# Magnetic resonance imaging anatomy of the rabbit brain at 3 T

**DOI:** 10.1186/s13028-015-0139-6

**Published:** 2015-08-28

**Authors:** Désirée Müllhaupt, Heinz Augsburger, Andrea Schwarz, Gregor Fischer, Patrick Kircher, Jean-Michel Hatt, Stefanie Ohlerth

**Affiliations:** Clinic of Diagnostic Imaging, Vetsuisse Faculty, University of Zurich, Winterthurerstrasse 285c, 8057 Zurich, Switzerland; Vetsuisse Faculty, Institute of Veterinary Anatomy, University of Zurich, Winterthurerstrasse 260, 8057 Zurich, Switzerland; Section of Anesthesiology, Vetsuisse Faculty, University of Zurich, Winterthurerstrasse 285c, 8057 Zurich, Switzerland; Laboratory Animal Services Center, University of Zurich, Winterthurerstrasse 190, 8057 Zurich, Switzerland; Clinic for Zoo Animals, Exotic Pets and Wildlife, Vetsuisse Faculty, University of Zurich, Winterthurerstrasse 260, 8057 Zurich, Switzerland

**Keywords:** MRI, Brain, Normal, Rabbit, 3 T

## Abstract

**Background:**

Rabbits are widely accepted as an animal model in neuroscience research. They also represent very popular pet animals, and, in selected clinical cases with neurological signs, magnetic resonance imaging (MRI) may be indicated for imaging the rabbit brain. Literature on the normal MRI anatomy of the rabbit brain and associated structures as well as related reference values is sparse. Therefore, it was the purpose of this study to generate an MRI atlas of the normal rabbit brain including the pituitary gland, the cranial nerves and major vessels by the use of a 3 T magnet.

**Results:**

Based on transverse, dorsal and sagittal T2-weighted (T2w) and pre- and post-contrast 3D T1-weighted (T1w) sequences, 60 intracranial structures were identified and labeled. Typical features of a lissencephalic brain type were described. In the 5 investigated rabbits, on T1w images a crescent-shaped hyperintense area caudodorsally in the pituitary gland most likely corresponded to a part of the *neurohypophysis*. The optic, trigeminal, and in part, the facial, vestibulocochlear and trochlear nerves were identified. Mild contrast enhancement of the trigeminal nerve was present in all rabbits. Absolute and relative size of the pituitary gland, midline area of the cranial and caudal cranial fossa and height of the *tel*- and *diencephalon*, 3rd and 4th ventricles were also determined.

**Conclusions:**

These data established normal MRI appearance and measurements of the rabbit brain. Results provide reference for research studies in rabbits and, in rare instances, clinical cases in veterinary medicine.

**Electronic supplementary material:**

The online version of this article (doi:10.1186/s13028-015-0139-6) contains supplementary material, which is available to authorized users.

## Background

Rabbits are widely accepted as an animal model in neuroscience research, and have been employed to study for example ischemic stroke [1], traumatic [2] and radiation brain injury [3], dementia [4] and intrauterine and postnatal neurodevelopment [5]. Rabbits are also very popular pet animals and are frequently presented to our hospital. Neurological diseases are common in rabbits [6]. Pasteurellosis or other bacterial infections and encephalitozoonosis causing encephalitis, as well as cerebral *larva migrans* leading to *encephalomalacia* are considered the most common conditions [7]. Magnetic resonance Imaging (MRI) is considered the gold standard to image the brain in humans and animals. Its availability for clinical use has dramatically increased in veterinary medicine over the years. However, costs and an increased risk of complications due to general anesthesia [8] are limiting factors for the application of MRI in exotic animals.

Currently, the rabbit brain and head MRI anatomy is available at reduced resolution from low field-strength (0.2 T) predominantly for clinical use [9]. Recently, an MRI atlas was published using a 7 T magnet in excised and fixed rabbit brains. However, T2w and post-contrast T1w sequences were not performed and the study lacks information on the pituitary gland, cranial nerves or vascular structures [10].

The aim of the present study was to build a comprehensive MRI atlas and MRI reference values of the normal brain and associated structures in rabbits. Results may serve as a basis for research and, in selected cases, as a clinical guide in rabbits with intracranial disease.

## Methods

### Animals

Five intact female New Zealand White rabbits were included in the study [6–7 months of age, body weight (BW) range 2.8–3.2 kg]. Animals were provided by the Laboratory Animal Services Center, University of Zurich and originated from a conventionally maintained animal facility with annual hygiene monitoring. The study was authorized by the animal care and use committee of the veterinary office of the Canton of Zurich (permit number 61/2012). Animal handling and all procedures were performed following the guidelines and regulations of the Animal Experimental Ethics Committee of the University of Zurich.

### MRI

All five rabbits underwent clinical examination prior to anesthesia and were judged clinically normal (ASA 1). Following sedation with fentanyl (5 µg/kg), midazolam (0.5 mg/kg) and medetomidin (200 µg/kg) given intramuscularly, a catheter was placed in the external auricular vein. After preoxygenation, the trachea was intubated with a cuffed endotracheal tube (4 animals) or a supraglottic airway device was placed (1 rabbit). Animals were connected to a non-rebreathing system. Swallowing during intubation attempts occurred in 1 rabbit, and intravenous propofol (0.68 mg/kg) was given. Anesthesia was maintained with isoflurane given to effect in an oxygen/air gas flow of 500 ml/kg/min with an initial inspired fraction of oxygen of 0.5. The rabbits were allowed to breath spontaneously. Lactated Ringer’s solution was infused at 10 ml/kg/h intravenously. Cardiovascular and respiratory variables were measured continuously with a multiparameter monitor and recorded. After the MRI scan, flumazenil (0.05 mg/kg; 5 rabbits) and atipamezole (0.25–0.5 mg/kg, 4 rabbits) were administered subcutaneously. All rabbits recovered uneventfully and were returned to their original barn the same day.

A 3 T magnet (Ingenia, Philips Medical Systems Nederland B.V., The Netherlands) was used in combination with an extremity coil (dS SmallExtremity 8ch, phased-array receive-only, 8 channels). Each rabbit was scanned in dorsal recumbency with the neck and head in an extended position. The following parameters were used for acquisition of the T2w turbo spin echo (TSE) transverse sequence: echo time (TE) = 100, repetition time (TR) = 5500, slice thickness was 2.2 mm with an interslice gap of 2.4 mm, voxel size = 0.4 mm, number of signal average (NSA) = 5, bandwidth (BW) = 354 Hz/pixel, echo train length = 13, and the field of view (FOV) = 100 mm. Dorsal and sagittal TSE T2w sequences as well as a transverse fluid attenuated inversion recovery (FLAIR longTR CLEAR) were also obtained (see Additional file 1: 2–4). For the pre- and postcontrast transverse T1w 3D (TFE SENSE) sequences, TE was 6.2, TR was 13.3, slice thickness was 0.6 mm without interslice gap, 0.6 mm isotropic resolution, NSA = 3, flip angle = 8°, BW = 114 Hz/pixel, echo train length = 166, and the FOV = 100 mm. As a contrast agent, Gadodiamidum 0.5 mmol/ml (0.3 ml/kg) was given intravenously and manually as a rapid bolus injection.

MRI slices were oriented as follows: transverse sections perpendicular to the ventral aspect of the brain, sagittal sections parallel with the interthalamic adhesion and dorsal sections parallel to the ventral aspect of the brain.

Image interpretation and measurements were done with dedicated software (OsiriX Open Source™ 5.0.2, OsiriX Foundation, Geneva). For the MRI atlas, constant window settings were used: T2w images with a window width (WW) of 400 and a window level (WL) of 170, and pre-contrast and post-contrast T1w images with a WW of 250, a WL of 100 and a WW of 350 and a WL of 150, respectively. Using T1w 3D (TFE SENSE) sequences with the possibility of acquiring very thin slices, more images were generated with T1w than with T2w sequences. Therefore, for the MRI atlas, multiplanar reconstructions of the transverse T1w images were also used to identify the transverse T1w images in a plane and at the anatomic level that matched best with T2w images. Anatomic structures were identified based on anatomy books of rabbits and Guinea pigs [11], a histological atlas of the rabbit brain and spinal cord [12], a previously published MRI-based atlas [10], a study of low field MRI of the rabbit head [9] and MRI brain atlases of dogs and cats [13, 14]. The anatomical structure nomenclature used in this study followed the format of the Nomina Anatomica Veterinaria [15].

**Fig. 1:**
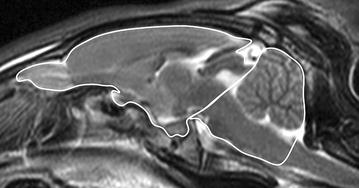
Mid-sagittal T2w image of the rabbit brain: the midline area of the caudal cranial fossa was defined as the area limited caudally by the *foramen magnum* and cranially by the rostral contour of the *cerebellum* and the dorsum *sella turcica*. The midline area of the cranial cranial fossa included the olfactory bulb, following the dorsal brain surface until the caudal pole, then following the *tectum mesencephali* to the *dorsum sella turcica*, going rostrally to the olfactory bulb again, including the pituitary gland and the optic chiasm

**Fig. 2:**
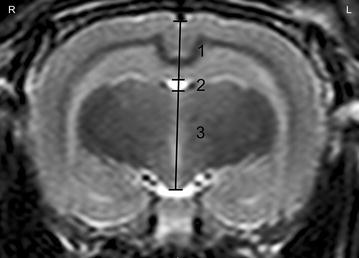
Transverse T2w image of the *diencephalon* of the rabbit brain including the third ventricle dorsal and ventral to it: telencephalic height (*1*), third ventricular height (*2*) and diencephalic height (*3*) were assessed along the midline

A variety of measurements were performed. In mid-sagittal T2w images, the midline area of the caudal cranial fossa was defined as the area limited caudally by the *foramen magnum* and cranially by the rostral contour of the *cerebellum* and the *dorsum sella turcica*. The midline area of the cranial cranial fossa included the olfactory bulb, following the dorsal brain surface until the caudal pole, then following the *tectum mesencephali* to the *dorsum sella turcica*, going rostrally to the olfactory bulb again, including the pituitary gland and the optic chiasm. The sum of both, the midline area of the cranial and caudal cranial fossa, defined the total midline braincase area (Fig. 1). On a transverse T2w image of the *diencephalon* including the third ventricle dorsal and ventral to it, brain height, telencephalic height, third ventricular height and diencephalic height were assessed along the midline (Fig. 2). Most of the images contained the pituitary gland. When the third ventricle was present dorsal and ventral to the *diencephalon* in more than two images, the largest measurement was used. Brain height was measured in the center, from the ventral border of the hypothalamus (mammillary bodies) to the ventral border of the longitudinal fissure. The height of the third ventricle was assessed in its dorsal part, dorsal to the *thalamus*.

**Fig. 3:**
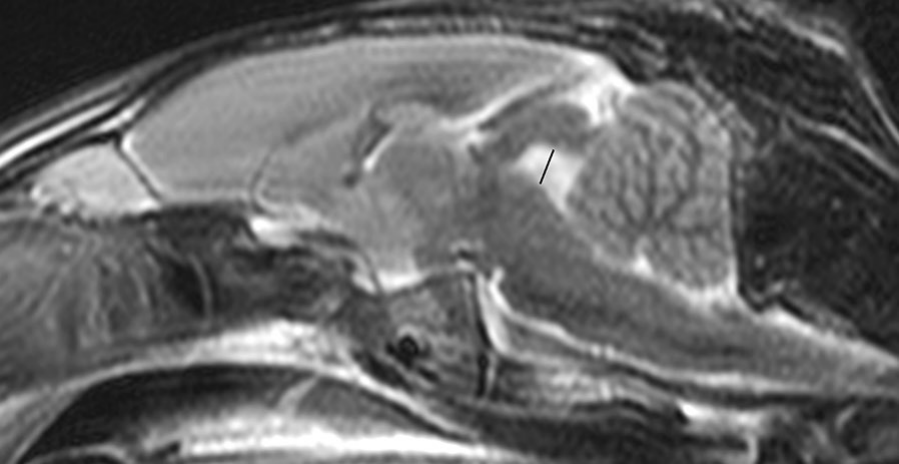
Mid-sagittal T2w image of the rabbit brain: fourth ventricular height was measured perpendicular to the base of the skull through the center of the fourth ventricle (*black line*)

Height, width and length of the pituitary gland and the transverse area of the brain were measured on pre-contrast T1w 3D images. The dorsal plane (pituitary length) was aligned parallel to the base of the skull. The transverse plane (pituitary height and width) was chosen perpendicular to the dorsal plane through the pituitary gland. Measurements were performed on the images presenting the largest dimensions of the pituitary gland. In the same image, brain area was measured excluding the pituitary gland. Then, the ratio of pituitary height/brain area was calculated. Fourth ventricular height was measured in a mid-sagittal T2w image perpendicular to the base of the skull through the center of the fourth ventricle (Fig. 3).

Descriptive statistics were calculated using the SPSS statistics program (Version 19, IBM Corporation, Armonk, NY, USA).

## Results

**Fig. 4:**
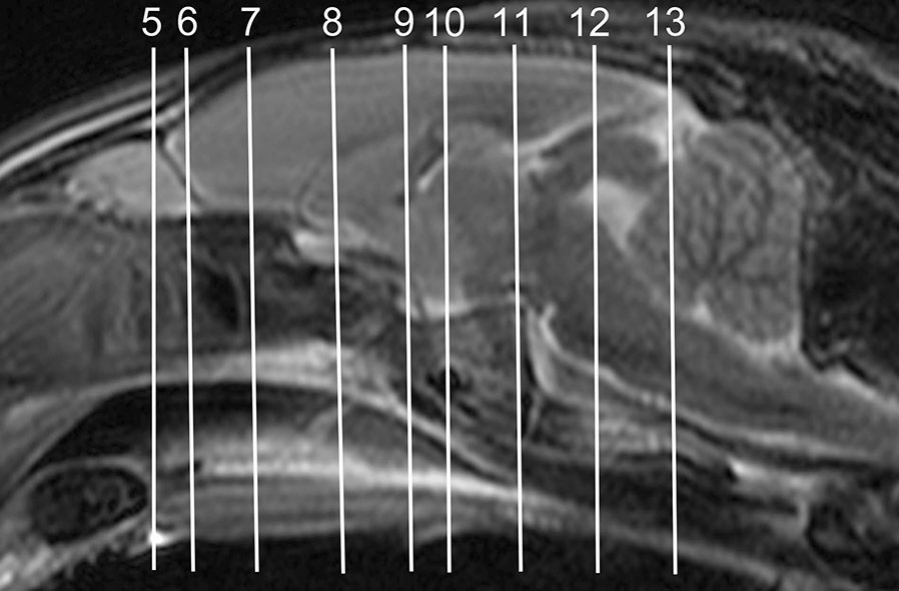
Mid-sagittal T2w reference image of the rabbit brain: the *vertical lines* indicate the level of the transverse images in Figs. 5, 6, 7, 8, 9, 10, 11, 12 and 13

**Fig. 5:**
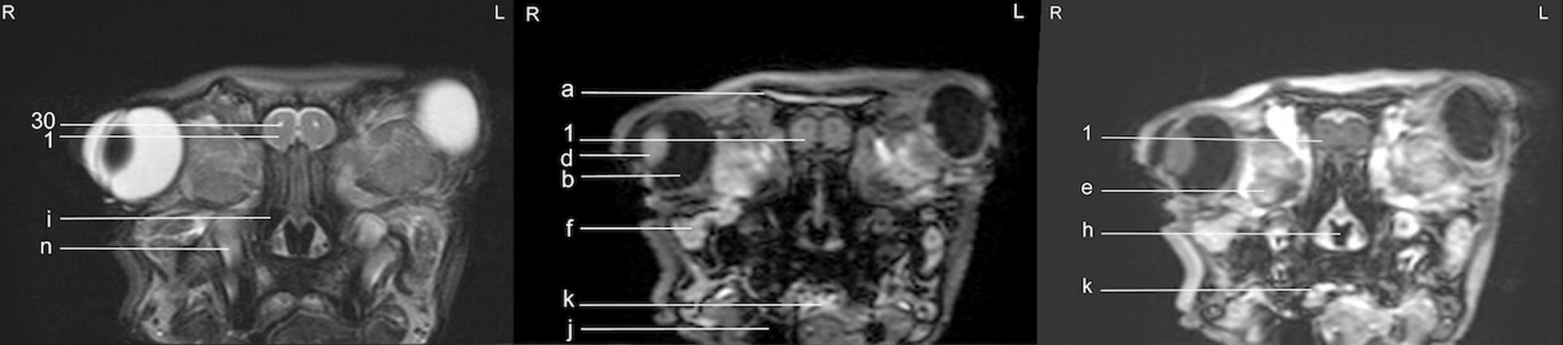
Transverse images of the rabbit brain at the level of the olfactory bulb (*left* T2w; *middle* T1w; *right* T1w post-contrast)

**Fig. 6:**
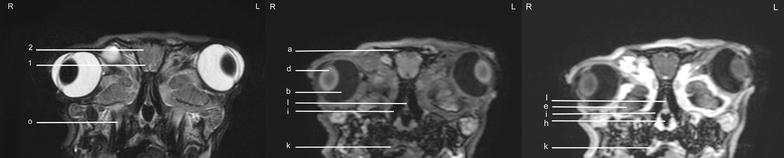
Transverse images of the rabbit brain at the level of the olfactory bulb/rostral *telencephalon* (*left* T2w; *middle* T1w; *right* T1w post-contrast)

**Fig. 7:**
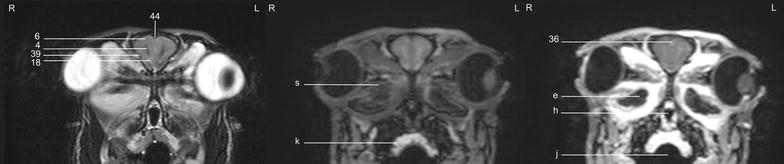
Transverse images of the rabbit brain at the level of the rostral *telencephalon*/rhinal fissure (*left* T2w; *middle* T1w; *right* T1w post-contrast)

**Fig. 8:**
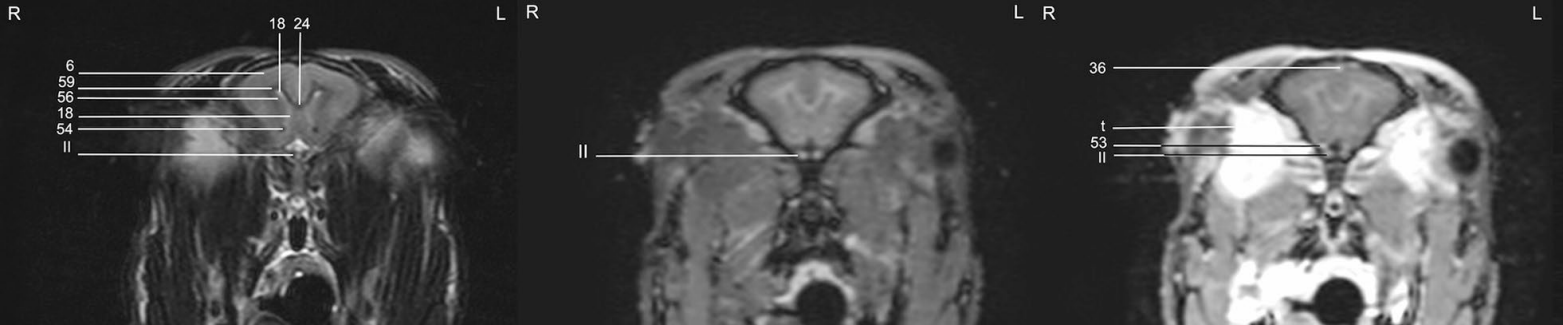
Transverse images of the rabbit brain at the level of the mid *telencephalon* (*left* T2w; *middle* T1w; *right* T1w post-contrast)

**Fig. 9:**
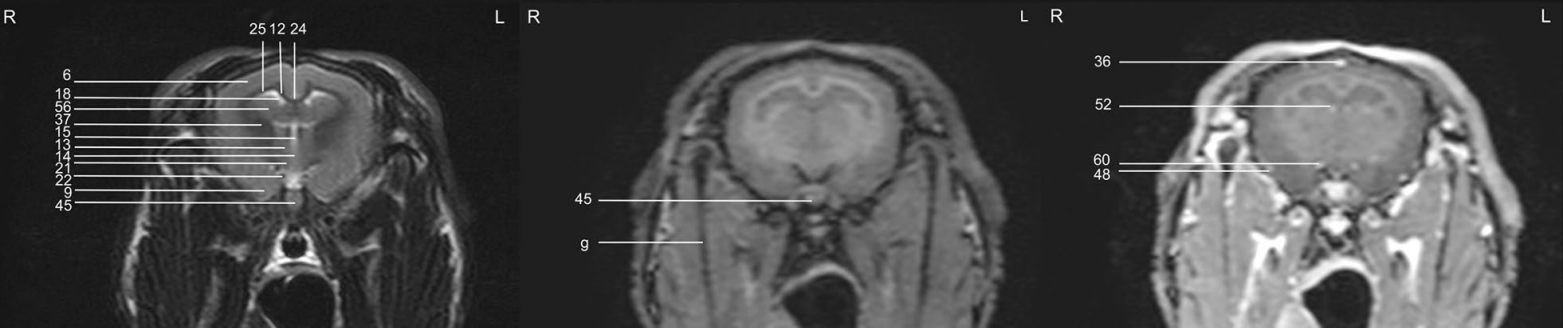
Transverse images of the rabbit brain at the level of the rostral part of the *hypophysis* (*left* T2w; *middle* T1w; *right* T1w post-contrast)

**Fig. 10:**
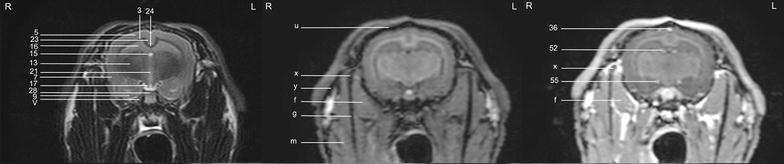
Transverse images of the rabbit brain at the level of the caudal part of the *hypophysis* (*left* T2w; *middle* T1w; *right* T1w post-contrast)

**Fig. 11:**
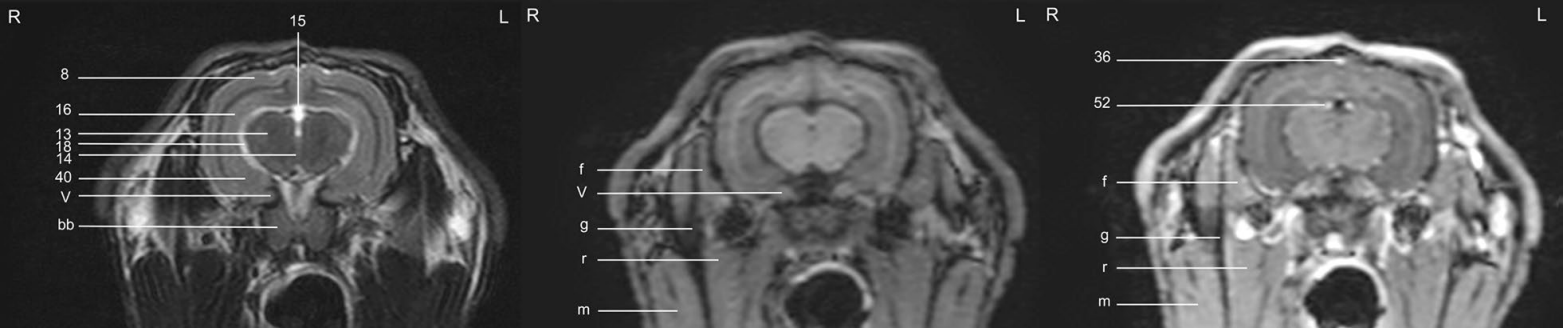
Transverse images of the rabbit brain at the level of the *thalamus* (*left* T2w; *middle* T1w; *right* T1w post-contrast)

**Fig. 12:**
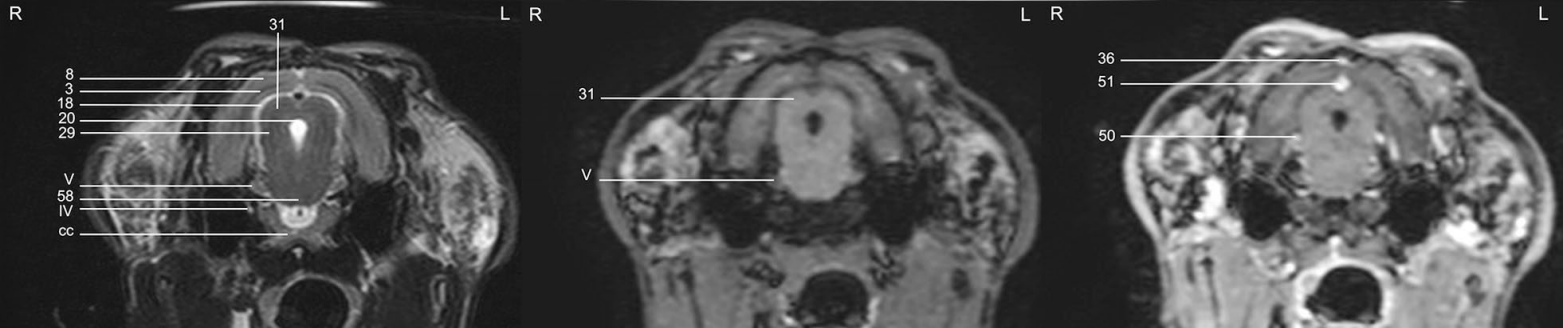
Transverse images of the rabbit brain at the level of the mesencephalic aqueduct (*left* T2w; *middle* T1w; *right* T1w post-contrast)

**Fig. 13:**
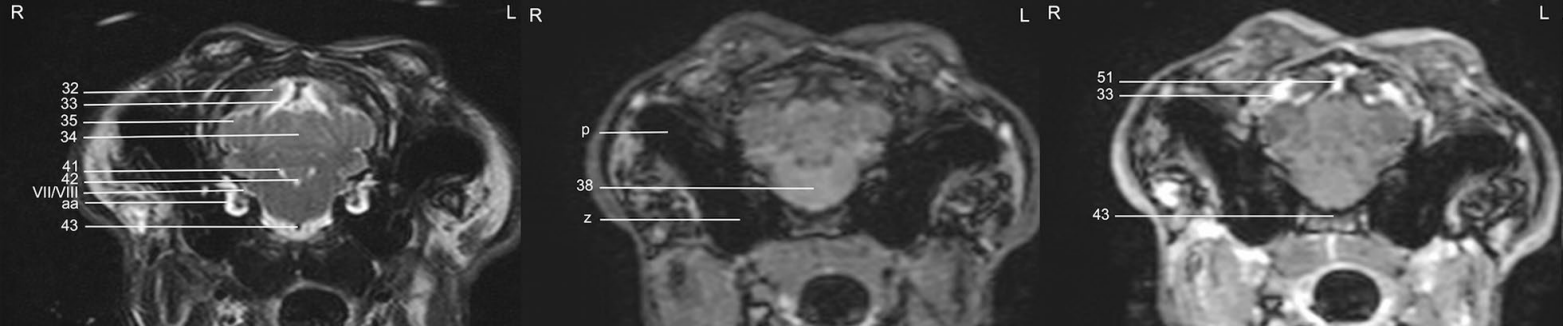
Transverse images of the rabbit brain at the level of the rostral *cerebellum* (*left* T2w; *middle* T1w; *right* T1w post-contrast)

In general, no significant anatomic differences were diagnosed subjectively in the 5 rabbits, and presented structures appeared normal in all sequences. Transverse T2w images included 24 or 25 sections from the cribriform plate to the cranial aspect of the atlas. Nine representative transverse T2w images were defined at different levels (reference sagittal scan, Fig. 4) and corresponding transverse pre- and post-contrast T1w images (Figs. 5, 6, 7, 8, 9, 10, 11, 12, 13) were labeled according to an English and Latin index of structures (Table 1). A complete MRI study of one rabbit brain including all images of all sequences is provided as Additional file 1: 1–6.

**Table 1:** Index of structures

No.	English name	Latin name
1.	Olfactory bulb	*Bulbus olfactorius*
2.	Rostral pole of neopallium	*Polus rostralis neopallii*
3.	Subcortical white matter	*Substantia alba subcorticale*
4.	Periventricular white matter	*Substantia alba periventriculare*
5.	Gray matter	*Substantia grisea*
6.	Frontal cortex	*Cortex frontalis*
7.	Temporal cortex	*Cortex temporalis*
8.	Parietal cortex	*Cortex parietalis*
9.	Piriform cortex	*Cortex piriformis*
10.		*Adenohypophysis*
11.		*Neurohypophysis*
12.		*Truncus corporis callosi*
13.		*Thalamus*
14.	Interthalamic adhesion	*Adhesio interthalamica*
15.	Third ventricle	*Ventriculus tertius*
16.		*Hippocampus*
17.	Mamillary body	*Corpus mamillare*
18.	Lateral ventricle	*Ventriculus lateralis*
19.	Midbrain tectum	*Tectum mesencephali*
20.	Cerebral aqueduct	*Aquaeductus mesencephali*
21.		*Hypothalamus*
22.	Optical tract	*Tractus opticus*
23.	Cingulate cortex	*Cortex cingularis*
24.		*Corpus callosum*
25.		*Corona radiata*
26.	Optic chiasm	*Chiasma opticum*
27.		*Cisterna magna*
28.	Pituitary stalk	*Infundibulum*
29.	Caudal colliculus	*Colliculus caudalis*
30.	Olfactory recess of lateral ventricle	*Recessus olfactorius*
31.	Rostral colliculus	*Colliculus rostralis*
32.	Caudal pole	*Polus caudalis neopallii*
33.	Transverse sinus	*Sinus transversus*
34.	Rostral cerebellar lobe	*Lobus rostralis cerebelli*
35.	Ansiform lobule	*Lobulus ansiformis*
36.	Dorsal sagittal sinus	*Sinus sagittalis dorsalis*
37.	Internal capsule	*Capsula interna*
38.		*Medulla oblongata*
39.	Rhinal fissure	*Fissura rhinalis*
40.	Piriform lobe	*Lobus piriformis*
41.	Lateral recess of the fourth ventricle	*Recessus lateralis ventriculi quarti*
42.	Fourth ventricle	*Ventriculus quartus*
43.	Basilar artery	*Arteria basilaris*
44.	Longitudinal cerebral fissure	*Fissura longitudinalis cerebri*
45.	Pituitary gland	*Hypophysis*
46.		*Cerebellum*
47.	Spinal cord	*Medulla spinalis*
48.	Medial cerebral artery (and cortical branches)	*Arteria cerebri media*
49.		*Genu corporis callosi*
50.	Caudal communicating artery	*Arteria communicans caudalis*
51.	Caudal cerebral artery	*Arteria cerebri caudalis*
52.	Internal cerebral vein	*Vena interna cerebri*
53.	Ophthalmic vein	*Vena ophthalmica*
54.	Rostral commissure	*Commissura rostralis cerebri*
55.	Caudal cerebral artery	*Arteria cerebri caudalis*
56.	Caudate nucleus	*Nucleus caudatus*
57.		*Septum pellucidum*
58.		*Pons*
59.		*Radiatio corporis callosi*
60.	Rostral cerebral artery	*Arteria cerebri rostralis*
(a)	Frontal bone	*Os frontale*
(b)	Vitreous humour	*Corpus vitreum*
(c)		*Sclera*
(d)		*Lens*
(e)	Zygomatic gland	*Glandula zygomatica*
(f)	Lateral pterygoid muscle	*Musculus pterygoideus lateralis*
(g)	Mandible	*Mandibula*
(h)		*Nasopharynx*
(i)	Sphenoidal sinus	*Sinus sphenoidalis*
(j)		*Oropharynx*
(k)	Soft palate	*Palatum molle*
(l)	Presphenoid bone	*Os praesphenoidale*
(m)	Masseter muscle	*Musculus masseter*
(n)	First upper molar tooth	
(o)	Second upper molar tooth	
(p)	External acustic meatus	*Meatus acusticus externus*
(q)	Ethmoid bone	*Os ethmoidale*
(r)	Medial pterygoid muscle	*Musculus pterygoideus medialis*
(s)	Extraocular muscles	*Musculi recti et obliqui*
(t)	Adipose body of the orbit	*Corpus adiposum orbitae*
(u)	Parietal bone	*Os parietale*
(v)		*Dorsum sellae*
(w)	Presphenoid bone	*Os praesphenoidale*
(x)	Condyloid process	*Processus condylaris (Mandibula)*
(y)	Zygomatic bone	*Os zygomaticum*
(z)	Tympanic cavity	*Cavum tympani*
(aa)	Peri- and endolymph	*Perilympha et endolympha cochleae*
(bb)	Basisphenoid bone	*Os basisphenoidale*
(cc)	Basioccipital bone	*Os basioccipitale*
II.	Optic nerve	*Nervus opticus*
IV.	Trochlear nerve	*Nervus trochlearis*
V.	Trigeminal nerve	*Nervus trigeminus*
VII.	Facial nerve	*Nervus facialis*
VIII.	Vestibulocochlear nerve	*Nervus vestibulocochlearis*

Because more transverse T1w images were acquired than T2w images (different slice thickness), volume averaging was more evident on T2w images causing mildly different presentation of anatomic structures in the different sequences. Eye movements were present in one rabbit causing mild motion artifacts at the level of the olfactory bulb.

Cerebrospinal fluid was hyperintense in T2w images and enabled identification of the 3rd ventricle, the aqueduct, the 4th ventricle, the subarachnoid space and both optic nerves. The lateral ventricles were symmetrical and narrow. They almost communicated ventrally at the level of the *diencephalon* e.g. the interthalamic adhesion (Fig. 11). Their caudal recesses were well seen at the level of the *mesencephalon* (Fig. 12), and the rostral recesses were identified at the level of the rostral portion of the *diencephalon* just dorsal to the caudate nucleus (Figs. 8, 9) with a little cavity extending into the olfactory bulb (Fig. 5). The longitudinal and rhinal fissures were also clearly identified. The *tectum mesencephali* with its rostral and caudal colliculi was precisely visible. Considering the rabbit’s brain size, MRI studies of the herein investigated animals showed good spatial resolution. Contrast between hypointense white matter areas (periventricular and subcortical white matter, internal and external capsule, *corona radiata*, *corpus callosum*, anterior commissure) and the more signal intense gray matter regions such as the cerebral cortex and the *hippocampus*, was good in T2w sequences and was considered mildly superior to FLAIR images. On pre- and post-contrast T1w images, signal intensity of white matter was more hyperintense than grey matter, and contrast also was of good diagnostic quality. The *cerebellum* was of a triangular shape in the mid-sagittal plane and there was moderate contrast between gray and white matter on T2w images. The cerebellar tentorium was rather flat and short.

The optic nerves could be followed easily from the sclera through the optic canal to the optic chiasm just rostral to the hypophyseal infundibulum and finally to the brain as the optic tracts (Figs. 9, 10). Because the optic nerve represents a white matter tract and is surrounded by a dural sheath containing cerebrospinal fluid, it was hyperintense in T1w images and hypointense with a hyperintense rim in T2w images (Fig. 8).

The trigeminal nerve was the largest cranial nerve and visible on 5 consecutive transverse T2w images from its origin lateral at the *pons* to the level of the pituitary gland (Figs. 10, 11, 12). In all rabbits, there was moderate contrast enhancement of the trigeminal nerve, and intensity was subjectively less than that of the pituitary gland. The ophthalmic branch was the only branch identified, while passing through the orbital fissure. The maxillary and mandibular branches were not clearly depicted. The vestibulocochlear nerve and the facial nerve leaving the pons could not be differentiated from each other in any sequence. They passed through the internal acoustic meatus just dorsal to the cochlea to the inner ear and were best seen in T2w images (Fig. 13). The emergence of the vagal group (glossopharyngeal, vagal and accessory nerves) could only be addressed moderately in T1w sequences at the level of the caudal cerebellar peduncle (not shown). Because of their close origin and their small size, these cranial nerves could not be differentiated from each other. Although the origin of the trochlear nerve was not identified, it was depicted on its course ventrally to the trigeminal nerve and mediodorsally to the tympanic bulla on T2w images. The oculomotor, abducent and hypoglossal nerves could not be identified at all.

Altogether, 60 structures were identified and labeled within the cranial fossa. Additionally, numerous bony and soft-tissue structures were identified and labeled on T1w images. The cerebral vasculature of the rabbit brain was also identified on post-contrast T1w images with clear resolution of the major arteries and veins.

**Fig. 14:**
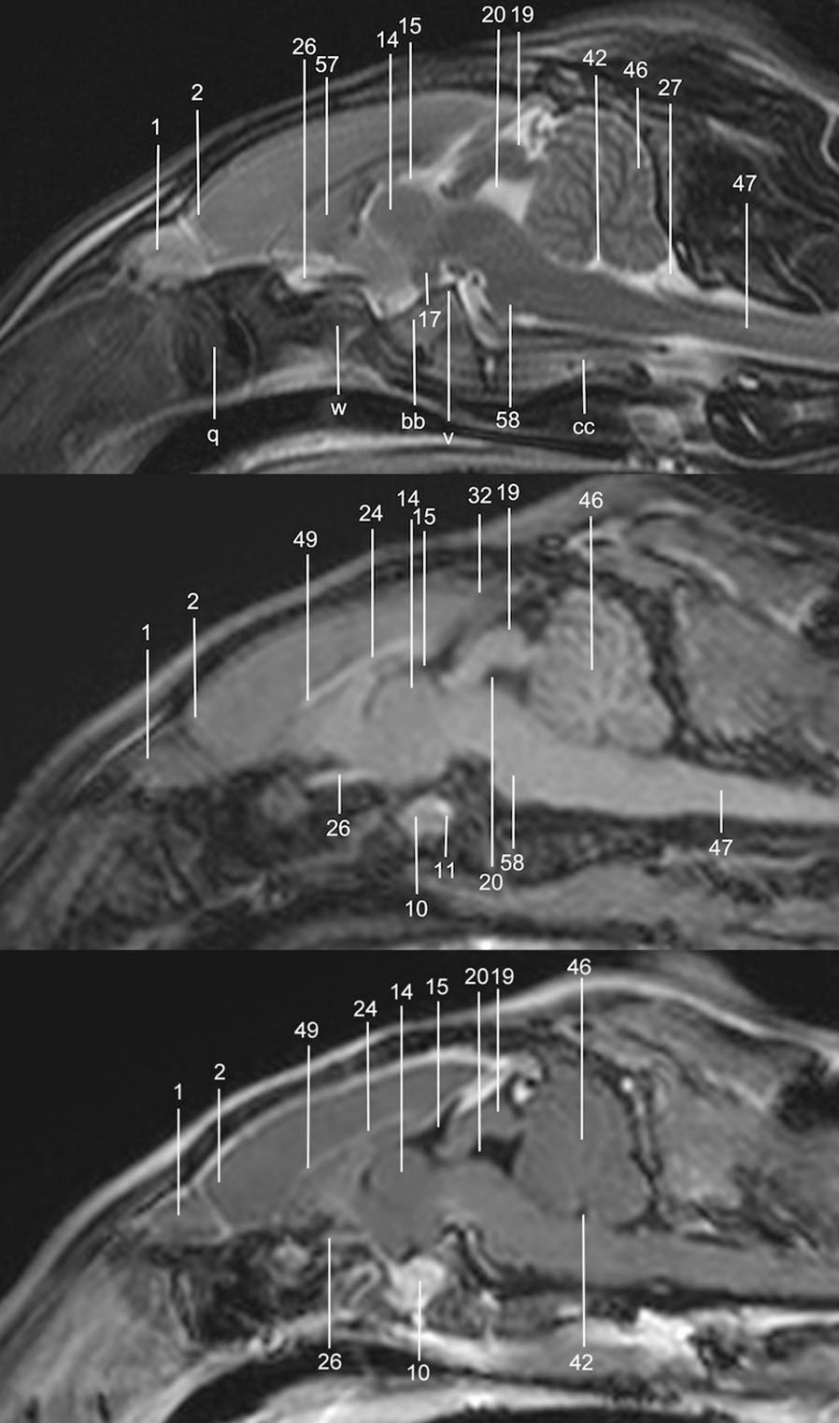
Mid-sagittal image of the rabbit brain (*top* T2w; *middle* T1w; *bottom* T1w post-contrast). Rostral is to the left and caudal to the right

Descriptive statistics for the performed measurements are shown in Table 2. In general, brain size was considered small. The height of the *telencephalon* was small in relation to the *diencephalon*. The pituitary gland was of a round to ovoid shape and easily identified in all rabbits in the *sella turcica*, which demonstrated a very prominent *dorsum sellae* of the basisphenoid bone (Fig. 14). Additionally, in T1w transverse and sagittal images, the infundibular recess with the *infundibulum* was located dorsorostrally to the pituitary gland, and a crescent-shaped area of high signal intensity was found caudodorsally in the pituitary gland of all animals. The pituitary gland in relation to the brain area appeared relatively large. The pituitary gland was best identified in pre-contrast T1 images. After administration of contrast medium, differentiation from the surrounding vessels e.g. the cavernous sinus and the caudal communicating artery, was difficult.

**Table 2:** MRI measurements of the brain and the pituitary gland in five healthy rabbits

Variable	Mean	SD	Median	Minimum, maximum
Midline area of the cranial cranial fossa (mm^2^)	409.3	17.3	409.7	382.6–429.9
Midline area of the caudal cranial fossa (mm^2^)	229.8	13.1	233.3	206.9–239.2
Total midline braincase area (mm^2^)	639.1	20.1	643.0	615.6–666.5
Diencephalic height (mm)	8.8	0.58	8.8	8.2–9.7
Brain height (mm)	15.5	0.77	15.2	14.7–16.5
Telencephalic height (mm)	5.9	0.35	5.89	5.4–6.3
3rd ventricular height (mm)	0.88	0.11	0.90	0.72–1.02
4th ventricular height (mm)	2.4	0.23	2.47	2.1–2.7
Pituitary gland height (mm)	3.5	0.26	3.5	3.3–4.5
Pituitary gland width (mm)	3.5	0.59	3.3	3.0–4.5
Pituitary gland length (mm)	5.0	0.34	4.8	4.7–5.5
Transverse brain area (mm^2^)	492.5	13.8	497.2	473.4–506.1
Ratio pituitary gland height/brain area (mm^−1^)	0.72	0.04	0.72	0.66–0.76

## Discussion

The rabbit brain is counted among the lissencephalic (smooth) brain type in contrast to the gyrencephalic (convoluted) brain type. The amount of fissures in the brain i.e. gyrification, is related to both, size of the animal and size of the brain. With gyrification, telencephalic surface and weight, respectively size increase and, therefore, number of cortical neurons increase [16]. Subjective image analysis and objective measurements of the present study also demonstrated a rather large *di*-*, mes*- and *metencephalon* in relation to a smaller *telencephalon*. Typical anatomical features of the rabbit brain and cranial fossa were seen, e.g. the funnel-shaped mesencephalic aqueduct with the widest diameter being rostral [17], the narrow but long lateral ventricles which almost communicated ventrally and extended rostrally to the olfactory bulb [17] and the short and flat cerebellar tentorium [18].

Similar to humans and the dog, a crescent-shaped hyperintense area was noted caudodorsally in the pituitary gland on T1w images most likely corresponding to a part of the *neurohypophysis*. Absolute and relative measurements of the rabbit’s pituitary gland were much higher than for example in the dog [19].

Similar to the dog [20], enhancement of the trigeminal nerve was found in all five rabbits without any clinical evidence of trigeminal nerve disease. According to the literature, enhancement is thought to occur due to a lack of a blood nerve barrier in the external nerve sheaths of the rabbit’s trigeminal nerve [21]. However, a post mortem examination was not performed in any rabbit of the present study, and therefore, a perineural plexus causing contrast enhancement cannot be ruled out.

A 3 T high-field magnet in combination with an extremity coil was used in the present study, which represents the highest field strength currently used in veterinary practice. In comparison to a recently performed study of the rabbit head with a low-field MRI unit [9], identification of 60 structures within the rabbit cranial fossa were identified, including the major cortical, gray and white matter regions as well as the major regions of the *di*-*, mes*- and *metencephalon* and major vessels. However, smaller regions within the cortex or deep gray matter as well as several smaller cranial nerves were difficult or even impossible to define. Although spatial and contrast resolution was considered to be of good quality in the present study, it may have been improved by the use of e.g. a microscopy coil and newer sequences such as 3D options for T2w and FLAIR sequences. However, these coils and sequences were not available at the time of the present study.

In conclusion, the present study established normal MRI appearance and MRI reference values of the rabbit brain. Results provide reference for research studies in rabbits and, in rare instances, clinical cases in veterinary medicine.

## Additional file

Additional file 1:
**1.** Complete image series of a transverse TSE T2w sequence in one rabbit. **2.** Complete image series of a sagittal TSE T2w sequence in the same rabbit as in Additional file 1: 1. **3.** Complete image series of a dorsal TSE T2w sequence in the same rabbit as in Additional file 1: 1. **4.** Complete image series of a FLAIR longTR CLEAR sequence in the same rabbit as in Additional file 1: 1. **5.** Complete image series of a precontrast T1w 3D (TFE SENSE) sequence in the same rabbit as in Additional file 1: 1. **6.** Complete image series of a postcontrast T1w 3D (TFE SENSE) sequence in the same rabbit as in Additional file 1: 1.
