# Supplementary figures and images for: Magnetic resonance imaging anatomy of the rabbit brain at 3 T (part 2 of 2)
Source: Acta Vet Scand. 2015 Aug 28;57(1):47. doi: 10.1186/s13028-015-0139-6 (PMC4551377; doi:10.1186/s13028-015-0139-6)

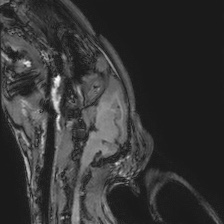

Supplement: Additional file 1: — 1. Complete image series of a transverse TSE T2w sequence in one rabbit. 2. Complete image series of a sagittal TSE T2w sequence in the same rabbit as in Additional file 1: 1. 3. Complete image series of a dorsal TSE T2w sequence in the same rabbit as in Additional file 1: 1. 4. Complete image series of a FLAIR longTR CLEAR sequence in the same rabbit as in Additional file 1: 1. 5. Complete image series of a precontrast T1w 3D (TFE SENSE) sequence in the same rabbit as in Additional file 1: 1. 6. Complete image series of a postcontrast T1w 3D (TFE SENSE) sequence in the same rabbit as in Additional file 1: 1. [file 13028_2015_139_MOESM1_ESM.zip › Brain_2200809/sT1W_3D_TFE_701/IM-0005-0081.jpg]

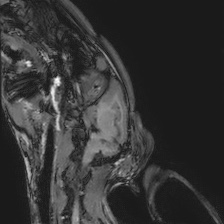

Supplement: Additional file 1: — 1. Complete image series of a transverse TSE T2w sequence in one rabbit. 2. Complete image series of a sagittal TSE T2w sequence in the same rabbit as in Additional file 1: 1. 3. Complete image series of a dorsal TSE T2w sequence in the same rabbit as in Additional file 1: 1. 4. Complete image series of a FLAIR longTR CLEAR sequence in the same rabbit as in Additional file 1: 1. 5. Complete image series of a precontrast T1w 3D (TFE SENSE) sequence in the same rabbit as in Additional file 1: 1. 6. Complete image series of a postcontrast T1w 3D (TFE SENSE) sequence in the same rabbit as in Additional file 1: 1. [file 13028_2015_139_MOESM1_ESM.zip › Brain_2200809/sT1W_3D_TFE_701/IM-0005-0082.jpg]

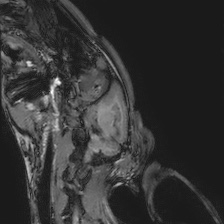

Supplement: Additional file 1: — 1. Complete image series of a transverse TSE T2w sequence in one rabbit. 2. Complete image series of a sagittal TSE T2w sequence in the same rabbit as in Additional file 1: 1. 3. Complete image series of a dorsal TSE T2w sequence in the same rabbit as in Additional file 1: 1. 4. Complete image series of a FLAIR longTR CLEAR sequence in the same rabbit as in Additional file 1: 1. 5. Complete image series of a precontrast T1w 3D (TFE SENSE) sequence in the same rabbit as in Additional file 1: 1. 6. Complete image series of a postcontrast T1w 3D (TFE SENSE) sequence in the same rabbit as in Additional file 1: 1. [file 13028_2015_139_MOESM1_ESM.zip › Brain_2200809/sT1W_3D_TFE_701/IM-0005-0083.jpg]

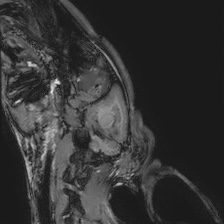

Supplement: Additional file 1: — 1. Complete image series of a transverse TSE T2w sequence in one rabbit. 2. Complete image series of a sagittal TSE T2w sequence in the same rabbit as in Additional file 1: 1. 3. Complete image series of a dorsal TSE T2w sequence in the same rabbit as in Additional file 1: 1. 4. Complete image series of a FLAIR longTR CLEAR sequence in the same rabbit as in Additional file 1: 1. 5. Complete image series of a precontrast T1w 3D (TFE SENSE) sequence in the same rabbit as in Additional file 1: 1. 6. Complete image series of a postcontrast T1w 3D (TFE SENSE) sequence in the same rabbit as in Additional file 1: 1. [file 13028_2015_139_MOESM1_ESM.zip › Brain_2200809/sT1W_3D_TFE_701/IM-0005-0084.jpg]

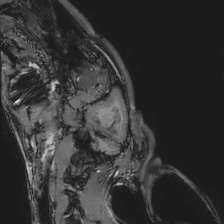

Supplement: Additional file 1: — 1. Complete image series of a transverse TSE T2w sequence in one rabbit. 2. Complete image series of a sagittal TSE T2w sequence in the same rabbit as in Additional file 1: 1. 3. Complete image series of a dorsal TSE T2w sequence in the same rabbit as in Additional file 1: 1. 4. Complete image series of a FLAIR longTR CLEAR sequence in the same rabbit as in Additional file 1: 1. 5. Complete image series of a precontrast T1w 3D (TFE SENSE) sequence in the same rabbit as in Additional file 1: 1. 6. Complete image series of a postcontrast T1w 3D (TFE SENSE) sequence in the same rabbit as in Additional file 1: 1. [file 13028_2015_139_MOESM1_ESM.zip › Brain_2200809/sT1W_3D_TFE_701/IM-0005-0085.jpg]

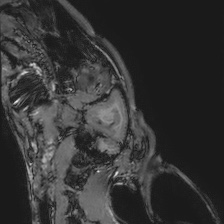

Supplement: Additional file 1: — 1. Complete image series of a transverse TSE T2w sequence in one rabbit. 2. Complete image series of a sagittal TSE T2w sequence in the same rabbit as in Additional file 1: 1. 3. Complete image series of a dorsal TSE T2w sequence in the same rabbit as in Additional file 1: 1. 4. Complete image series of a FLAIR longTR CLEAR sequence in the same rabbit as in Additional file 1: 1. 5. Complete image series of a precontrast T1w 3D (TFE SENSE) sequence in the same rabbit as in Additional file 1: 1. 6. Complete image series of a postcontrast T1w 3D (TFE SENSE) sequence in the same rabbit as in Additional file 1: 1. [file 13028_2015_139_MOESM1_ESM.zip › Brain_2200809/sT1W_3D_TFE_701/IM-0005-0086.jpg]

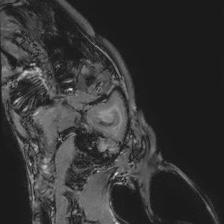

Supplement: Additional file 1: — 1. Complete image series of a transverse TSE T2w sequence in one rabbit. 2. Complete image series of a sagittal TSE T2w sequence in the same rabbit as in Additional file 1: 1. 3. Complete image series of a dorsal TSE T2w sequence in the same rabbit as in Additional file 1: 1. 4. Complete image series of a FLAIR longTR CLEAR sequence in the same rabbit as in Additional file 1: 1. 5. Complete image series of a precontrast T1w 3D (TFE SENSE) sequence in the same rabbit as in Additional file 1: 1. 6. Complete image series of a postcontrast T1w 3D (TFE SENSE) sequence in the same rabbit as in Additional file 1: 1. [file 13028_2015_139_MOESM1_ESM.zip › Brain_2200809/sT1W_3D_TFE_701/IM-0005-0087.jpg]

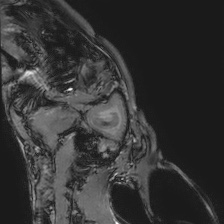

Supplement: Additional file 1: — 1. Complete image series of a transverse TSE T2w sequence in one rabbit. 2. Complete image series of a sagittal TSE T2w sequence in the same rabbit as in Additional file 1: 1. 3. Complete image series of a dorsal TSE T2w sequence in the same rabbit as in Additional file 1: 1. 4. Complete image series of a FLAIR longTR CLEAR sequence in the same rabbit as in Additional file 1: 1. 5. Complete image series of a precontrast T1w 3D (TFE SENSE) sequence in the same rabbit as in Additional file 1: 1. 6. Complete image series of a postcontrast T1w 3D (TFE SENSE) sequence in the same rabbit as in Additional file 1: 1. [file 13028_2015_139_MOESM1_ESM.zip › Brain_2200809/sT1W_3D_TFE_701/IM-0005-0088.jpg]

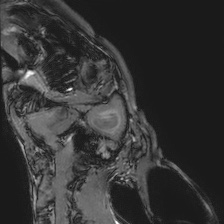

Supplement: Additional file 1: — 1. Complete image series of a transverse TSE T2w sequence in one rabbit. 2. Complete image series of a sagittal TSE T2w sequence in the same rabbit as in Additional file 1: 1. 3. Complete image series of a dorsal TSE T2w sequence in the same rabbit as in Additional file 1: 1. 4. Complete image series of a FLAIR longTR CLEAR sequence in the same rabbit as in Additional file 1: 1. 5. Complete image series of a precontrast T1w 3D (TFE SENSE) sequence in the same rabbit as in Additional file 1: 1. 6. Complete image series of a postcontrast T1w 3D (TFE SENSE) sequence in the same rabbit as in Additional file 1: 1. [file 13028_2015_139_MOESM1_ESM.zip › Brain_2200809/sT1W_3D_TFE_701/IM-0005-0089.jpg]

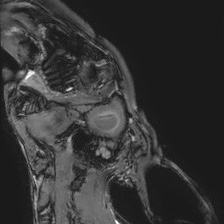

Supplement: Additional file 1: — 1. Complete image series of a transverse TSE T2w sequence in one rabbit. 2. Complete image series of a sagittal TSE T2w sequence in the same rabbit as in Additional file 1: 1. 3. Complete image series of a dorsal TSE T2w sequence in the same rabbit as in Additional file 1: 1. 4. Complete image series of a FLAIR longTR CLEAR sequence in the same rabbit as in Additional file 1: 1. 5. Complete image series of a precontrast T1w 3D (TFE SENSE) sequence in the same rabbit as in Additional file 1: 1. 6. Complete image series of a postcontrast T1w 3D (TFE SENSE) sequence in the same rabbit as in Additional file 1: 1. [file 13028_2015_139_MOESM1_ESM.zip › Brain_2200809/sT1W_3D_TFE_701/IM-0005-0090.jpg]

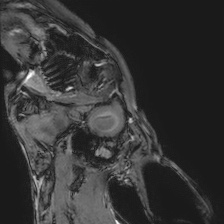

Supplement: Additional file 1: — 1. Complete image series of a transverse TSE T2w sequence in one rabbit. 2. Complete image series of a sagittal TSE T2w sequence in the same rabbit as in Additional file 1: 1. 3. Complete image series of a dorsal TSE T2w sequence in the same rabbit as in Additional file 1: 1. 4. Complete image series of a FLAIR longTR CLEAR sequence in the same rabbit as in Additional file 1: 1. 5. Complete image series of a precontrast T1w 3D (TFE SENSE) sequence in the same rabbit as in Additional file 1: 1. 6. Complete image series of a postcontrast T1w 3D (TFE SENSE) sequence in the same rabbit as in Additional file 1: 1. [file 13028_2015_139_MOESM1_ESM.zip › Brain_2200809/sT1W_3D_TFE_701/IM-0005-0091.jpg]

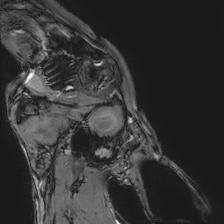

Supplement: Additional file 1: — 1. Complete image series of a transverse TSE T2w sequence in one rabbit. 2. Complete image series of a sagittal TSE T2w sequence in the same rabbit as in Additional file 1: 1. 3. Complete image series of a dorsal TSE T2w sequence in the same rabbit as in Additional file 1: 1. 4. Complete image series of a FLAIR longTR CLEAR sequence in the same rabbit as in Additional file 1: 1. 5. Complete image series of a precontrast T1w 3D (TFE SENSE) sequence in the same rabbit as in Additional file 1: 1. 6. Complete image series of a postcontrast T1w 3D (TFE SENSE) sequence in the same rabbit as in Additional file 1: 1. [file 13028_2015_139_MOESM1_ESM.zip › Brain_2200809/sT1W_3D_TFE_701/IM-0005-0092.jpg]

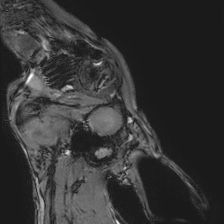

Supplement: Additional file 1: — 1. Complete image series of a transverse TSE T2w sequence in one rabbit. 2. Complete image series of a sagittal TSE T2w sequence in the same rabbit as in Additional file 1: 1. 3. Complete image series of a dorsal TSE T2w sequence in the same rabbit as in Additional file 1: 1. 4. Complete image series of a FLAIR longTR CLEAR sequence in the same rabbit as in Additional file 1: 1. 5. Complete image series of a precontrast T1w 3D (TFE SENSE) sequence in the same rabbit as in Additional file 1: 1. 6. Complete image series of a postcontrast T1w 3D (TFE SENSE) sequence in the same rabbit as in Additional file 1: 1. [file 13028_2015_139_MOESM1_ESM.zip › Brain_2200809/sT1W_3D_TFE_701/IM-0005-0093.jpg]

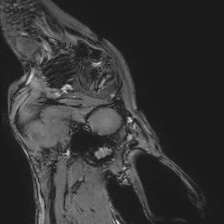

Supplement: Additional file 1: — 1. Complete image series of a transverse TSE T2w sequence in one rabbit. 2. Complete image series of a sagittal TSE T2w sequence in the same rabbit as in Additional file 1: 1. 3. Complete image series of a dorsal TSE T2w sequence in the same rabbit as in Additional file 1: 1. 4. Complete image series of a FLAIR longTR CLEAR sequence in the same rabbit as in Additional file 1: 1. 5. Complete image series of a precontrast T1w 3D (TFE SENSE) sequence in the same rabbit as in Additional file 1: 1. 6. Complete image series of a postcontrast T1w 3D (TFE SENSE) sequence in the same rabbit as in Additional file 1: 1. [file 13028_2015_139_MOESM1_ESM.zip › Brain_2200809/sT1W_3D_TFE_701/IM-0005-0094.jpg]

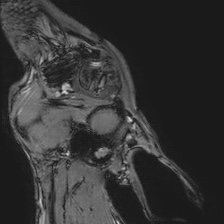

Supplement: Additional file 1: — 1. Complete image series of a transverse TSE T2w sequence in one rabbit. 2. Complete image series of a sagittal TSE T2w sequence in the same rabbit as in Additional file 1: 1. 3. Complete image series of a dorsal TSE T2w sequence in the same rabbit as in Additional file 1: 1. 4. Complete image series of a FLAIR longTR CLEAR sequence in the same rabbit as in Additional file 1: 1. 5. Complete image series of a precontrast T1w 3D (TFE SENSE) sequence in the same rabbit as in Additional file 1: 1. 6. Complete image series of a postcontrast T1w 3D (TFE SENSE) sequence in the same rabbit as in Additional file 1: 1. [file 13028_2015_139_MOESM1_ESM.zip › Brain_2200809/sT1W_3D_TFE_701/IM-0005-0095.jpg]

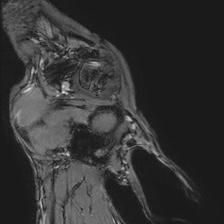

Supplement: Additional file 1: — 1. Complete image series of a transverse TSE T2w sequence in one rabbit. 2. Complete image series of a sagittal TSE T2w sequence in the same rabbit as in Additional file 1: 1. 3. Complete image series of a dorsal TSE T2w sequence in the same rabbit as in Additional file 1: 1. 4. Complete image series of a FLAIR longTR CLEAR sequence in the same rabbit as in Additional file 1: 1. 5. Complete image series of a precontrast T1w 3D (TFE SENSE) sequence in the same rabbit as in Additional file 1: 1. 6. Complete image series of a postcontrast T1w 3D (TFE SENSE) sequence in the same rabbit as in Additional file 1: 1. [file 13028_2015_139_MOESM1_ESM.zip › Brain_2200809/sT1W_3D_TFE_701/IM-0005-0096.jpg]

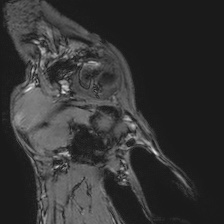

Supplement: Additional file 1: — 1. Complete image series of a transverse TSE T2w sequence in one rabbit. 2. Complete image series of a sagittal TSE T2w sequence in the same rabbit as in Additional file 1: 1. 3. Complete image series of a dorsal TSE T2w sequence in the same rabbit as in Additional file 1: 1. 4. Complete image series of a FLAIR longTR CLEAR sequence in the same rabbit as in Additional file 1: 1. 5. Complete image series of a precontrast T1w 3D (TFE SENSE) sequence in the same rabbit as in Additional file 1: 1. 6. Complete image series of a postcontrast T1w 3D (TFE SENSE) sequence in the same rabbit as in Additional file 1: 1. [file 13028_2015_139_MOESM1_ESM.zip › Brain_2200809/sT1W_3D_TFE_701/IM-0005-0097.jpg]

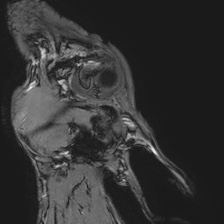

Supplement: Additional file 1: — 1. Complete image series of a transverse TSE T2w sequence in one rabbit. 2. Complete image series of a sagittal TSE T2w sequence in the same rabbit as in Additional file 1: 1. 3. Complete image series of a dorsal TSE T2w sequence in the same rabbit as in Additional file 1: 1. 4. Complete image series of a FLAIR longTR CLEAR sequence in the same rabbit as in Additional file 1: 1. 5. Complete image series of a precontrast T1w 3D (TFE SENSE) sequence in the same rabbit as in Additional file 1: 1. 6. Complete image series of a postcontrast T1w 3D (TFE SENSE) sequence in the same rabbit as in Additional file 1: 1. [file 13028_2015_139_MOESM1_ESM.zip › Brain_2200809/sT1W_3D_TFE_701/IM-0005-0098.jpg]

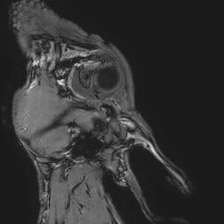

Supplement: Additional file 1: — 1. Complete image series of a transverse TSE T2w sequence in one rabbit. 2. Complete image series of a sagittal TSE T2w sequence in the same rabbit as in Additional file 1: 1. 3. Complete image series of a dorsal TSE T2w sequence in the same rabbit as in Additional file 1: 1. 4. Complete image series of a FLAIR longTR CLEAR sequence in the same rabbit as in Additional file 1: 1. 5. Complete image series of a precontrast T1w 3D (TFE SENSE) sequence in the same rabbit as in Additional file 1: 1. 6. Complete image series of a postcontrast T1w 3D (TFE SENSE) sequence in the same rabbit as in Additional file 1: 1. [file 13028_2015_139_MOESM1_ESM.zip › Brain_2200809/sT1W_3D_TFE_701/IM-0005-0099.jpg]

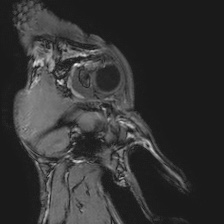

Supplement: Additional file 1: — 1. Complete image series of a transverse TSE T2w sequence in one rabbit. 2. Complete image series of a sagittal TSE T2w sequence in the same rabbit as in Additional file 1: 1. 3. Complete image series of a dorsal TSE T2w sequence in the same rabbit as in Additional file 1: 1. 4. Complete image series of a FLAIR longTR CLEAR sequence in the same rabbit as in Additional file 1: 1. 5. Complete image series of a precontrast T1w 3D (TFE SENSE) sequence in the same rabbit as in Additional file 1: 1. 6. Complete image series of a postcontrast T1w 3D (TFE SENSE) sequence in the same rabbit as in Additional file 1: 1. [file 13028_2015_139_MOESM1_ESM.zip › Brain_2200809/sT1W_3D_TFE_701/IM-0005-0100.jpg]

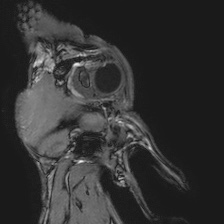

Supplement: Additional file 1: — 1. Complete image series of a transverse TSE T2w sequence in one rabbit. 2. Complete image series of a sagittal TSE T2w sequence in the same rabbit as in Additional file 1: 1. 3. Complete image series of a dorsal TSE T2w sequence in the same rabbit as in Additional file 1: 1. 4. Complete image series of a FLAIR longTR CLEAR sequence in the same rabbit as in Additional file 1: 1. 5. Complete image series of a precontrast T1w 3D (TFE SENSE) sequence in the same rabbit as in Additional file 1: 1. 6. Complete image series of a postcontrast T1w 3D (TFE SENSE) sequence in the same rabbit as in Additional file 1: 1. [file 13028_2015_139_MOESM1_ESM.zip › Brain_2200809/sT1W_3D_TFE_701/IM-0005-0101.jpg]

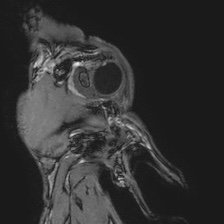

Supplement: Additional file 1: — 1. Complete image series of a transverse TSE T2w sequence in one rabbit. 2. Complete image series of a sagittal TSE T2w sequence in the same rabbit as in Additional file 1: 1. 3. Complete image series of a dorsal TSE T2w sequence in the same rabbit as in Additional file 1: 1. 4. Complete image series of a FLAIR longTR CLEAR sequence in the same rabbit as in Additional file 1: 1. 5. Complete image series of a precontrast T1w 3D (TFE SENSE) sequence in the same rabbit as in Additional file 1: 1. 6. Complete image series of a postcontrast T1w 3D (TFE SENSE) sequence in the same rabbit as in Additional file 1: 1. [file 13028_2015_139_MOESM1_ESM.zip › Brain_2200809/sT1W_3D_TFE_701/IM-0005-0102.jpg]

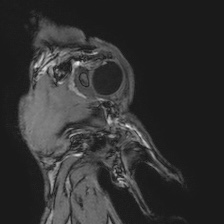

Supplement: Additional file 1: — 1. Complete image series of a transverse TSE T2w sequence in one rabbit. 2. Complete image series of a sagittal TSE T2w sequence in the same rabbit as in Additional file 1: 1. 3. Complete image series of a dorsal TSE T2w sequence in the same rabbit as in Additional file 1: 1. 4. Complete image series of a FLAIR longTR CLEAR sequence in the same rabbit as in Additional file 1: 1. 5. Complete image series of a precontrast T1w 3D (TFE SENSE) sequence in the same rabbit as in Additional file 1: 1. 6. Complete image series of a postcontrast T1w 3D (TFE SENSE) sequence in the same rabbit as in Additional file 1: 1. [file 13028_2015_139_MOESM1_ESM.zip › Brain_2200809/sT1W_3D_TFE_701/IM-0005-0103.jpg]

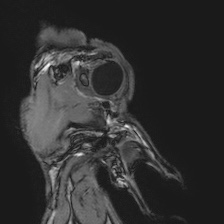

Supplement: Additional file 1: — 1. Complete image series of a transverse TSE T2w sequence in one rabbit. 2. Complete image series of a sagittal TSE T2w sequence in the same rabbit as in Additional file 1: 1. 3. Complete image series of a dorsal TSE T2w sequence in the same rabbit as in Additional file 1: 1. 4. Complete image series of a FLAIR longTR CLEAR sequence in the same rabbit as in Additional file 1: 1. 5. Complete image series of a precontrast T1w 3D (TFE SENSE) sequence in the same rabbit as in Additional file 1: 1. 6. Complete image series of a postcontrast T1w 3D (TFE SENSE) sequence in the same rabbit as in Additional file 1: 1. [file 13028_2015_139_MOESM1_ESM.zip › Brain_2200809/sT1W_3D_TFE_701/IM-0005-0104.jpg]

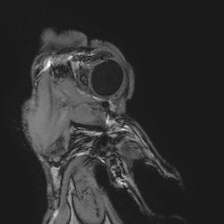

Supplement: Additional file 1: — 1. Complete image series of a transverse TSE T2w sequence in one rabbit. 2. Complete image series of a sagittal TSE T2w sequence in the same rabbit as in Additional file 1: 1. 3. Complete image series of a dorsal TSE T2w sequence in the same rabbit as in Additional file 1: 1. 4. Complete image series of a FLAIR longTR CLEAR sequence in the same rabbit as in Additional file 1: 1. 5. Complete image series of a precontrast T1w 3D (TFE SENSE) sequence in the same rabbit as in Additional file 1: 1. 6. Complete image series of a postcontrast T1w 3D (TFE SENSE) sequence in the same rabbit as in Additional file 1: 1. [file 13028_2015_139_MOESM1_ESM.zip › Brain_2200809/sT1W_3D_TFE_701/IM-0005-0105.jpg]

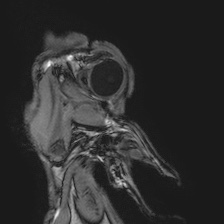

Supplement: Additional file 1: — 1. Complete image series of a transverse TSE T2w sequence in one rabbit. 2. Complete image series of a sagittal TSE T2w sequence in the same rabbit as in Additional file 1: 1. 3. Complete image series of a dorsal TSE T2w sequence in the same rabbit as in Additional file 1: 1. 4. Complete image series of a FLAIR longTR CLEAR sequence in the same rabbit as in Additional file 1: 1. 5. Complete image series of a precontrast T1w 3D (TFE SENSE) sequence in the same rabbit as in Additional file 1: 1. 6. Complete image series of a postcontrast T1w 3D (TFE SENSE) sequence in the same rabbit as in Additional file 1: 1. [file 13028_2015_139_MOESM1_ESM.zip › Brain_2200809/sT1W_3D_TFE_701/IM-0005-0106.jpg]

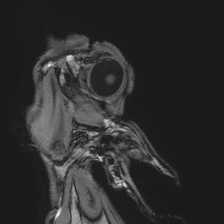

Supplement: Additional file 1: — 1. Complete image series of a transverse TSE T2w sequence in one rabbit. 2. Complete image series of a sagittal TSE T2w sequence in the same rabbit as in Additional file 1: 1. 3. Complete image series of a dorsal TSE T2w sequence in the same rabbit as in Additional file 1: 1. 4. Complete image series of a FLAIR longTR CLEAR sequence in the same rabbit as in Additional file 1: 1. 5. Complete image series of a precontrast T1w 3D (TFE SENSE) sequence in the same rabbit as in Additional file 1: 1. 6. Complete image series of a postcontrast T1w 3D (TFE SENSE) sequence in the same rabbit as in Additional file 1: 1. [file 13028_2015_139_MOESM1_ESM.zip › Brain_2200809/sT1W_3D_TFE_701/IM-0005-0107.jpg]

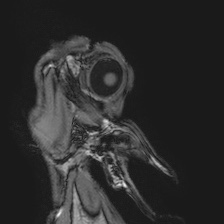

Supplement: Additional file 1: — 1. Complete image series of a transverse TSE T2w sequence in one rabbit. 2. Complete image series of a sagittal TSE T2w sequence in the same rabbit as in Additional file 1: 1. 3. Complete image series of a dorsal TSE T2w sequence in the same rabbit as in Additional file 1: 1. 4. Complete image series of a FLAIR longTR CLEAR sequence in the same rabbit as in Additional file 1: 1. 5. Complete image series of a precontrast T1w 3D (TFE SENSE) sequence in the same rabbit as in Additional file 1: 1. 6. Complete image series of a postcontrast T1w 3D (TFE SENSE) sequence in the same rabbit as in Additional file 1: 1. [file 13028_2015_139_MOESM1_ESM.zip › Brain_2200809/sT1W_3D_TFE_701/IM-0005-0108.jpg]

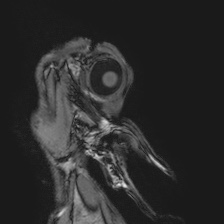

Supplement: Additional file 1: — 1. Complete image series of a transverse TSE T2w sequence in one rabbit. 2. Complete image series of a sagittal TSE T2w sequence in the same rabbit as in Additional file 1: 1. 3. Complete image series of a dorsal TSE T2w sequence in the same rabbit as in Additional file 1: 1. 4. Complete image series of a FLAIR longTR CLEAR sequence in the same rabbit as in Additional file 1: 1. 5. Complete image series of a precontrast T1w 3D (TFE SENSE) sequence in the same rabbit as in Additional file 1: 1. 6. Complete image series of a postcontrast T1w 3D (TFE SENSE) sequence in the same rabbit as in Additional file 1: 1. [file 13028_2015_139_MOESM1_ESM.zip › Brain_2200809/sT1W_3D_TFE_701/IM-0005-0109.jpg]

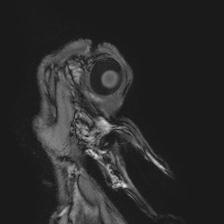

Supplement: Additional file 1: — 1. Complete image series of a transverse TSE T2w sequence in one rabbit. 2. Complete image series of a sagittal TSE T2w sequence in the same rabbit as in Additional file 1: 1. 3. Complete image series of a dorsal TSE T2w sequence in the same rabbit as in Additional file 1: 1. 4. Complete image series of a FLAIR longTR CLEAR sequence in the same rabbit as in Additional file 1: 1. 5. Complete image series of a precontrast T1w 3D (TFE SENSE) sequence in the same rabbit as in Additional file 1: 1. 6. Complete image series of a postcontrast T1w 3D (TFE SENSE) sequence in the same rabbit as in Additional file 1: 1. [file 13028_2015_139_MOESM1_ESM.zip › Brain_2200809/sT1W_3D_TFE_701/IM-0005-0110.jpg]

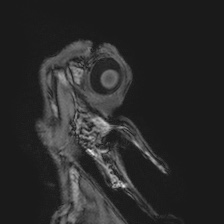

Supplement: Additional file 1: — 1. Complete image series of a transverse TSE T2w sequence in one rabbit. 2. Complete image series of a sagittal TSE T2w sequence in the same rabbit as in Additional file 1: 1. 3. Complete image series of a dorsal TSE T2w sequence in the same rabbit as in Additional file 1: 1. 4. Complete image series of a FLAIR longTR CLEAR sequence in the same rabbit as in Additional file 1: 1. 5. Complete image series of a precontrast T1w 3D (TFE SENSE) sequence in the same rabbit as in Additional file 1: 1. 6. Complete image series of a postcontrast T1w 3D (TFE SENSE) sequence in the same rabbit as in Additional file 1: 1. [file 13028_2015_139_MOESM1_ESM.zip › Brain_2200809/sT1W_3D_TFE_701/IM-0005-0111.jpg]

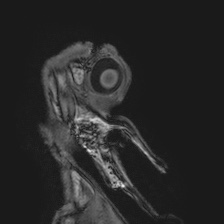

Supplement: Additional file 1: — 1. Complete image series of a transverse TSE T2w sequence in one rabbit. 2. Complete image series of a sagittal TSE T2w sequence in the same rabbit as in Additional file 1: 1. 3. Complete image series of a dorsal TSE T2w sequence in the same rabbit as in Additional file 1: 1. 4. Complete image series of a FLAIR longTR CLEAR sequence in the same rabbit as in Additional file 1: 1. 5. Complete image series of a precontrast T1w 3D (TFE SENSE) sequence in the same rabbit as in Additional file 1: 1. 6. Complete image series of a postcontrast T1w 3D (TFE SENSE) sequence in the same rabbit as in Additional file 1: 1. [file 13028_2015_139_MOESM1_ESM.zip › Brain_2200809/sT1W_3D_TFE_701/IM-0005-0112.jpg]

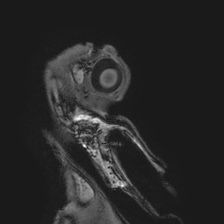

Supplement: Additional file 1: — 1. Complete image series of a transverse TSE T2w sequence in one rabbit. 2. Complete image series of a sagittal TSE T2w sequence in the same rabbit as in Additional file 1: 1. 3. Complete image series of a dorsal TSE T2w sequence in the same rabbit as in Additional file 1: 1. 4. Complete image series of a FLAIR longTR CLEAR sequence in the same rabbit as in Additional file 1: 1. 5. Complete image series of a precontrast T1w 3D (TFE SENSE) sequence in the same rabbit as in Additional file 1: 1. 6. Complete image series of a postcontrast T1w 3D (TFE SENSE) sequence in the same rabbit as in Additional file 1: 1. [file 13028_2015_139_MOESM1_ESM.zip › Brain_2200809/sT1W_3D_TFE_701/IM-0005-0113.jpg]

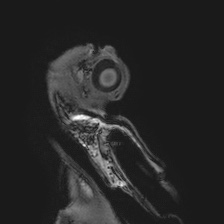

Supplement: Additional file 1: — 1. Complete image series of a transverse TSE T2w sequence in one rabbit. 2. Complete image series of a sagittal TSE T2w sequence in the same rabbit as in Additional file 1: 1. 3. Complete image series of a dorsal TSE T2w sequence in the same rabbit as in Additional file 1: 1. 4. Complete image series of a FLAIR longTR CLEAR sequence in the same rabbit as in Additional file 1: 1. 5. Complete image series of a precontrast T1w 3D (TFE SENSE) sequence in the same rabbit as in Additional file 1: 1. 6. Complete image series of a postcontrast T1w 3D (TFE SENSE) sequence in the same rabbit as in Additional file 1: 1. [file 13028_2015_139_MOESM1_ESM.zip › Brain_2200809/sT1W_3D_TFE_701/IM-0005-0114.jpg]

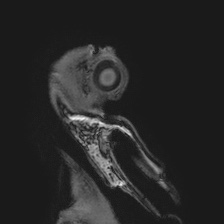

Supplement: Additional file 1: — 1. Complete image series of a transverse TSE T2w sequence in one rabbit. 2. Complete image series of a sagittal TSE T2w sequence in the same rabbit as in Additional file 1: 1. 3. Complete image series of a dorsal TSE T2w sequence in the same rabbit as in Additional file 1: 1. 4. Complete image series of a FLAIR longTR CLEAR sequence in the same rabbit as in Additional file 1: 1. 5. Complete image series of a precontrast T1w 3D (TFE SENSE) sequence in the same rabbit as in Additional file 1: 1. 6. Complete image series of a postcontrast T1w 3D (TFE SENSE) sequence in the same rabbit as in Additional file 1: 1. [file 13028_2015_139_MOESM1_ESM.zip › Brain_2200809/sT1W_3D_TFE_701/IM-0005-0115.jpg]

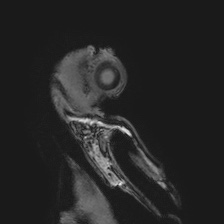

Supplement: Additional file 1: — 1. Complete image series of a transverse TSE T2w sequence in one rabbit. 2. Complete image series of a sagittal TSE T2w sequence in the same rabbit as in Additional file 1: 1. 3. Complete image series of a dorsal TSE T2w sequence in the same rabbit as in Additional file 1: 1. 4. Complete image series of a FLAIR longTR CLEAR sequence in the same rabbit as in Additional file 1: 1. 5. Complete image series of a precontrast T1w 3D (TFE SENSE) sequence in the same rabbit as in Additional file 1: 1. 6. Complete image series of a postcontrast T1w 3D (TFE SENSE) sequence in the same rabbit as in Additional file 1: 1. [file 13028_2015_139_MOESM1_ESM.zip › Brain_2200809/sT1W_3D_TFE_701/IM-0005-0116.jpg]

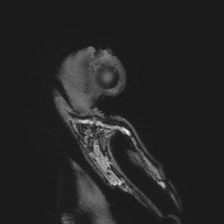

Supplement: Additional file 1: — 1. Complete image series of a transverse TSE T2w sequence in one rabbit. 2. Complete image series of a sagittal TSE T2w sequence in the same rabbit as in Additional file 1: 1. 3. Complete image series of a dorsal TSE T2w sequence in the same rabbit as in Additional file 1: 1. 4. Complete image series of a FLAIR longTR CLEAR sequence in the same rabbit as in Additional file 1: 1. 5. Complete image series of a precontrast T1w 3D (TFE SENSE) sequence in the same rabbit as in Additional file 1: 1. 6. Complete image series of a postcontrast T1w 3D (TFE SENSE) sequence in the same rabbit as in Additional file 1: 1. [file 13028_2015_139_MOESM1_ESM.zip › Brain_2200809/sT1W_3D_TFE_701/IM-0005-0117.jpg]

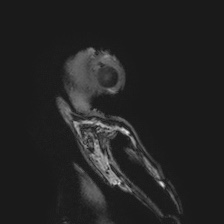

Supplement: Additional file 1: — 1. Complete image series of a transverse TSE T2w sequence in one rabbit. 2. Complete image series of a sagittal TSE T2w sequence in the same rabbit as in Additional file 1: 1. 3. Complete image series of a dorsal TSE T2w sequence in the same rabbit as in Additional file 1: 1. 4. Complete image series of a FLAIR longTR CLEAR sequence in the same rabbit as in Additional file 1: 1. 5. Complete image series of a precontrast T1w 3D (TFE SENSE) sequence in the same rabbit as in Additional file 1: 1. 6. Complete image series of a postcontrast T1w 3D (TFE SENSE) sequence in the same rabbit as in Additional file 1: 1. [file 13028_2015_139_MOESM1_ESM.zip › Brain_2200809/sT1W_3D_TFE_701/IM-0005-0118.jpg]

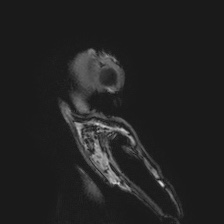

Supplement: Additional file 1: — 1. Complete image series of a transverse TSE T2w sequence in one rabbit. 2. Complete image series of a sagittal TSE T2w sequence in the same rabbit as in Additional file 1: 1. 3. Complete image series of a dorsal TSE T2w sequence in the same rabbit as in Additional file 1: 1. 4. Complete image series of a FLAIR longTR CLEAR sequence in the same rabbit as in Additional file 1: 1. 5. Complete image series of a precontrast T1w 3D (TFE SENSE) sequence in the same rabbit as in Additional file 1: 1. 6. Complete image series of a postcontrast T1w 3D (TFE SENSE) sequence in the same rabbit as in Additional file 1: 1. [file 13028_2015_139_MOESM1_ESM.zip › Brain_2200809/sT1W_3D_TFE_701/IM-0005-0119.jpg]

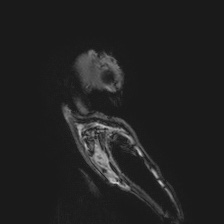

Supplement: Additional file 1: — 1. Complete image series of a transverse TSE T2w sequence in one rabbit. 2. Complete image series of a sagittal TSE T2w sequence in the same rabbit as in Additional file 1: 1. 3. Complete image series of a dorsal TSE T2w sequence in the same rabbit as in Additional file 1: 1. 4. Complete image series of a FLAIR longTR CLEAR sequence in the same rabbit as in Additional file 1: 1. 5. Complete image series of a precontrast T1w 3D (TFE SENSE) sequence in the same rabbit as in Additional file 1: 1. 6. Complete image series of a postcontrast T1w 3D (TFE SENSE) sequence in the same rabbit as in Additional file 1: 1. [file 13028_2015_139_MOESM1_ESM.zip › Brain_2200809/sT1W_3D_TFE_701/IM-0005-0120.jpg]

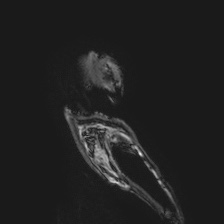

Supplement: Additional file 1: — 1. Complete image series of a transverse TSE T2w sequence in one rabbit. 2. Complete image series of a sagittal TSE T2w sequence in the same rabbit as in Additional file 1: 1. 3. Complete image series of a dorsal TSE T2w sequence in the same rabbit as in Additional file 1: 1. 4. Complete image series of a FLAIR longTR CLEAR sequence in the same rabbit as in Additional file 1: 1. 5. Complete image series of a precontrast T1w 3D (TFE SENSE) sequence in the same rabbit as in Additional file 1: 1. 6. Complete image series of a postcontrast T1w 3D (TFE SENSE) sequence in the same rabbit as in Additional file 1: 1. [file 13028_2015_139_MOESM1_ESM.zip › Brain_2200809/sT1W_3D_TFE_701/IM-0005-0121.jpg]

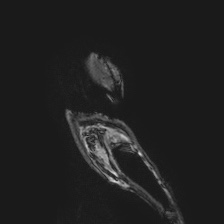

Supplement: Additional file 1: — 1. Complete image series of a transverse TSE T2w sequence in one rabbit. 2. Complete image series of a sagittal TSE T2w sequence in the same rabbit as in Additional file 1: 1. 3. Complete image series of a dorsal TSE T2w sequence in the same rabbit as in Additional file 1: 1. 4. Complete image series of a FLAIR longTR CLEAR sequence in the same rabbit as in Additional file 1: 1. 5. Complete image series of a precontrast T1w 3D (TFE SENSE) sequence in the same rabbit as in Additional file 1: 1. 6. Complete image series of a postcontrast T1w 3D (TFE SENSE) sequence in the same rabbit as in Additional file 1: 1. [file 13028_2015_139_MOESM1_ESM.zip › Brain_2200809/sT1W_3D_TFE_701/IM-0005-0122.jpg]

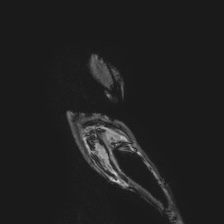

Supplement: Additional file 1: — 1. Complete image series of a transverse TSE T2w sequence in one rabbit. 2. Complete image series of a sagittal TSE T2w sequence in the same rabbit as in Additional file 1: 1. 3. Complete image series of a dorsal TSE T2w sequence in the same rabbit as in Additional file 1: 1. 4. Complete image series of a FLAIR longTR CLEAR sequence in the same rabbit as in Additional file 1: 1. 5. Complete image series of a precontrast T1w 3D (TFE SENSE) sequence in the same rabbit as in Additional file 1: 1. 6. Complete image series of a postcontrast T1w 3D (TFE SENSE) sequence in the same rabbit as in Additional file 1: 1. [file 13028_2015_139_MOESM1_ESM.zip › Brain_2200809/sT1W_3D_TFE_701/IM-0005-0123.jpg]

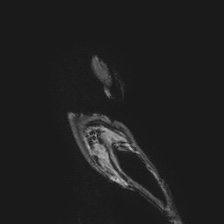

Supplement: Additional file 1: — 1. Complete image series of a transverse TSE T2w sequence in one rabbit. 2. Complete image series of a sagittal TSE T2w sequence in the same rabbit as in Additional file 1: 1. 3. Complete image series of a dorsal TSE T2w sequence in the same rabbit as in Additional file 1: 1. 4. Complete image series of a FLAIR longTR CLEAR sequence in the same rabbit as in Additional file 1: 1. 5. Complete image series of a precontrast T1w 3D (TFE SENSE) sequence in the same rabbit as in Additional file 1: 1. 6. Complete image series of a postcontrast T1w 3D (TFE SENSE) sequence in the same rabbit as in Additional file 1: 1. [file 13028_2015_139_MOESM1_ESM.zip › Brain_2200809/sT1W_3D_TFE_701/IM-0005-0124.jpg]

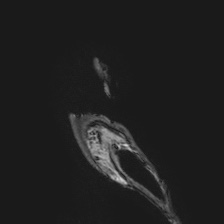

Supplement: Additional file 1: — 1. Complete image series of a transverse TSE T2w sequence in one rabbit. 2. Complete image series of a sagittal TSE T2w sequence in the same rabbit as in Additional file 1: 1. 3. Complete image series of a dorsal TSE T2w sequence in the same rabbit as in Additional file 1: 1. 4. Complete image series of a FLAIR longTR CLEAR sequence in the same rabbit as in Additional file 1: 1. 5. Complete image series of a precontrast T1w 3D (TFE SENSE) sequence in the same rabbit as in Additional file 1: 1. 6. Complete image series of a postcontrast T1w 3D (TFE SENSE) sequence in the same rabbit as in Additional file 1: 1. [file 13028_2015_139_MOESM1_ESM.zip › Brain_2200809/sT1W_3D_TFE_701/IM-0005-0125.jpg]

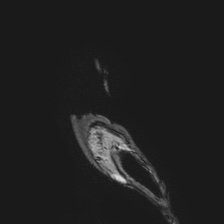

Supplement: Additional file 1: — 1. Complete image series of a transverse TSE T2w sequence in one rabbit. 2. Complete image series of a sagittal TSE T2w sequence in the same rabbit as in Additional file 1: 1. 3. Complete image series of a dorsal TSE T2w sequence in the same rabbit as in Additional file 1: 1. 4. Complete image series of a FLAIR longTR CLEAR sequence in the same rabbit as in Additional file 1: 1. 5. Complete image series of a precontrast T1w 3D (TFE SENSE) sequence in the same rabbit as in Additional file 1: 1. 6. Complete image series of a postcontrast T1w 3D (TFE SENSE) sequence in the same rabbit as in Additional file 1: 1. [file 13028_2015_139_MOESM1_ESM.zip › Brain_2200809/sT1W_3D_TFE_701/IM-0005-0126.jpg]

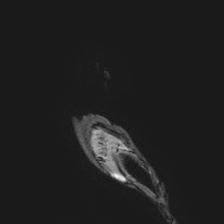

Supplement: Additional file 1: — 1. Complete image series of a transverse TSE T2w sequence in one rabbit. 2. Complete image series of a sagittal TSE T2w sequence in the same rabbit as in Additional file 1: 1. 3. Complete image series of a dorsal TSE T2w sequence in the same rabbit as in Additional file 1: 1. 4. Complete image series of a FLAIR longTR CLEAR sequence in the same rabbit as in Additional file 1: 1. 5. Complete image series of a precontrast T1w 3D (TFE SENSE) sequence in the same rabbit as in Additional file 1: 1. 6. Complete image series of a postcontrast T1w 3D (TFE SENSE) sequence in the same rabbit as in Additional file 1: 1. [file 13028_2015_139_MOESM1_ESM.zip › Brain_2200809/sT1W_3D_TFE_701/IM-0005-0127.jpg]

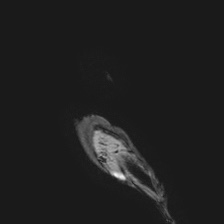

Supplement: Additional file 1: — 1. Complete image series of a transverse TSE T2w sequence in one rabbit. 2. Complete image series of a sagittal TSE T2w sequence in the same rabbit as in Additional file 1: 1. 3. Complete image series of a dorsal TSE T2w sequence in the same rabbit as in Additional file 1: 1. 4. Complete image series of a FLAIR longTR CLEAR sequence in the same rabbit as in Additional file 1: 1. 5. Complete image series of a precontrast T1w 3D (TFE SENSE) sequence in the same rabbit as in Additional file 1: 1. 6. Complete image series of a postcontrast T1w 3D (TFE SENSE) sequence in the same rabbit as in Additional file 1: 1. [file 13028_2015_139_MOESM1_ESM.zip › Brain_2200809/sT1W_3D_TFE_701/IM-0005-0128.jpg]

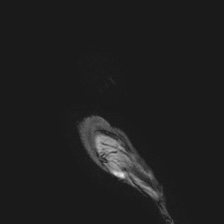

Supplement: Additional file 1: — 1. Complete image series of a transverse TSE T2w sequence in one rabbit. 2. Complete image series of a sagittal TSE T2w sequence in the same rabbit as in Additional file 1: 1. 3. Complete image series of a dorsal TSE T2w sequence in the same rabbit as in Additional file 1: 1. 4. Complete image series of a FLAIR longTR CLEAR sequence in the same rabbit as in Additional file 1: 1. 5. Complete image series of a precontrast T1w 3D (TFE SENSE) sequence in the same rabbit as in Additional file 1: 1. 6. Complete image series of a postcontrast T1w 3D (TFE SENSE) sequence in the same rabbit as in Additional file 1: 1. [file 13028_2015_139_MOESM1_ESM.zip › Brain_2200809/sT1W_3D_TFE_701/IM-0005-0129.jpg]

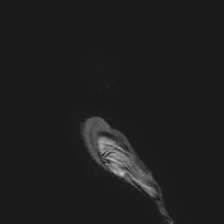

Supplement: Additional file 1: — 1. Complete image series of a transverse TSE T2w sequence in one rabbit. 2. Complete image series of a sagittal TSE T2w sequence in the same rabbit as in Additional file 1: 1. 3. Complete image series of a dorsal TSE T2w sequence in the same rabbit as in Additional file 1: 1. 4. Complete image series of a FLAIR longTR CLEAR sequence in the same rabbit as in Additional file 1: 1. 5. Complete image series of a precontrast T1w 3D (TFE SENSE) sequence in the same rabbit as in Additional file 1: 1. 6. Complete image series of a postcontrast T1w 3D (TFE SENSE) sequence in the same rabbit as in Additional file 1: 1. [file 13028_2015_139_MOESM1_ESM.zip › Brain_2200809/sT1W_3D_TFE_701/IM-0005-0130.jpg]

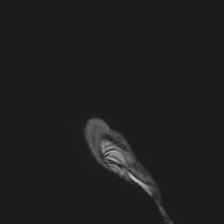

Supplement: Additional file 1: — 1. Complete image series of a transverse TSE T2w sequence in one rabbit. 2. Complete image series of a sagittal TSE T2w sequence in the same rabbit as in Additional file 1: 1. 3. Complete image series of a dorsal TSE T2w sequence in the same rabbit as in Additional file 1: 1. 4. Complete image series of a FLAIR longTR CLEAR sequence in the same rabbit as in Additional file 1: 1. 5. Complete image series of a precontrast T1w 3D (TFE SENSE) sequence in the same rabbit as in Additional file 1: 1. 6. Complete image series of a postcontrast T1w 3D (TFE SENSE) sequence in the same rabbit as in Additional file 1: 1. [file 13028_2015_139_MOESM1_ESM.zip › Brain_2200809/sT1W_3D_TFE_701/IM-0005-0131.jpg]

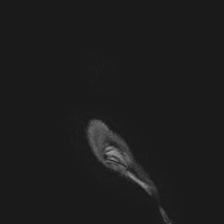

Supplement: Additional file 1: — 1. Complete image series of a transverse TSE T2w sequence in one rabbit. 2. Complete image series of a sagittal TSE T2w sequence in the same rabbit as in Additional file 1: 1. 3. Complete image series of a dorsal TSE T2w sequence in the same rabbit as in Additional file 1: 1. 4. Complete image series of a FLAIR longTR CLEAR sequence in the same rabbit as in Additional file 1: 1. 5. Complete image series of a precontrast T1w 3D (TFE SENSE) sequence in the same rabbit as in Additional file 1: 1. 6. Complete image series of a postcontrast T1w 3D (TFE SENSE) sequence in the same rabbit as in Additional file 1: 1. [file 13028_2015_139_MOESM1_ESM.zip › Brain_2200809/sT1W_3D_TFE_701/IM-0005-0132.jpg]

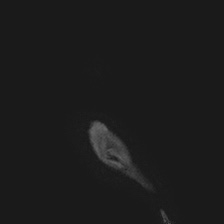

Supplement: Additional file 1: — 1. Complete image series of a transverse TSE T2w sequence in one rabbit. 2. Complete image series of a sagittal TSE T2w sequence in the same rabbit as in Additional file 1: 1. 3. Complete image series of a dorsal TSE T2w sequence in the same rabbit as in Additional file 1: 1. 4. Complete image series of a FLAIR longTR CLEAR sequence in the same rabbit as in Additional file 1: 1. 5. Complete image series of a precontrast T1w 3D (TFE SENSE) sequence in the same rabbit as in Additional file 1: 1. 6. Complete image series of a postcontrast T1w 3D (TFE SENSE) sequence in the same rabbit as in Additional file 1: 1. [file 13028_2015_139_MOESM1_ESM.zip › Brain_2200809/sT1W_3D_TFE_701/IM-0005-0133.jpg]

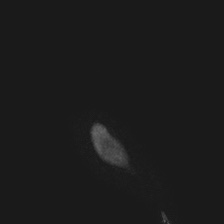

Supplement: Additional file 1: — 1. Complete image series of a transverse TSE T2w sequence in one rabbit. 2. Complete image series of a sagittal TSE T2w sequence in the same rabbit as in Additional file 1: 1. 3. Complete image series of a dorsal TSE T2w sequence in the same rabbit as in Additional file 1: 1. 4. Complete image series of a FLAIR longTR CLEAR sequence in the same rabbit as in Additional file 1: 1. 5. Complete image series of a precontrast T1w 3D (TFE SENSE) sequence in the same rabbit as in Additional file 1: 1. 6. Complete image series of a postcontrast T1w 3D (TFE SENSE) sequence in the same rabbit as in Additional file 1: 1. [file 13028_2015_139_MOESM1_ESM.zip › Brain_2200809/sT1W_3D_TFE_701/IM-0005-0134.jpg]

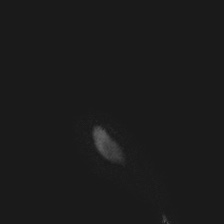

Supplement: Additional file 1: — 1. Complete image series of a transverse TSE T2w sequence in one rabbit. 2. Complete image series of a sagittal TSE T2w sequence in the same rabbit as in Additional file 1: 1. 3. Complete image series of a dorsal TSE T2w sequence in the same rabbit as in Additional file 1: 1. 4. Complete image series of a FLAIR longTR CLEAR sequence in the same rabbit as in Additional file 1: 1. 5. Complete image series of a precontrast T1w 3D (TFE SENSE) sequence in the same rabbit as in Additional file 1: 1. 6. Complete image series of a postcontrast T1w 3D (TFE SENSE) sequence in the same rabbit as in Additional file 1: 1. [file 13028_2015_139_MOESM1_ESM.zip › Brain_2200809/sT1W_3D_TFE_701/IM-0005-0135.jpg]

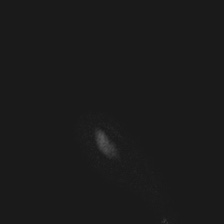

Supplement: Additional file 1: — 1. Complete image series of a transverse TSE T2w sequence in one rabbit. 2. Complete image series of a sagittal TSE T2w sequence in the same rabbit as in Additional file 1: 1. 3. Complete image series of a dorsal TSE T2w sequence in the same rabbit as in Additional file 1: 1. 4. Complete image series of a FLAIR longTR CLEAR sequence in the same rabbit as in Additional file 1: 1. 5. Complete image series of a precontrast T1w 3D (TFE SENSE) sequence in the same rabbit as in Additional file 1: 1. 6. Complete image series of a postcontrast T1w 3D (TFE SENSE) sequence in the same rabbit as in Additional file 1: 1. [file 13028_2015_139_MOESM1_ESM.zip › Brain_2200809/sT1W_3D_TFE_701/IM-0005-0136.jpg]

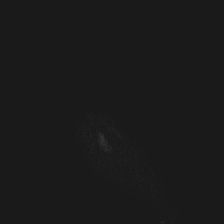

Supplement: Additional file 1: — 1. Complete image series of a transverse TSE T2w sequence in one rabbit. 2. Complete image series of a sagittal TSE T2w sequence in the same rabbit as in Additional file 1: 1. 3. Complete image series of a dorsal TSE T2w sequence in the same rabbit as in Additional file 1: 1. 4. Complete image series of a FLAIR longTR CLEAR sequence in the same rabbit as in Additional file 1: 1. 5. Complete image series of a precontrast T1w 3D (TFE SENSE) sequence in the same rabbit as in Additional file 1: 1. 6. Complete image series of a postcontrast T1w 3D (TFE SENSE) sequence in the same rabbit as in Additional file 1: 1. [file 13028_2015_139_MOESM1_ESM.zip › Brain_2200809/sT1W_3D_TFE_701/IM-0005-0137.jpg]

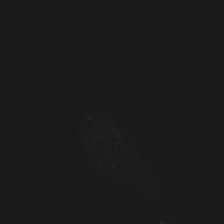

Supplement: Additional file 1: — 1. Complete image series of a transverse TSE T2w sequence in one rabbit. 2. Complete image series of a sagittal TSE T2w sequence in the same rabbit as in Additional file 1: 1. 3. Complete image series of a dorsal TSE T2w sequence in the same rabbit as in Additional file 1: 1. 4. Complete image series of a FLAIR longTR CLEAR sequence in the same rabbit as in Additional file 1: 1. 5. Complete image series of a precontrast T1w 3D (TFE SENSE) sequence in the same rabbit as in Additional file 1: 1. 6. Complete image series of a postcontrast T1w 3D (TFE SENSE) sequence in the same rabbit as in Additional file 1: 1. [file 13028_2015_139_MOESM1_ESM.zip › Brain_2200809/sT1W_3D_TFE_701/IM-0005-0138.jpg]

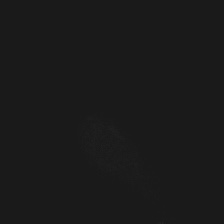

Supplement: Additional file 1: — 1. Complete image series of a transverse TSE T2w sequence in one rabbit. 2. Complete image series of a sagittal TSE T2w sequence in the same rabbit as in Additional file 1: 1. 3. Complete image series of a dorsal TSE T2w sequence in the same rabbit as in Additional file 1: 1. 4. Complete image series of a FLAIR longTR CLEAR sequence in the same rabbit as in Additional file 1: 1. 5. Complete image series of a precontrast T1w 3D (TFE SENSE) sequence in the same rabbit as in Additional file 1: 1. 6. Complete image series of a postcontrast T1w 3D (TFE SENSE) sequence in the same rabbit as in Additional file 1: 1. [file 13028_2015_139_MOESM1_ESM.zip › Brain_2200809/sT1W_3D_TFE_701/IM-0005-0139.jpg]

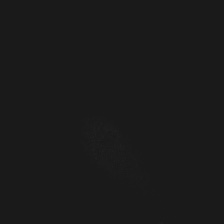

Supplement: Additional file 1: — 1. Complete image series of a transverse TSE T2w sequence in one rabbit. 2. Complete image series of a sagittal TSE T2w sequence in the same rabbit as in Additional file 1: 1. 3. Complete image series of a dorsal TSE T2w sequence in the same rabbit as in Additional file 1: 1. 4. Complete image series of a FLAIR longTR CLEAR sequence in the same rabbit as in Additional file 1: 1. 5. Complete image series of a precontrast T1w 3D (TFE SENSE) sequence in the same rabbit as in Additional file 1: 1. 6. Complete image series of a postcontrast T1w 3D (TFE SENSE) sequence in the same rabbit as in Additional file 1: 1. [file 13028_2015_139_MOESM1_ESM.zip › Brain_2200809/sT1W_3D_TFE_701/IM-0005-0140.jpg]

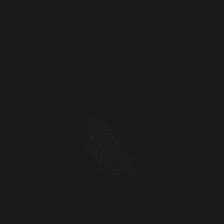

Supplement: Additional file 1: — 1. Complete image series of a transverse TSE T2w sequence in one rabbit. 2. Complete image series of a sagittal TSE T2w sequence in the same rabbit as in Additional file 1: 1. 3. Complete image series of a dorsal TSE T2w sequence in the same rabbit as in Additional file 1: 1. 4. Complete image series of a FLAIR longTR CLEAR sequence in the same rabbit as in Additional file 1: 1. 5. Complete image series of a precontrast T1w 3D (TFE SENSE) sequence in the same rabbit as in Additional file 1: 1. 6. Complete image series of a postcontrast T1w 3D (TFE SENSE) sequence in the same rabbit as in Additional file 1: 1. [file 13028_2015_139_MOESM1_ESM.zip › Brain_2200809/sT1W_3D_TFE_701/IM-0005-0141.jpg]

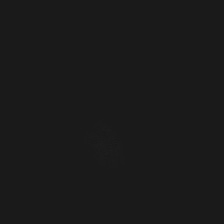

Supplement: Additional file 1: — 1. Complete image series of a transverse TSE T2w sequence in one rabbit. 2. Complete image series of a sagittal TSE T2w sequence in the same rabbit as in Additional file 1: 1. 3. Complete image series of a dorsal TSE T2w sequence in the same rabbit as in Additional file 1: 1. 4. Complete image series of a FLAIR longTR CLEAR sequence in the same rabbit as in Additional file 1: 1. 5. Complete image series of a precontrast T1w 3D (TFE SENSE) sequence in the same rabbit as in Additional file 1: 1. 6. Complete image series of a postcontrast T1w 3D (TFE SENSE) sequence in the same rabbit as in Additional file 1: 1. [file 13028_2015_139_MOESM1_ESM.zip › Brain_2200809/sT1W_3D_TFE_701/IM-0005-0142.jpg]

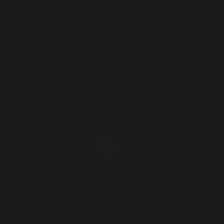

Supplement: Additional file 1: — 1. Complete image series of a transverse TSE T2w sequence in one rabbit. 2. Complete image series of a sagittal TSE T2w sequence in the same rabbit as in Additional file 1: 1. 3. Complete image series of a dorsal TSE T2w sequence in the same rabbit as in Additional file 1: 1. 4. Complete image series of a FLAIR longTR CLEAR sequence in the same rabbit as in Additional file 1: 1. 5. Complete image series of a precontrast T1w 3D (TFE SENSE) sequence in the same rabbit as in Additional file 1: 1. 6. Complete image series of a postcontrast T1w 3D (TFE SENSE) sequence in the same rabbit as in Additional file 1: 1. [file 13028_2015_139_MOESM1_ESM.zip › Brain_2200809/sT1W_3D_TFE_701/IM-0005-0143.jpg]

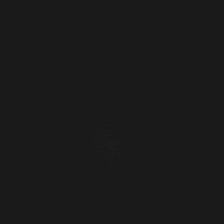

Supplement: Additional file 1: — 1. Complete image series of a transverse TSE T2w sequence in one rabbit. 2. Complete image series of a sagittal TSE T2w sequence in the same rabbit as in Additional file 1: 1. 3. Complete image series of a dorsal TSE T2w sequence in the same rabbit as in Additional file 1: 1. 4. Complete image series of a FLAIR longTR CLEAR sequence in the same rabbit as in Additional file 1: 1. 5. Complete image series of a precontrast T1w 3D (TFE SENSE) sequence in the same rabbit as in Additional file 1: 1. 6. Complete image series of a postcontrast T1w 3D (TFE SENSE) sequence in the same rabbit as in Additional file 1: 1. [file 13028_2015_139_MOESM1_ESM.zip › Brain_2200809/sT1W_3D_TFE_701/IM-0005-0144.jpg]

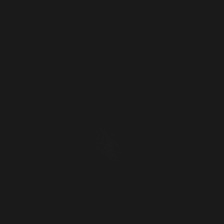

Supplement: Additional file 1: — 1. Complete image series of a transverse TSE T2w sequence in one rabbit. 2. Complete image series of a sagittal TSE T2w sequence in the same rabbit as in Additional file 1: 1. 3. Complete image series of a dorsal TSE T2w sequence in the same rabbit as in Additional file 1: 1. 4. Complete image series of a FLAIR longTR CLEAR sequence in the same rabbit as in Additional file 1: 1. 5. Complete image series of a precontrast T1w 3D (TFE SENSE) sequence in the same rabbit as in Additional file 1: 1. 6. Complete image series of a postcontrast T1w 3D (TFE SENSE) sequence in the same rabbit as in Additional file 1: 1. [file 13028_2015_139_MOESM1_ESM.zip › Brain_2200809/sT1W_3D_TFE_701/IM-0005-0145.jpg]

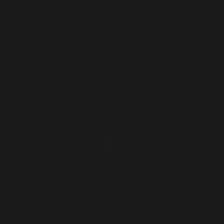

Supplement: Additional file 1: — 1. Complete image series of a transverse TSE T2w sequence in one rabbit. 2. Complete image series of a sagittal TSE T2w sequence in the same rabbit as in Additional file 1: 1. 3. Complete image series of a dorsal TSE T2w sequence in the same rabbit as in Additional file 1: 1. 4. Complete image series of a FLAIR longTR CLEAR sequence in the same rabbit as in Additional file 1: 1. 5. Complete image series of a precontrast T1w 3D (TFE SENSE) sequence in the same rabbit as in Additional file 1: 1. 6. Complete image series of a postcontrast T1w 3D (TFE SENSE) sequence in the same rabbit as in Additional file 1: 1. [file 13028_2015_139_MOESM1_ESM.zip › Brain_2200809/sT1W_3D_TFE_701/IM-0005-0146.jpg]

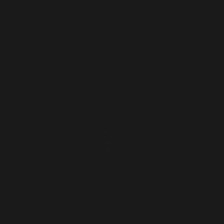

Supplement: Additional file 1: — 1. Complete image series of a transverse TSE T2w sequence in one rabbit. 2. Complete image series of a sagittal TSE T2w sequence in the same rabbit as in Additional file 1: 1. 3. Complete image series of a dorsal TSE T2w sequence in the same rabbit as in Additional file 1: 1. 4. Complete image series of a FLAIR longTR CLEAR sequence in the same rabbit as in Additional file 1: 1. 5. Complete image series of a precontrast T1w 3D (TFE SENSE) sequence in the same rabbit as in Additional file 1: 1. 6. Complete image series of a postcontrast T1w 3D (TFE SENSE) sequence in the same rabbit as in Additional file 1: 1. [file 13028_2015_139_MOESM1_ESM.zip › Brain_2200809/sT1W_3D_TFE_701/IM-0005-0147.jpg]

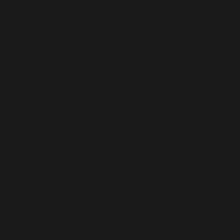

Supplement: Additional file 1: — 1. Complete image series of a transverse TSE T2w sequence in one rabbit. 2. Complete image series of a sagittal TSE T2w sequence in the same rabbit as in Additional file 1: 1. 3. Complete image series of a dorsal TSE T2w sequence in the same rabbit as in Additional file 1: 1. 4. Complete image series of a FLAIR longTR CLEAR sequence in the same rabbit as in Additional file 1: 1. 5. Complete image series of a precontrast T1w 3D (TFE SENSE) sequence in the same rabbit as in Additional file 1: 1. 6. Complete image series of a postcontrast T1w 3D (TFE SENSE) sequence in the same rabbit as in Additional file 1: 1. [file 13028_2015_139_MOESM1_ESM.zip › Brain_2200809/sT1W_3D_TFE_701/IM-0005-0148.jpg]

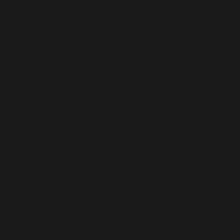

Supplement: Additional file 1: — 1. Complete image series of a transverse TSE T2w sequence in one rabbit. 2. Complete image series of a sagittal TSE T2w sequence in the same rabbit as in Additional file 1: 1. 3. Complete image series of a dorsal TSE T2w sequence in the same rabbit as in Additional file 1: 1. 4. Complete image series of a FLAIR longTR CLEAR sequence in the same rabbit as in Additional file 1: 1. 5. Complete image series of a precontrast T1w 3D (TFE SENSE) sequence in the same rabbit as in Additional file 1: 1. 6. Complete image series of a postcontrast T1w 3D (TFE SENSE) sequence in the same rabbit as in Additional file 1: 1. [file 13028_2015_139_MOESM1_ESM.zip › Brain_2200809/sT1W_3D_TFE_701/IM-0005-0149.jpg]

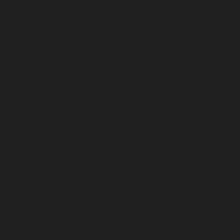

Supplement: Additional file 1: — 1. Complete image series of a transverse TSE T2w sequence in one rabbit. 2. Complete image series of a sagittal TSE T2w sequence in the same rabbit as in Additional file 1: 1. 3. Complete image series of a dorsal TSE T2w sequence in the same rabbit as in Additional file 1: 1. 4. Complete image series of a FLAIR longTR CLEAR sequence in the same rabbit as in Additional file 1: 1. 5. Complete image series of a precontrast T1w 3D (TFE SENSE) sequence in the same rabbit as in Additional file 1: 1. 6. Complete image series of a postcontrast T1w 3D (TFE SENSE) sequence in the same rabbit as in Additional file 1: 1. [file 13028_2015_139_MOESM1_ESM.zip › Brain_2200809/sT1W_3D_TFE_KM_1001/IM-0008-0001.jpg]

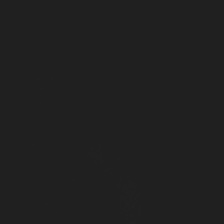

Supplement: Additional file 1: — 1. Complete image series of a transverse TSE T2w sequence in one rabbit. 2. Complete image series of a sagittal TSE T2w sequence in the same rabbit as in Additional file 1: 1. 3. Complete image series of a dorsal TSE T2w sequence in the same rabbit as in Additional file 1: 1. 4. Complete image series of a FLAIR longTR CLEAR sequence in the same rabbit as in Additional file 1: 1. 5. Complete image series of a precontrast T1w 3D (TFE SENSE) sequence in the same rabbit as in Additional file 1: 1. 6. Complete image series of a postcontrast T1w 3D (TFE SENSE) sequence in the same rabbit as in Additional file 1: 1. [file 13028_2015_139_MOESM1_ESM.zip › Brain_2200809/sT1W_3D_TFE_KM_1001/IM-0008-0002.jpg]

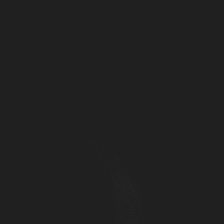

Supplement: Additional file 1: — 1. Complete image series of a transverse TSE T2w sequence in one rabbit. 2. Complete image series of a sagittal TSE T2w sequence in the same rabbit as in Additional file 1: 1. 3. Complete image series of a dorsal TSE T2w sequence in the same rabbit as in Additional file 1: 1. 4. Complete image series of a FLAIR longTR CLEAR sequence in the same rabbit as in Additional file 1: 1. 5. Complete image series of a precontrast T1w 3D (TFE SENSE) sequence in the same rabbit as in Additional file 1: 1. 6. Complete image series of a postcontrast T1w 3D (TFE SENSE) sequence in the same rabbit as in Additional file 1: 1. [file 13028_2015_139_MOESM1_ESM.zip › Brain_2200809/sT1W_3D_TFE_KM_1001/IM-0008-0003.jpg]

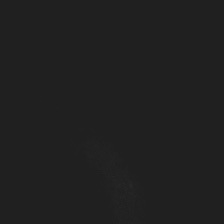

Supplement: Additional file 1: — 1. Complete image series of a transverse TSE T2w sequence in one rabbit. 2. Complete image series of a sagittal TSE T2w sequence in the same rabbit as in Additional file 1: 1. 3. Complete image series of a dorsal TSE T2w sequence in the same rabbit as in Additional file 1: 1. 4. Complete image series of a FLAIR longTR CLEAR sequence in the same rabbit as in Additional file 1: 1. 5. Complete image series of a precontrast T1w 3D (TFE SENSE) sequence in the same rabbit as in Additional file 1: 1. 6. Complete image series of a postcontrast T1w 3D (TFE SENSE) sequence in the same rabbit as in Additional file 1: 1. [file 13028_2015_139_MOESM1_ESM.zip › Brain_2200809/sT1W_3D_TFE_KM_1001/IM-0008-0004.jpg]

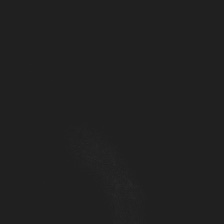

Supplement: Additional file 1: — 1. Complete image series of a transverse TSE T2w sequence in one rabbit. 2. Complete image series of a sagittal TSE T2w sequence in the same rabbit as in Additional file 1: 1. 3. Complete image series of a dorsal TSE T2w sequence in the same rabbit as in Additional file 1: 1. 4. Complete image series of a FLAIR longTR CLEAR sequence in the same rabbit as in Additional file 1: 1. 5. Complete image series of a precontrast T1w 3D (TFE SENSE) sequence in the same rabbit as in Additional file 1: 1. 6. Complete image series of a postcontrast T1w 3D (TFE SENSE) sequence in the same rabbit as in Additional file 1: 1. [file 13028_2015_139_MOESM1_ESM.zip › Brain_2200809/sT1W_3D_TFE_KM_1001/IM-0008-0005.jpg]

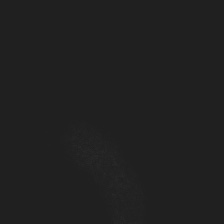

Supplement: Additional file 1: — 1. Complete image series of a transverse TSE T2w sequence in one rabbit. 2. Complete image series of a sagittal TSE T2w sequence in the same rabbit as in Additional file 1: 1. 3. Complete image series of a dorsal TSE T2w sequence in the same rabbit as in Additional file 1: 1. 4. Complete image series of a FLAIR longTR CLEAR sequence in the same rabbit as in Additional file 1: 1. 5. Complete image series of a precontrast T1w 3D (TFE SENSE) sequence in the same rabbit as in Additional file 1: 1. 6. Complete image series of a postcontrast T1w 3D (TFE SENSE) sequence in the same rabbit as in Additional file 1: 1. [file 13028_2015_139_MOESM1_ESM.zip › Brain_2200809/sT1W_3D_TFE_KM_1001/IM-0008-0006.jpg]

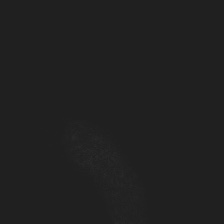

Supplement: Additional file 1: — 1. Complete image series of a transverse TSE T2w sequence in one rabbit. 2. Complete image series of a sagittal TSE T2w sequence in the same rabbit as in Additional file 1: 1. 3. Complete image series of a dorsal TSE T2w sequence in the same rabbit as in Additional file 1: 1. 4. Complete image series of a FLAIR longTR CLEAR sequence in the same rabbit as in Additional file 1: 1. 5. Complete image series of a precontrast T1w 3D (TFE SENSE) sequence in the same rabbit as in Additional file 1: 1. 6. Complete image series of a postcontrast T1w 3D (TFE SENSE) sequence in the same rabbit as in Additional file 1: 1. [file 13028_2015_139_MOESM1_ESM.zip › Brain_2200809/sT1W_3D_TFE_KM_1001/IM-0008-0007.jpg]

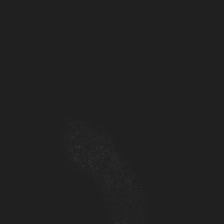

Supplement: Additional file 1: — 1. Complete image series of a transverse TSE T2w sequence in one rabbit. 2. Complete image series of a sagittal TSE T2w sequence in the same rabbit as in Additional file 1: 1. 3. Complete image series of a dorsal TSE T2w sequence in the same rabbit as in Additional file 1: 1. 4. Complete image series of a FLAIR longTR CLEAR sequence in the same rabbit as in Additional file 1: 1. 5. Complete image series of a precontrast T1w 3D (TFE SENSE) sequence in the same rabbit as in Additional file 1: 1. 6. Complete image series of a postcontrast T1w 3D (TFE SENSE) sequence in the same rabbit as in Additional file 1: 1. [file 13028_2015_139_MOESM1_ESM.zip › Brain_2200809/sT1W_3D_TFE_KM_1001/IM-0008-0008.jpg]

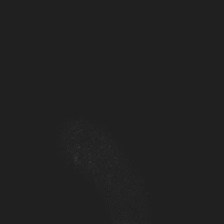

Supplement: Additional file 1: — 1. Complete image series of a transverse TSE T2w sequence in one rabbit. 2. Complete image series of a sagittal TSE T2w sequence in the same rabbit as in Additional file 1: 1. 3. Complete image series of a dorsal TSE T2w sequence in the same rabbit as in Additional file 1: 1. 4. Complete image series of a FLAIR longTR CLEAR sequence in the same rabbit as in Additional file 1: 1. 5. Complete image series of a precontrast T1w 3D (TFE SENSE) sequence in the same rabbit as in Additional file 1: 1. 6. Complete image series of a postcontrast T1w 3D (TFE SENSE) sequence in the same rabbit as in Additional file 1: 1. [file 13028_2015_139_MOESM1_ESM.zip › Brain_2200809/sT1W_3D_TFE_KM_1001/IM-0008-0009.jpg]

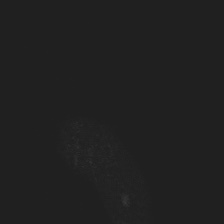

Supplement: Additional file 1: — 1. Complete image series of a transverse TSE T2w sequence in one rabbit. 2. Complete image series of a sagittal TSE T2w sequence in the same rabbit as in Additional file 1: 1. 3. Complete image series of a dorsal TSE T2w sequence in the same rabbit as in Additional file 1: 1. 4. Complete image series of a FLAIR longTR CLEAR sequence in the same rabbit as in Additional file 1: 1. 5. Complete image series of a precontrast T1w 3D (TFE SENSE) sequence in the same rabbit as in Additional file 1: 1. 6. Complete image series of a postcontrast T1w 3D (TFE SENSE) sequence in the same rabbit as in Additional file 1: 1. [file 13028_2015_139_MOESM1_ESM.zip › Brain_2200809/sT1W_3D_TFE_KM_1001/IM-0008-0010.jpg]

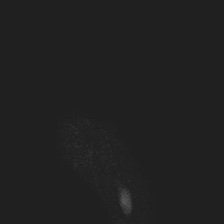

Supplement: Additional file 1: — 1. Complete image series of a transverse TSE T2w sequence in one rabbit. 2. Complete image series of a sagittal TSE T2w sequence in the same rabbit as in Additional file 1: 1. 3. Complete image series of a dorsal TSE T2w sequence in the same rabbit as in Additional file 1: 1. 4. Complete image series of a FLAIR longTR CLEAR sequence in the same rabbit as in Additional file 1: 1. 5. Complete image series of a precontrast T1w 3D (TFE SENSE) sequence in the same rabbit as in Additional file 1: 1. 6. Complete image series of a postcontrast T1w 3D (TFE SENSE) sequence in the same rabbit as in Additional file 1: 1. [file 13028_2015_139_MOESM1_ESM.zip › Brain_2200809/sT1W_3D_TFE_KM_1001/IM-0008-0011.jpg]

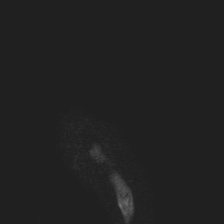

Supplement: Additional file 1: — 1. Complete image series of a transverse TSE T2w sequence in one rabbit. 2. Complete image series of a sagittal TSE T2w sequence in the same rabbit as in Additional file 1: 1. 3. Complete image series of a dorsal TSE T2w sequence in the same rabbit as in Additional file 1: 1. 4. Complete image series of a FLAIR longTR CLEAR sequence in the same rabbit as in Additional file 1: 1. 5. Complete image series of a precontrast T1w 3D (TFE SENSE) sequence in the same rabbit as in Additional file 1: 1. 6. Complete image series of a postcontrast T1w 3D (TFE SENSE) sequence in the same rabbit as in Additional file 1: 1. [file 13028_2015_139_MOESM1_ESM.zip › Brain_2200809/sT1W_3D_TFE_KM_1001/IM-0008-0012.jpg]

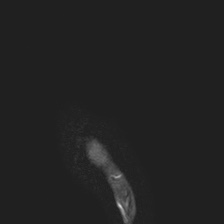

Supplement: Additional file 1: — 1. Complete image series of a transverse TSE T2w sequence in one rabbit. 2. Complete image series of a sagittal TSE T2w sequence in the same rabbit as in Additional file 1: 1. 3. Complete image series of a dorsal TSE T2w sequence in the same rabbit as in Additional file 1: 1. 4. Complete image series of a FLAIR longTR CLEAR sequence in the same rabbit as in Additional file 1: 1. 5. Complete image series of a precontrast T1w 3D (TFE SENSE) sequence in the same rabbit as in Additional file 1: 1. 6. Complete image series of a postcontrast T1w 3D (TFE SENSE) sequence in the same rabbit as in Additional file 1: 1. [file 13028_2015_139_MOESM1_ESM.zip › Brain_2200809/sT1W_3D_TFE_KM_1001/IM-0008-0013.jpg]

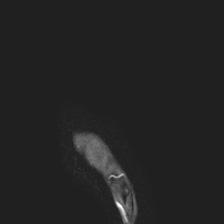

Supplement: Additional file 1: — 1. Complete image series of a transverse TSE T2w sequence in one rabbit. 2. Complete image series of a sagittal TSE T2w sequence in the same rabbit as in Additional file 1: 1. 3. Complete image series of a dorsal TSE T2w sequence in the same rabbit as in Additional file 1: 1. 4. Complete image series of a FLAIR longTR CLEAR sequence in the same rabbit as in Additional file 1: 1. 5. Complete image series of a precontrast T1w 3D (TFE SENSE) sequence in the same rabbit as in Additional file 1: 1. 6. Complete image series of a postcontrast T1w 3D (TFE SENSE) sequence in the same rabbit as in Additional file 1: 1. [file 13028_2015_139_MOESM1_ESM.zip › Brain_2200809/sT1W_3D_TFE_KM_1001/IM-0008-0014.jpg]

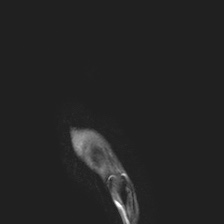

Supplement: Additional file 1: — 1. Complete image series of a transverse TSE T2w sequence in one rabbit. 2. Complete image series of a sagittal TSE T2w sequence in the same rabbit as in Additional file 1: 1. 3. Complete image series of a dorsal TSE T2w sequence in the same rabbit as in Additional file 1: 1. 4. Complete image series of a FLAIR longTR CLEAR sequence in the same rabbit as in Additional file 1: 1. 5. Complete image series of a precontrast T1w 3D (TFE SENSE) sequence in the same rabbit as in Additional file 1: 1. 6. Complete image series of a postcontrast T1w 3D (TFE SENSE) sequence in the same rabbit as in Additional file 1: 1. [file 13028_2015_139_MOESM1_ESM.zip › Brain_2200809/sT1W_3D_TFE_KM_1001/IM-0008-0015.jpg]

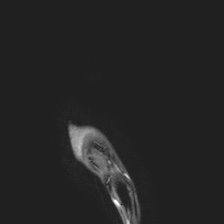

Supplement: Additional file 1: — 1. Complete image series of a transverse TSE T2w sequence in one rabbit. 2. Complete image series of a sagittal TSE T2w sequence in the same rabbit as in Additional file 1: 1. 3. Complete image series of a dorsal TSE T2w sequence in the same rabbit as in Additional file 1: 1. 4. Complete image series of a FLAIR longTR CLEAR sequence in the same rabbit as in Additional file 1: 1. 5. Complete image series of a precontrast T1w 3D (TFE SENSE) sequence in the same rabbit as in Additional file 1: 1. 6. Complete image series of a postcontrast T1w 3D (TFE SENSE) sequence in the same rabbit as in Additional file 1: 1. [file 13028_2015_139_MOESM1_ESM.zip › Brain_2200809/sT1W_3D_TFE_KM_1001/IM-0008-0016.jpg]

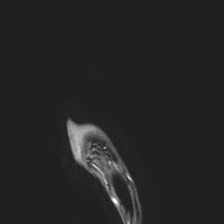

Supplement: Additional file 1: — 1. Complete image series of a transverse TSE T2w sequence in one rabbit. 2. Complete image series of a sagittal TSE T2w sequence in the same rabbit as in Additional file 1: 1. 3. Complete image series of a dorsal TSE T2w sequence in the same rabbit as in Additional file 1: 1. 4. Complete image series of a FLAIR longTR CLEAR sequence in the same rabbit as in Additional file 1: 1. 5. Complete image series of a precontrast T1w 3D (TFE SENSE) sequence in the same rabbit as in Additional file 1: 1. 6. Complete image series of a postcontrast T1w 3D (TFE SENSE) sequence in the same rabbit as in Additional file 1: 1. [file 13028_2015_139_MOESM1_ESM.zip › Brain_2200809/sT1W_3D_TFE_KM_1001/IM-0008-0017.jpg]

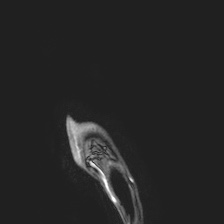

Supplement: Additional file 1: — 1. Complete image series of a transverse TSE T2w sequence in one rabbit. 2. Complete image series of a sagittal TSE T2w sequence in the same rabbit as in Additional file 1: 1. 3. Complete image series of a dorsal TSE T2w sequence in the same rabbit as in Additional file 1: 1. 4. Complete image series of a FLAIR longTR CLEAR sequence in the same rabbit as in Additional file 1: 1. 5. Complete image series of a precontrast T1w 3D (TFE SENSE) sequence in the same rabbit as in Additional file 1: 1. 6. Complete image series of a postcontrast T1w 3D (TFE SENSE) sequence in the same rabbit as in Additional file 1: 1. [file 13028_2015_139_MOESM1_ESM.zip › Brain_2200809/sT1W_3D_TFE_KM_1001/IM-0008-0018.jpg]

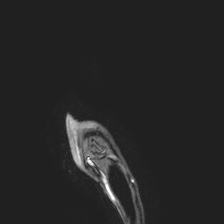

Supplement: Additional file 1: — 1. Complete image series of a transverse TSE T2w sequence in one rabbit. 2. Complete image series of a sagittal TSE T2w sequence in the same rabbit as in Additional file 1: 1. 3. Complete image series of a dorsal TSE T2w sequence in the same rabbit as in Additional file 1: 1. 4. Complete image series of a FLAIR longTR CLEAR sequence in the same rabbit as in Additional file 1: 1. 5. Complete image series of a precontrast T1w 3D (TFE SENSE) sequence in the same rabbit as in Additional file 1: 1. 6. Complete image series of a postcontrast T1w 3D (TFE SENSE) sequence in the same rabbit as in Additional file 1: 1. [file 13028_2015_139_MOESM1_ESM.zip › Brain_2200809/sT1W_3D_TFE_KM_1001/IM-0008-0019.jpg]

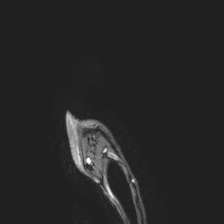

Supplement: Additional file 1: — 1. Complete image series of a transverse TSE T2w sequence in one rabbit. 2. Complete image series of a sagittal TSE T2w sequence in the same rabbit as in Additional file 1: 1. 3. Complete image series of a dorsal TSE T2w sequence in the same rabbit as in Additional file 1: 1. 4. Complete image series of a FLAIR longTR CLEAR sequence in the same rabbit as in Additional file 1: 1. 5. Complete image series of a precontrast T1w 3D (TFE SENSE) sequence in the same rabbit as in Additional file 1: 1. 6. Complete image series of a postcontrast T1w 3D (TFE SENSE) sequence in the same rabbit as in Additional file 1: 1. [file 13028_2015_139_MOESM1_ESM.zip › Brain_2200809/sT1W_3D_TFE_KM_1001/IM-0008-0020.jpg]

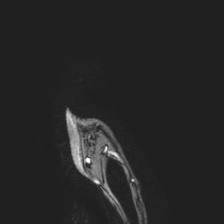

Supplement: Additional file 1: — 1. Complete image series of a transverse TSE T2w sequence in one rabbit. 2. Complete image series of a sagittal TSE T2w sequence in the same rabbit as in Additional file 1: 1. 3. Complete image series of a dorsal TSE T2w sequence in the same rabbit as in Additional file 1: 1. 4. Complete image series of a FLAIR longTR CLEAR sequence in the same rabbit as in Additional file 1: 1. 5. Complete image series of a precontrast T1w 3D (TFE SENSE) sequence in the same rabbit as in Additional file 1: 1. 6. Complete image series of a postcontrast T1w 3D (TFE SENSE) sequence in the same rabbit as in Additional file 1: 1. [file 13028_2015_139_MOESM1_ESM.zip › Brain_2200809/sT1W_3D_TFE_KM_1001/IM-0008-0021.jpg]

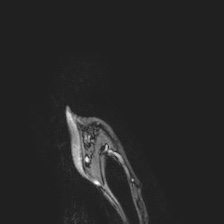

Supplement: Additional file 1: — 1. Complete image series of a transverse TSE T2w sequence in one rabbit. 2. Complete image series of a sagittal TSE T2w sequence in the same rabbit as in Additional file 1: 1. 3. Complete image series of a dorsal TSE T2w sequence in the same rabbit as in Additional file 1: 1. 4. Complete image series of a FLAIR longTR CLEAR sequence in the same rabbit as in Additional file 1: 1. 5. Complete image series of a precontrast T1w 3D (TFE SENSE) sequence in the same rabbit as in Additional file 1: 1. 6. Complete image series of a postcontrast T1w 3D (TFE SENSE) sequence in the same rabbit as in Additional file 1: 1. [file 13028_2015_139_MOESM1_ESM.zip › Brain_2200809/sT1W_3D_TFE_KM_1001/IM-0008-0022.jpg]

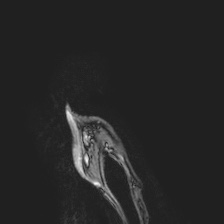

Supplement: Additional file 1: — 1. Complete image series of a transverse TSE T2w sequence in one rabbit. 2. Complete image series of a sagittal TSE T2w sequence in the same rabbit as in Additional file 1: 1. 3. Complete image series of a dorsal TSE T2w sequence in the same rabbit as in Additional file 1: 1. 4. Complete image series of a FLAIR longTR CLEAR sequence in the same rabbit as in Additional file 1: 1. 5. Complete image series of a precontrast T1w 3D (TFE SENSE) sequence in the same rabbit as in Additional file 1: 1. 6. Complete image series of a postcontrast T1w 3D (TFE SENSE) sequence in the same rabbit as in Additional file 1: 1. [file 13028_2015_139_MOESM1_ESM.zip › Brain_2200809/sT1W_3D_TFE_KM_1001/IM-0008-0023.jpg]

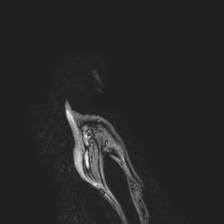

Supplement: Additional file 1: — 1. Complete image series of a transverse TSE T2w sequence in one rabbit. 2. Complete image series of a sagittal TSE T2w sequence in the same rabbit as in Additional file 1: 1. 3. Complete image series of a dorsal TSE T2w sequence in the same rabbit as in Additional file 1: 1. 4. Complete image series of a FLAIR longTR CLEAR sequence in the same rabbit as in Additional file 1: 1. 5. Complete image series of a precontrast T1w 3D (TFE SENSE) sequence in the same rabbit as in Additional file 1: 1. 6. Complete image series of a postcontrast T1w 3D (TFE SENSE) sequence in the same rabbit as in Additional file 1: 1. [file 13028_2015_139_MOESM1_ESM.zip › Brain_2200809/sT1W_3D_TFE_KM_1001/IM-0008-0024.jpg]

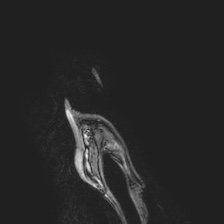

Supplement: Additional file 1: — 1. Complete image series of a transverse TSE T2w sequence in one rabbit. 2. Complete image series of a sagittal TSE T2w sequence in the same rabbit as in Additional file 1: 1. 3. Complete image series of a dorsal TSE T2w sequence in the same rabbit as in Additional file 1: 1. 4. Complete image series of a FLAIR longTR CLEAR sequence in the same rabbit as in Additional file 1: 1. 5. Complete image series of a precontrast T1w 3D (TFE SENSE) sequence in the same rabbit as in Additional file 1: 1. 6. Complete image series of a postcontrast T1w 3D (TFE SENSE) sequence in the same rabbit as in Additional file 1: 1. [file 13028_2015_139_MOESM1_ESM.zip › Brain_2200809/sT1W_3D_TFE_KM_1001/IM-0008-0025.jpg]

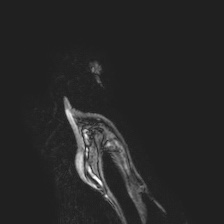

Supplement: Additional file 1: — 1. Complete image series of a transverse TSE T2w sequence in one rabbit. 2. Complete image series of a sagittal TSE T2w sequence in the same rabbit as in Additional file 1: 1. 3. Complete image series of a dorsal TSE T2w sequence in the same rabbit as in Additional file 1: 1. 4. Complete image series of a FLAIR longTR CLEAR sequence in the same rabbit as in Additional file 1: 1. 5. Complete image series of a precontrast T1w 3D (TFE SENSE) sequence in the same rabbit as in Additional file 1: 1. 6. Complete image series of a postcontrast T1w 3D (TFE SENSE) sequence in the same rabbit as in Additional file 1: 1. [file 13028_2015_139_MOESM1_ESM.zip › Brain_2200809/sT1W_3D_TFE_KM_1001/IM-0008-0026.jpg]

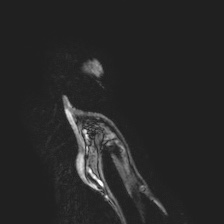

Supplement: Additional file 1: — 1. Complete image series of a transverse TSE T2w sequence in one rabbit. 2. Complete image series of a sagittal TSE T2w sequence in the same rabbit as in Additional file 1: 1. 3. Complete image series of a dorsal TSE T2w sequence in the same rabbit as in Additional file 1: 1. 4. Complete image series of a FLAIR longTR CLEAR sequence in the same rabbit as in Additional file 1: 1. 5. Complete image series of a precontrast T1w 3D (TFE SENSE) sequence in the same rabbit as in Additional file 1: 1. 6. Complete image series of a postcontrast T1w 3D (TFE SENSE) sequence in the same rabbit as in Additional file 1: 1. [file 13028_2015_139_MOESM1_ESM.zip › Brain_2200809/sT1W_3D_TFE_KM_1001/IM-0008-0027.jpg]

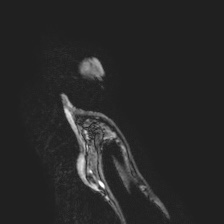

Supplement: Additional file 1: — 1. Complete image series of a transverse TSE T2w sequence in one rabbit. 2. Complete image series of a sagittal TSE T2w sequence in the same rabbit as in Additional file 1: 1. 3. Complete image series of a dorsal TSE T2w sequence in the same rabbit as in Additional file 1: 1. 4. Complete image series of a FLAIR longTR CLEAR sequence in the same rabbit as in Additional file 1: 1. 5. Complete image series of a precontrast T1w 3D (TFE SENSE) sequence in the same rabbit as in Additional file 1: 1. 6. Complete image series of a postcontrast T1w 3D (TFE SENSE) sequence in the same rabbit as in Additional file 1: 1. [file 13028_2015_139_MOESM1_ESM.zip › Brain_2200809/sT1W_3D_TFE_KM_1001/IM-0008-0028.jpg]

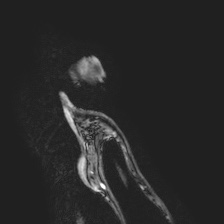

Supplement: Additional file 1: — 1. Complete image series of a transverse TSE T2w sequence in one rabbit. 2. Complete image series of a sagittal TSE T2w sequence in the same rabbit as in Additional file 1: 1. 3. Complete image series of a dorsal TSE T2w sequence in the same rabbit as in Additional file 1: 1. 4. Complete image series of a FLAIR longTR CLEAR sequence in the same rabbit as in Additional file 1: 1. 5. Complete image series of a precontrast T1w 3D (TFE SENSE) sequence in the same rabbit as in Additional file 1: 1. 6. Complete image series of a postcontrast T1w 3D (TFE SENSE) sequence in the same rabbit as in Additional file 1: 1. [file 13028_2015_139_MOESM1_ESM.zip › Brain_2200809/sT1W_3D_TFE_KM_1001/IM-0008-0029.jpg]

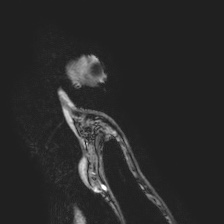

Supplement: Additional file 1: — 1. Complete image series of a transverse TSE T2w sequence in one rabbit. 2. Complete image series of a sagittal TSE T2w sequence in the same rabbit as in Additional file 1: 1. 3. Complete image series of a dorsal TSE T2w sequence in the same rabbit as in Additional file 1: 1. 4. Complete image series of a FLAIR longTR CLEAR sequence in the same rabbit as in Additional file 1: 1. 5. Complete image series of a precontrast T1w 3D (TFE SENSE) sequence in the same rabbit as in Additional file 1: 1. 6. Complete image series of a postcontrast T1w 3D (TFE SENSE) sequence in the same rabbit as in Additional file 1: 1. [file 13028_2015_139_MOESM1_ESM.zip › Brain_2200809/sT1W_3D_TFE_KM_1001/IM-0008-0030.jpg]
