# Supplementary figures and images for: Magnetic resonance imaging anatomy of the rabbit brain at 3 T (part 1 of 2)
Source: Acta Vet Scand. 2015 Aug 28;57(1):47. doi: 10.1186/s13028-015-0139-6 (PMC4551377; doi:10.1186/s13028-015-0139-6)

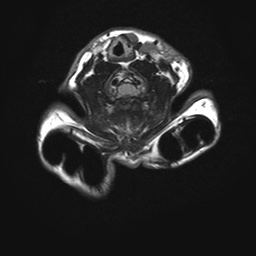

Supplement: Additional file 1: — 1. Complete image series of a transverse TSE T2w sequence in one rabbit. 2. Complete image series of a sagittal TSE T2w sequence in the same rabbit as in Additional file 1: 1. 3. Complete image series of a dorsal TSE T2w sequence in the same rabbit as in Additional file 1: 1. 4. Complete image series of a FLAIR longTR CLEAR sequence in the same rabbit as in Additional file 1: 1. 5. Complete image series of a precontrast T1w 3D (TFE SENSE) sequence in the same rabbit as in Additional file 1: 1. 6. Complete image series of a postcontrast T1w 3D (TFE SENSE) sequence in the same rabbit as in Additional file 1: 1. [file 13028_2015_139_MOESM1_ESM.zip › Brain_2200809/FLAIR_LongTR_401/IM-0002-0001.jpg]

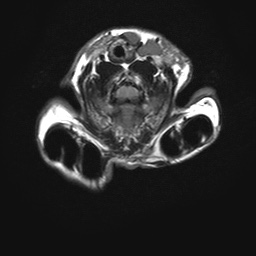

Supplement: Additional file 1: — 1. Complete image series of a transverse TSE T2w sequence in one rabbit. 2. Complete image series of a sagittal TSE T2w sequence in the same rabbit as in Additional file 1: 1. 3. Complete image series of a dorsal TSE T2w sequence in the same rabbit as in Additional file 1: 1. 4. Complete image series of a FLAIR longTR CLEAR sequence in the same rabbit as in Additional file 1: 1. 5. Complete image series of a precontrast T1w 3D (TFE SENSE) sequence in the same rabbit as in Additional file 1: 1. 6. Complete image series of a postcontrast T1w 3D (TFE SENSE) sequence in the same rabbit as in Additional file 1: 1. [file 13028_2015_139_MOESM1_ESM.zip › Brain_2200809/FLAIR_LongTR_401/IM-0002-0002.jpg]

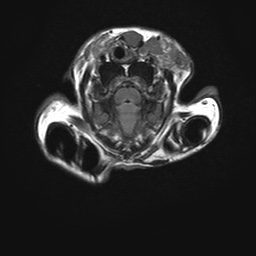

Supplement: Additional file 1: — 1. Complete image series of a transverse TSE T2w sequence in one rabbit. 2. Complete image series of a sagittal TSE T2w sequence in the same rabbit as in Additional file 1: 1. 3. Complete image series of a dorsal TSE T2w sequence in the same rabbit as in Additional file 1: 1. 4. Complete image series of a FLAIR longTR CLEAR sequence in the same rabbit as in Additional file 1: 1. 5. Complete image series of a precontrast T1w 3D (TFE SENSE) sequence in the same rabbit as in Additional file 1: 1. 6. Complete image series of a postcontrast T1w 3D (TFE SENSE) sequence in the same rabbit as in Additional file 1: 1. [file 13028_2015_139_MOESM1_ESM.zip › Brain_2200809/FLAIR_LongTR_401/IM-0002-0003.jpg]

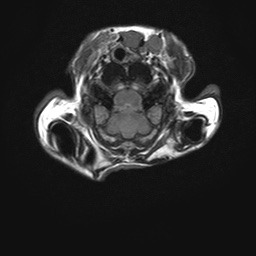

Supplement: Additional file 1: — 1. Complete image series of a transverse TSE T2w sequence in one rabbit. 2. Complete image series of a sagittal TSE T2w sequence in the same rabbit as in Additional file 1: 1. 3. Complete image series of a dorsal TSE T2w sequence in the same rabbit as in Additional file 1: 1. 4. Complete image series of a FLAIR longTR CLEAR sequence in the same rabbit as in Additional file 1: 1. 5. Complete image series of a precontrast T1w 3D (TFE SENSE) sequence in the same rabbit as in Additional file 1: 1. 6. Complete image series of a postcontrast T1w 3D (TFE SENSE) sequence in the same rabbit as in Additional file 1: 1. [file 13028_2015_139_MOESM1_ESM.zip › Brain_2200809/FLAIR_LongTR_401/IM-0002-0004.jpg]

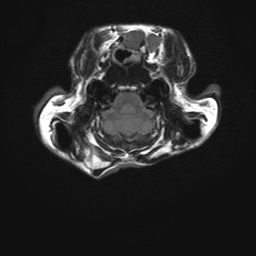

Supplement: Additional file 1: — 1. Complete image series of a transverse TSE T2w sequence in one rabbit. 2. Complete image series of a sagittal TSE T2w sequence in the same rabbit as in Additional file 1: 1. 3. Complete image series of a dorsal TSE T2w sequence in the same rabbit as in Additional file 1: 1. 4. Complete image series of a FLAIR longTR CLEAR sequence in the same rabbit as in Additional file 1: 1. 5. Complete image series of a precontrast T1w 3D (TFE SENSE) sequence in the same rabbit as in Additional file 1: 1. 6. Complete image series of a postcontrast T1w 3D (TFE SENSE) sequence in the same rabbit as in Additional file 1: 1. [file 13028_2015_139_MOESM1_ESM.zip › Brain_2200809/FLAIR_LongTR_401/IM-0002-0005.jpg]

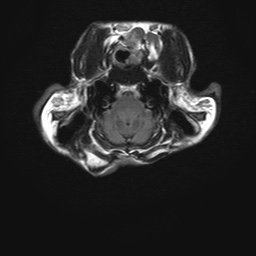

Supplement: Additional file 1: — 1. Complete image series of a transverse TSE T2w sequence in one rabbit. 2. Complete image series of a sagittal TSE T2w sequence in the same rabbit as in Additional file 1: 1. 3. Complete image series of a dorsal TSE T2w sequence in the same rabbit as in Additional file 1: 1. 4. Complete image series of a FLAIR longTR CLEAR sequence in the same rabbit as in Additional file 1: 1. 5. Complete image series of a precontrast T1w 3D (TFE SENSE) sequence in the same rabbit as in Additional file 1: 1. 6. Complete image series of a postcontrast T1w 3D (TFE SENSE) sequence in the same rabbit as in Additional file 1: 1. [file 13028_2015_139_MOESM1_ESM.zip › Brain_2200809/FLAIR_LongTR_401/IM-0002-0006.jpg]

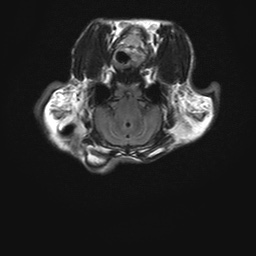

Supplement: Additional file 1: — 1. Complete image series of a transverse TSE T2w sequence in one rabbit. 2. Complete image series of a sagittal TSE T2w sequence in the same rabbit as in Additional file 1: 1. 3. Complete image series of a dorsal TSE T2w sequence in the same rabbit as in Additional file 1: 1. 4. Complete image series of a FLAIR longTR CLEAR sequence in the same rabbit as in Additional file 1: 1. 5. Complete image series of a precontrast T1w 3D (TFE SENSE) sequence in the same rabbit as in Additional file 1: 1. 6. Complete image series of a postcontrast T1w 3D (TFE SENSE) sequence in the same rabbit as in Additional file 1: 1. [file 13028_2015_139_MOESM1_ESM.zip › Brain_2200809/FLAIR_LongTR_401/IM-0002-0007.jpg]

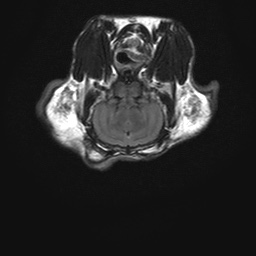

Supplement: Additional file 1: — 1. Complete image series of a transverse TSE T2w sequence in one rabbit. 2. Complete image series of a sagittal TSE T2w sequence in the same rabbit as in Additional file 1: 1. 3. Complete image series of a dorsal TSE T2w sequence in the same rabbit as in Additional file 1: 1. 4. Complete image series of a FLAIR longTR CLEAR sequence in the same rabbit as in Additional file 1: 1. 5. Complete image series of a precontrast T1w 3D (TFE SENSE) sequence in the same rabbit as in Additional file 1: 1. 6. Complete image series of a postcontrast T1w 3D (TFE SENSE) sequence in the same rabbit as in Additional file 1: 1. [file 13028_2015_139_MOESM1_ESM.zip › Brain_2200809/FLAIR_LongTR_401/IM-0002-0008.jpg]

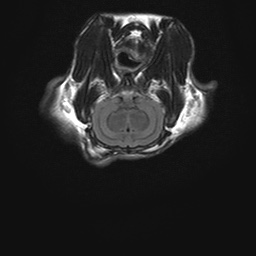

Supplement: Additional file 1: — 1. Complete image series of a transverse TSE T2w sequence in one rabbit. 2. Complete image series of a sagittal TSE T2w sequence in the same rabbit as in Additional file 1: 1. 3. Complete image series of a dorsal TSE T2w sequence in the same rabbit as in Additional file 1: 1. 4. Complete image series of a FLAIR longTR CLEAR sequence in the same rabbit as in Additional file 1: 1. 5. Complete image series of a precontrast T1w 3D (TFE SENSE) sequence in the same rabbit as in Additional file 1: 1. 6. Complete image series of a postcontrast T1w 3D (TFE SENSE) sequence in the same rabbit as in Additional file 1: 1. [file 13028_2015_139_MOESM1_ESM.zip › Brain_2200809/FLAIR_LongTR_401/IM-0002-0009.jpg]

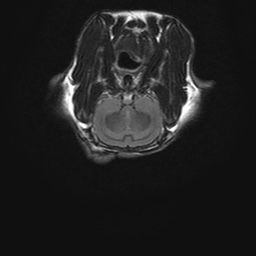

Supplement: Additional file 1: — 1. Complete image series of a transverse TSE T2w sequence in one rabbit. 2. Complete image series of a sagittal TSE T2w sequence in the same rabbit as in Additional file 1: 1. 3. Complete image series of a dorsal TSE T2w sequence in the same rabbit as in Additional file 1: 1. 4. Complete image series of a FLAIR longTR CLEAR sequence in the same rabbit as in Additional file 1: 1. 5. Complete image series of a precontrast T1w 3D (TFE SENSE) sequence in the same rabbit as in Additional file 1: 1. 6. Complete image series of a postcontrast T1w 3D (TFE SENSE) sequence in the same rabbit as in Additional file 1: 1. [file 13028_2015_139_MOESM1_ESM.zip › Brain_2200809/FLAIR_LongTR_401/IM-0002-0010.jpg]

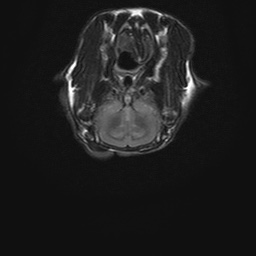

Supplement: Additional file 1: — 1. Complete image series of a transverse TSE T2w sequence in one rabbit. 2. Complete image series of a sagittal TSE T2w sequence in the same rabbit as in Additional file 1: 1. 3. Complete image series of a dorsal TSE T2w sequence in the same rabbit as in Additional file 1: 1. 4. Complete image series of a FLAIR longTR CLEAR sequence in the same rabbit as in Additional file 1: 1. 5. Complete image series of a precontrast T1w 3D (TFE SENSE) sequence in the same rabbit as in Additional file 1: 1. 6. Complete image series of a postcontrast T1w 3D (TFE SENSE) sequence in the same rabbit as in Additional file 1: 1. [file 13028_2015_139_MOESM1_ESM.zip › Brain_2200809/FLAIR_LongTR_401/IM-0002-0011.jpg]

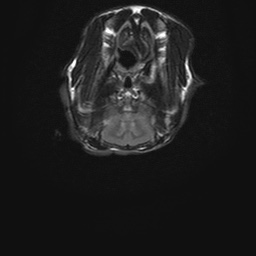

Supplement: Additional file 1: — 1. Complete image series of a transverse TSE T2w sequence in one rabbit. 2. Complete image series of a sagittal TSE T2w sequence in the same rabbit as in Additional file 1: 1. 3. Complete image series of a dorsal TSE T2w sequence in the same rabbit as in Additional file 1: 1. 4. Complete image series of a FLAIR longTR CLEAR sequence in the same rabbit as in Additional file 1: 1. 5. Complete image series of a precontrast T1w 3D (TFE SENSE) sequence in the same rabbit as in Additional file 1: 1. 6. Complete image series of a postcontrast T1w 3D (TFE SENSE) sequence in the same rabbit as in Additional file 1: 1. [file 13028_2015_139_MOESM1_ESM.zip › Brain_2200809/FLAIR_LongTR_401/IM-0002-0012.jpg]

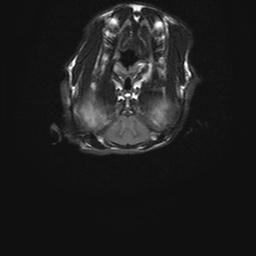

Supplement: Additional file 1: — 1. Complete image series of a transverse TSE T2w sequence in one rabbit. 2. Complete image series of a sagittal TSE T2w sequence in the same rabbit as in Additional file 1: 1. 3. Complete image series of a dorsal TSE T2w sequence in the same rabbit as in Additional file 1: 1. 4. Complete image series of a FLAIR longTR CLEAR sequence in the same rabbit as in Additional file 1: 1. 5. Complete image series of a precontrast T1w 3D (TFE SENSE) sequence in the same rabbit as in Additional file 1: 1. 6. Complete image series of a postcontrast T1w 3D (TFE SENSE) sequence in the same rabbit as in Additional file 1: 1. [file 13028_2015_139_MOESM1_ESM.zip › Brain_2200809/FLAIR_LongTR_401/IM-0002-0013.jpg]

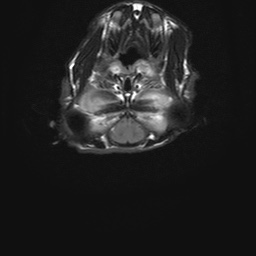

Supplement: Additional file 1: — 1. Complete image series of a transverse TSE T2w sequence in one rabbit. 2. Complete image series of a sagittal TSE T2w sequence in the same rabbit as in Additional file 1: 1. 3. Complete image series of a dorsal TSE T2w sequence in the same rabbit as in Additional file 1: 1. 4. Complete image series of a FLAIR longTR CLEAR sequence in the same rabbit as in Additional file 1: 1. 5. Complete image series of a precontrast T1w 3D (TFE SENSE) sequence in the same rabbit as in Additional file 1: 1. 6. Complete image series of a postcontrast T1w 3D (TFE SENSE) sequence in the same rabbit as in Additional file 1: 1. [file 13028_2015_139_MOESM1_ESM.zip › Brain_2200809/FLAIR_LongTR_401/IM-0002-0014.jpg]

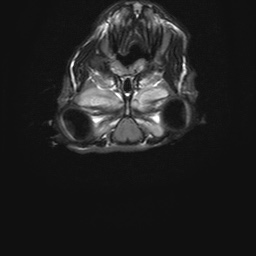

Supplement: Additional file 1: — 1. Complete image series of a transverse TSE T2w sequence in one rabbit. 2. Complete image series of a sagittal TSE T2w sequence in the same rabbit as in Additional file 1: 1. 3. Complete image series of a dorsal TSE T2w sequence in the same rabbit as in Additional file 1: 1. 4. Complete image series of a FLAIR longTR CLEAR sequence in the same rabbit as in Additional file 1: 1. 5. Complete image series of a precontrast T1w 3D (TFE SENSE) sequence in the same rabbit as in Additional file 1: 1. 6. Complete image series of a postcontrast T1w 3D (TFE SENSE) sequence in the same rabbit as in Additional file 1: 1. [file 13028_2015_139_MOESM1_ESM.zip › Brain_2200809/FLAIR_LongTR_401/IM-0002-0015.jpg]

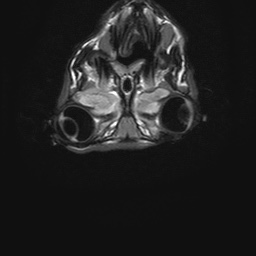

Supplement: Additional file 1: — 1. Complete image series of a transverse TSE T2w sequence in one rabbit. 2. Complete image series of a sagittal TSE T2w sequence in the same rabbit as in Additional file 1: 1. 3. Complete image series of a dorsal TSE T2w sequence in the same rabbit as in Additional file 1: 1. 4. Complete image series of a FLAIR longTR CLEAR sequence in the same rabbit as in Additional file 1: 1. 5. Complete image series of a precontrast T1w 3D (TFE SENSE) sequence in the same rabbit as in Additional file 1: 1. 6. Complete image series of a postcontrast T1w 3D (TFE SENSE) sequence in the same rabbit as in Additional file 1: 1. [file 13028_2015_139_MOESM1_ESM.zip › Brain_2200809/FLAIR_LongTR_401/IM-0002-0016.jpg]

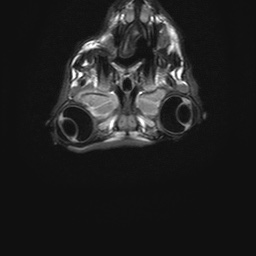

Supplement: Additional file 1: — 1. Complete image series of a transverse TSE T2w sequence in one rabbit. 2. Complete image series of a sagittal TSE T2w sequence in the same rabbit as in Additional file 1: 1. 3. Complete image series of a dorsal TSE T2w sequence in the same rabbit as in Additional file 1: 1. 4. Complete image series of a FLAIR longTR CLEAR sequence in the same rabbit as in Additional file 1: 1. 5. Complete image series of a precontrast T1w 3D (TFE SENSE) sequence in the same rabbit as in Additional file 1: 1. 6. Complete image series of a postcontrast T1w 3D (TFE SENSE) sequence in the same rabbit as in Additional file 1: 1. [file 13028_2015_139_MOESM1_ESM.zip › Brain_2200809/FLAIR_LongTR_401/IM-0002-0017.jpg]

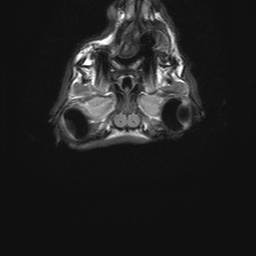

Supplement: Additional file 1: — 1. Complete image series of a transverse TSE T2w sequence in one rabbit. 2. Complete image series of a sagittal TSE T2w sequence in the same rabbit as in Additional file 1: 1. 3. Complete image series of a dorsal TSE T2w sequence in the same rabbit as in Additional file 1: 1. 4. Complete image series of a FLAIR longTR CLEAR sequence in the same rabbit as in Additional file 1: 1. 5. Complete image series of a precontrast T1w 3D (TFE SENSE) sequence in the same rabbit as in Additional file 1: 1. 6. Complete image series of a postcontrast T1w 3D (TFE SENSE) sequence in the same rabbit as in Additional file 1: 1. [file 13028_2015_139_MOESM1_ESM.zip › Brain_2200809/FLAIR_LongTR_401/IM-0002-0018.jpg]

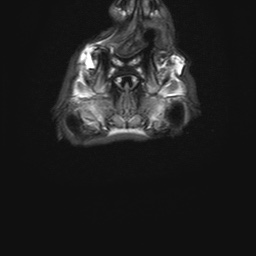

Supplement: Additional file 1: — 1. Complete image series of a transverse TSE T2w sequence in one rabbit. 2. Complete image series of a sagittal TSE T2w sequence in the same rabbit as in Additional file 1: 1. 3. Complete image series of a dorsal TSE T2w sequence in the same rabbit as in Additional file 1: 1. 4. Complete image series of a FLAIR longTR CLEAR sequence in the same rabbit as in Additional file 1: 1. 5. Complete image series of a precontrast T1w 3D (TFE SENSE) sequence in the same rabbit as in Additional file 1: 1. 6. Complete image series of a postcontrast T1w 3D (TFE SENSE) sequence in the same rabbit as in Additional file 1: 1. [file 13028_2015_139_MOESM1_ESM.zip › Brain_2200809/FLAIR_LongTR_401/IM-0002-0019.jpg]

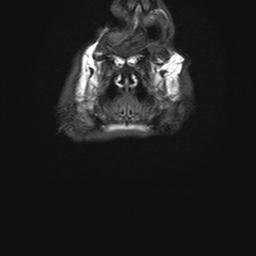

Supplement: Additional file 1: — 1. Complete image series of a transverse TSE T2w sequence in one rabbit. 2. Complete image series of a sagittal TSE T2w sequence in the same rabbit as in Additional file 1: 1. 3. Complete image series of a dorsal TSE T2w sequence in the same rabbit as in Additional file 1: 1. 4. Complete image series of a FLAIR longTR CLEAR sequence in the same rabbit as in Additional file 1: 1. 5. Complete image series of a precontrast T1w 3D (TFE SENSE) sequence in the same rabbit as in Additional file 1: 1. 6. Complete image series of a postcontrast T1w 3D (TFE SENSE) sequence in the same rabbit as in Additional file 1: 1. [file 13028_2015_139_MOESM1_ESM.zip › Brain_2200809/FLAIR_LongTR_401/IM-0002-0020.jpg]

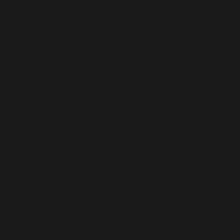

Supplement: Additional file 1: — 1. Complete image series of a transverse TSE T2w sequence in one rabbit. 2. Complete image series of a sagittal TSE T2w sequence in the same rabbit as in Additional file 1: 1. 3. Complete image series of a dorsal TSE T2w sequence in the same rabbit as in Additional file 1: 1. 4. Complete image series of a FLAIR longTR CLEAR sequence in the same rabbit as in Additional file 1: 1. 5. Complete image series of a precontrast T1w 3D (TFE SENSE) sequence in the same rabbit as in Additional file 1: 1. 6. Complete image series of a postcontrast T1w 3D (TFE SENSE) sequence in the same rabbit as in Additional file 1: 1. [file 13028_2015_139_MOESM1_ESM.zip › Brain_2200809/sT1W_3D_TFE_701/IM-0005-0001.jpg]

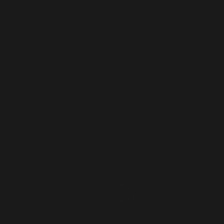

Supplement: Additional file 1: — 1. Complete image series of a transverse TSE T2w sequence in one rabbit. 2. Complete image series of a sagittal TSE T2w sequence in the same rabbit as in Additional file 1: 1. 3. Complete image series of a dorsal TSE T2w sequence in the same rabbit as in Additional file 1: 1. 4. Complete image series of a FLAIR longTR CLEAR sequence in the same rabbit as in Additional file 1: 1. 5. Complete image series of a precontrast T1w 3D (TFE SENSE) sequence in the same rabbit as in Additional file 1: 1. 6. Complete image series of a postcontrast T1w 3D (TFE SENSE) sequence in the same rabbit as in Additional file 1: 1. [file 13028_2015_139_MOESM1_ESM.zip › Brain_2200809/sT1W_3D_TFE_701/IM-0005-0002.jpg]

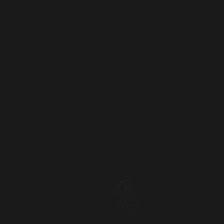

Supplement: Additional file 1: — 1. Complete image series of a transverse TSE T2w sequence in one rabbit. 2. Complete image series of a sagittal TSE T2w sequence in the same rabbit as in Additional file 1: 1. 3. Complete image series of a dorsal TSE T2w sequence in the same rabbit as in Additional file 1: 1. 4. Complete image series of a FLAIR longTR CLEAR sequence in the same rabbit as in Additional file 1: 1. 5. Complete image series of a precontrast T1w 3D (TFE SENSE) sequence in the same rabbit as in Additional file 1: 1. 6. Complete image series of a postcontrast T1w 3D (TFE SENSE) sequence in the same rabbit as in Additional file 1: 1. [file 13028_2015_139_MOESM1_ESM.zip › Brain_2200809/sT1W_3D_TFE_701/IM-0005-0003.jpg]

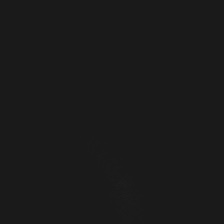

Supplement: Additional file 1: — 1. Complete image series of a transverse TSE T2w sequence in one rabbit. 2. Complete image series of a sagittal TSE T2w sequence in the same rabbit as in Additional file 1: 1. 3. Complete image series of a dorsal TSE T2w sequence in the same rabbit as in Additional file 1: 1. 4. Complete image series of a FLAIR longTR CLEAR sequence in the same rabbit as in Additional file 1: 1. 5. Complete image series of a precontrast T1w 3D (TFE SENSE) sequence in the same rabbit as in Additional file 1: 1. 6. Complete image series of a postcontrast T1w 3D (TFE SENSE) sequence in the same rabbit as in Additional file 1: 1. [file 13028_2015_139_MOESM1_ESM.zip › Brain_2200809/sT1W_3D_TFE_701/IM-0005-0004.jpg]

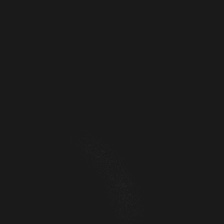

Supplement: Additional file 1: — 1. Complete image series of a transverse TSE T2w sequence in one rabbit. 2. Complete image series of a sagittal TSE T2w sequence in the same rabbit as in Additional file 1: 1. 3. Complete image series of a dorsal TSE T2w sequence in the same rabbit as in Additional file 1: 1. 4. Complete image series of a FLAIR longTR CLEAR sequence in the same rabbit as in Additional file 1: 1. 5. Complete image series of a precontrast T1w 3D (TFE SENSE) sequence in the same rabbit as in Additional file 1: 1. 6. Complete image series of a postcontrast T1w 3D (TFE SENSE) sequence in the same rabbit as in Additional file 1: 1. [file 13028_2015_139_MOESM1_ESM.zip › Brain_2200809/sT1W_3D_TFE_701/IM-0005-0005.jpg]

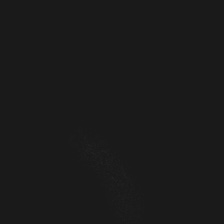

Supplement: Additional file 1: — 1. Complete image series of a transverse TSE T2w sequence in one rabbit. 2. Complete image series of a sagittal TSE T2w sequence in the same rabbit as in Additional file 1: 1. 3. Complete image series of a dorsal TSE T2w sequence in the same rabbit as in Additional file 1: 1. 4. Complete image series of a FLAIR longTR CLEAR sequence in the same rabbit as in Additional file 1: 1. 5. Complete image series of a precontrast T1w 3D (TFE SENSE) sequence in the same rabbit as in Additional file 1: 1. 6. Complete image series of a postcontrast T1w 3D (TFE SENSE) sequence in the same rabbit as in Additional file 1: 1. [file 13028_2015_139_MOESM1_ESM.zip › Brain_2200809/sT1W_3D_TFE_701/IM-0005-0006.jpg]

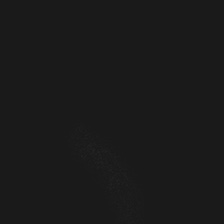

Supplement: Additional file 1: — 1. Complete image series of a transverse TSE T2w sequence in one rabbit. 2. Complete image series of a sagittal TSE T2w sequence in the same rabbit as in Additional file 1: 1. 3. Complete image series of a dorsal TSE T2w sequence in the same rabbit as in Additional file 1: 1. 4. Complete image series of a FLAIR longTR CLEAR sequence in the same rabbit as in Additional file 1: 1. 5. Complete image series of a precontrast T1w 3D (TFE SENSE) sequence in the same rabbit as in Additional file 1: 1. 6. Complete image series of a postcontrast T1w 3D (TFE SENSE) sequence in the same rabbit as in Additional file 1: 1. [file 13028_2015_139_MOESM1_ESM.zip › Brain_2200809/sT1W_3D_TFE_701/IM-0005-0007.jpg]

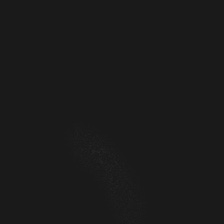

Supplement: Additional file 1: — 1. Complete image series of a transverse TSE T2w sequence in one rabbit. 2. Complete image series of a sagittal TSE T2w sequence in the same rabbit as in Additional file 1: 1. 3. Complete image series of a dorsal TSE T2w sequence in the same rabbit as in Additional file 1: 1. 4. Complete image series of a FLAIR longTR CLEAR sequence in the same rabbit as in Additional file 1: 1. 5. Complete image series of a precontrast T1w 3D (TFE SENSE) sequence in the same rabbit as in Additional file 1: 1. 6. Complete image series of a postcontrast T1w 3D (TFE SENSE) sequence in the same rabbit as in Additional file 1: 1. [file 13028_2015_139_MOESM1_ESM.zip › Brain_2200809/sT1W_3D_TFE_701/IM-0005-0008.jpg]

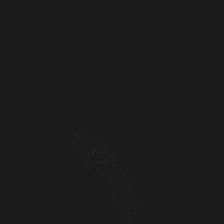

Supplement: Additional file 1: — 1. Complete image series of a transverse TSE T2w sequence in one rabbit. 2. Complete image series of a sagittal TSE T2w sequence in the same rabbit as in Additional file 1: 1. 3. Complete image series of a dorsal TSE T2w sequence in the same rabbit as in Additional file 1: 1. 4. Complete image series of a FLAIR longTR CLEAR sequence in the same rabbit as in Additional file 1: 1. 5. Complete image series of a precontrast T1w 3D (TFE SENSE) sequence in the same rabbit as in Additional file 1: 1. 6. Complete image series of a postcontrast T1w 3D (TFE SENSE) sequence in the same rabbit as in Additional file 1: 1. [file 13028_2015_139_MOESM1_ESM.zip › Brain_2200809/sT1W_3D_TFE_701/IM-0005-0009.jpg]

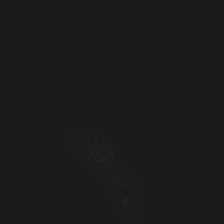

Supplement: Additional file 1: — 1. Complete image series of a transverse TSE T2w sequence in one rabbit. 2. Complete image series of a sagittal TSE T2w sequence in the same rabbit as in Additional file 1: 1. 3. Complete image series of a dorsal TSE T2w sequence in the same rabbit as in Additional file 1: 1. 4. Complete image series of a FLAIR longTR CLEAR sequence in the same rabbit as in Additional file 1: 1. 5. Complete image series of a precontrast T1w 3D (TFE SENSE) sequence in the same rabbit as in Additional file 1: 1. 6. Complete image series of a postcontrast T1w 3D (TFE SENSE) sequence in the same rabbit as in Additional file 1: 1. [file 13028_2015_139_MOESM1_ESM.zip › Brain_2200809/sT1W_3D_TFE_701/IM-0005-0010.jpg]

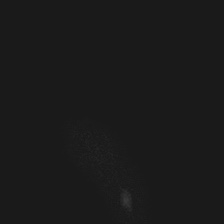

Supplement: Additional file 1: — 1. Complete image series of a transverse TSE T2w sequence in one rabbit. 2. Complete image series of a sagittal TSE T2w sequence in the same rabbit as in Additional file 1: 1. 3. Complete image series of a dorsal TSE T2w sequence in the same rabbit as in Additional file 1: 1. 4. Complete image series of a FLAIR longTR CLEAR sequence in the same rabbit as in Additional file 1: 1. 5. Complete image series of a precontrast T1w 3D (TFE SENSE) sequence in the same rabbit as in Additional file 1: 1. 6. Complete image series of a postcontrast T1w 3D (TFE SENSE) sequence in the same rabbit as in Additional file 1: 1. [file 13028_2015_139_MOESM1_ESM.zip › Brain_2200809/sT1W_3D_TFE_701/IM-0005-0011.jpg]

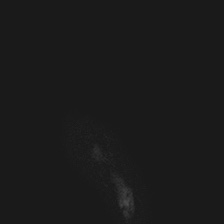

Supplement: Additional file 1: — 1. Complete image series of a transverse TSE T2w sequence in one rabbit. 2. Complete image series of a sagittal TSE T2w sequence in the same rabbit as in Additional file 1: 1. 3. Complete image series of a dorsal TSE T2w sequence in the same rabbit as in Additional file 1: 1. 4. Complete image series of a FLAIR longTR CLEAR sequence in the same rabbit as in Additional file 1: 1. 5. Complete image series of a precontrast T1w 3D (TFE SENSE) sequence in the same rabbit as in Additional file 1: 1. 6. Complete image series of a postcontrast T1w 3D (TFE SENSE) sequence in the same rabbit as in Additional file 1: 1. [file 13028_2015_139_MOESM1_ESM.zip › Brain_2200809/sT1W_3D_TFE_701/IM-0005-0012.jpg]

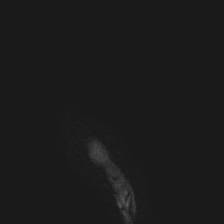

Supplement: Additional file 1: — 1. Complete image series of a transverse TSE T2w sequence in one rabbit. 2. Complete image series of a sagittal TSE T2w sequence in the same rabbit as in Additional file 1: 1. 3. Complete image series of a dorsal TSE T2w sequence in the same rabbit as in Additional file 1: 1. 4. Complete image series of a FLAIR longTR CLEAR sequence in the same rabbit as in Additional file 1: 1. 5. Complete image series of a precontrast T1w 3D (TFE SENSE) sequence in the same rabbit as in Additional file 1: 1. 6. Complete image series of a postcontrast T1w 3D (TFE SENSE) sequence in the same rabbit as in Additional file 1: 1. [file 13028_2015_139_MOESM1_ESM.zip › Brain_2200809/sT1W_3D_TFE_701/IM-0005-0013.jpg]

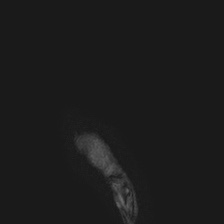

Supplement: Additional file 1: — 1. Complete image series of a transverse TSE T2w sequence in one rabbit. 2. Complete image series of a sagittal TSE T2w sequence in the same rabbit as in Additional file 1: 1. 3. Complete image series of a dorsal TSE T2w sequence in the same rabbit as in Additional file 1: 1. 4. Complete image series of a FLAIR longTR CLEAR sequence in the same rabbit as in Additional file 1: 1. 5. Complete image series of a precontrast T1w 3D (TFE SENSE) sequence in the same rabbit as in Additional file 1: 1. 6. Complete image series of a postcontrast T1w 3D (TFE SENSE) sequence in the same rabbit as in Additional file 1: 1. [file 13028_2015_139_MOESM1_ESM.zip › Brain_2200809/sT1W_3D_TFE_701/IM-0005-0014.jpg]

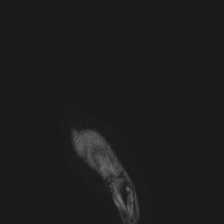

Supplement: Additional file 1: — 1. Complete image series of a transverse TSE T2w sequence in one rabbit. 2. Complete image series of a sagittal TSE T2w sequence in the same rabbit as in Additional file 1: 1. 3. Complete image series of a dorsal TSE T2w sequence in the same rabbit as in Additional file 1: 1. 4. Complete image series of a FLAIR longTR CLEAR sequence in the same rabbit as in Additional file 1: 1. 5. Complete image series of a precontrast T1w 3D (TFE SENSE) sequence in the same rabbit as in Additional file 1: 1. 6. Complete image series of a postcontrast T1w 3D (TFE SENSE) sequence in the same rabbit as in Additional file 1: 1. [file 13028_2015_139_MOESM1_ESM.zip › Brain_2200809/sT1W_3D_TFE_701/IM-0005-0015.jpg]

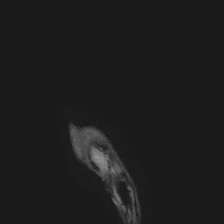

Supplement: Additional file 1: — 1. Complete image series of a transverse TSE T2w sequence in one rabbit. 2. Complete image series of a sagittal TSE T2w sequence in the same rabbit as in Additional file 1: 1. 3. Complete image series of a dorsal TSE T2w sequence in the same rabbit as in Additional file 1: 1. 4. Complete image series of a FLAIR longTR CLEAR sequence in the same rabbit as in Additional file 1: 1. 5. Complete image series of a precontrast T1w 3D (TFE SENSE) sequence in the same rabbit as in Additional file 1: 1. 6. Complete image series of a postcontrast T1w 3D (TFE SENSE) sequence in the same rabbit as in Additional file 1: 1. [file 13028_2015_139_MOESM1_ESM.zip › Brain_2200809/sT1W_3D_TFE_701/IM-0005-0016.jpg]

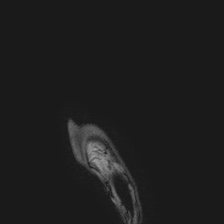

Supplement: Additional file 1: — 1. Complete image series of a transverse TSE T2w sequence in one rabbit. 2. Complete image series of a sagittal TSE T2w sequence in the same rabbit as in Additional file 1: 1. 3. Complete image series of a dorsal TSE T2w sequence in the same rabbit as in Additional file 1: 1. 4. Complete image series of a FLAIR longTR CLEAR sequence in the same rabbit as in Additional file 1: 1. 5. Complete image series of a precontrast T1w 3D (TFE SENSE) sequence in the same rabbit as in Additional file 1: 1. 6. Complete image series of a postcontrast T1w 3D (TFE SENSE) sequence in the same rabbit as in Additional file 1: 1. [file 13028_2015_139_MOESM1_ESM.zip › Brain_2200809/sT1W_3D_TFE_701/IM-0005-0017.jpg]

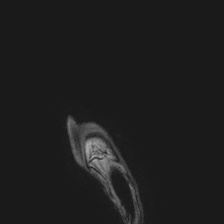

Supplement: Additional file 1: — 1. Complete image series of a transverse TSE T2w sequence in one rabbit. 2. Complete image series of a sagittal TSE T2w sequence in the same rabbit as in Additional file 1: 1. 3. Complete image series of a dorsal TSE T2w sequence in the same rabbit as in Additional file 1: 1. 4. Complete image series of a FLAIR longTR CLEAR sequence in the same rabbit as in Additional file 1: 1. 5. Complete image series of a precontrast T1w 3D (TFE SENSE) sequence in the same rabbit as in Additional file 1: 1. 6. Complete image series of a postcontrast T1w 3D (TFE SENSE) sequence in the same rabbit as in Additional file 1: 1. [file 13028_2015_139_MOESM1_ESM.zip › Brain_2200809/sT1W_3D_TFE_701/IM-0005-0018.jpg]

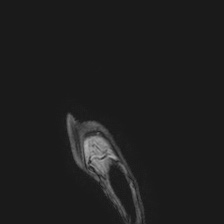

Supplement: Additional file 1: — 1. Complete image series of a transverse TSE T2w sequence in one rabbit. 2. Complete image series of a sagittal TSE T2w sequence in the same rabbit as in Additional file 1: 1. 3. Complete image series of a dorsal TSE T2w sequence in the same rabbit as in Additional file 1: 1. 4. Complete image series of a FLAIR longTR CLEAR sequence in the same rabbit as in Additional file 1: 1. 5. Complete image series of a precontrast T1w 3D (TFE SENSE) sequence in the same rabbit as in Additional file 1: 1. 6. Complete image series of a postcontrast T1w 3D (TFE SENSE) sequence in the same rabbit as in Additional file 1: 1. [file 13028_2015_139_MOESM1_ESM.zip › Brain_2200809/sT1W_3D_TFE_701/IM-0005-0019.jpg]

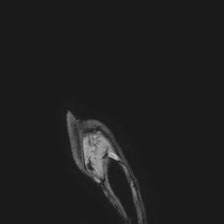

Supplement: Additional file 1: — 1. Complete image series of a transverse TSE T2w sequence in one rabbit. 2. Complete image series of a sagittal TSE T2w sequence in the same rabbit as in Additional file 1: 1. 3. Complete image series of a dorsal TSE T2w sequence in the same rabbit as in Additional file 1: 1. 4. Complete image series of a FLAIR longTR CLEAR sequence in the same rabbit as in Additional file 1: 1. 5. Complete image series of a precontrast T1w 3D (TFE SENSE) sequence in the same rabbit as in Additional file 1: 1. 6. Complete image series of a postcontrast T1w 3D (TFE SENSE) sequence in the same rabbit as in Additional file 1: 1. [file 13028_2015_139_MOESM1_ESM.zip › Brain_2200809/sT1W_3D_TFE_701/IM-0005-0020.jpg]

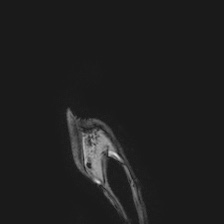

Supplement: Additional file 1: — 1. Complete image series of a transverse TSE T2w sequence in one rabbit. 2. Complete image series of a sagittal TSE T2w sequence in the same rabbit as in Additional file 1: 1. 3. Complete image series of a dorsal TSE T2w sequence in the same rabbit as in Additional file 1: 1. 4. Complete image series of a FLAIR longTR CLEAR sequence in the same rabbit as in Additional file 1: 1. 5. Complete image series of a precontrast T1w 3D (TFE SENSE) sequence in the same rabbit as in Additional file 1: 1. 6. Complete image series of a postcontrast T1w 3D (TFE SENSE) sequence in the same rabbit as in Additional file 1: 1. [file 13028_2015_139_MOESM1_ESM.zip › Brain_2200809/sT1W_3D_TFE_701/IM-0005-0021.jpg]

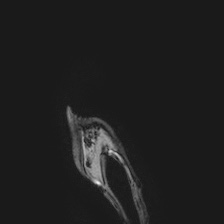

Supplement: Additional file 1: — 1. Complete image series of a transverse TSE T2w sequence in one rabbit. 2. Complete image series of a sagittal TSE T2w sequence in the same rabbit as in Additional file 1: 1. 3. Complete image series of a dorsal TSE T2w sequence in the same rabbit as in Additional file 1: 1. 4. Complete image series of a FLAIR longTR CLEAR sequence in the same rabbit as in Additional file 1: 1. 5. Complete image series of a precontrast T1w 3D (TFE SENSE) sequence in the same rabbit as in Additional file 1: 1. 6. Complete image series of a postcontrast T1w 3D (TFE SENSE) sequence in the same rabbit as in Additional file 1: 1. [file 13028_2015_139_MOESM1_ESM.zip › Brain_2200809/sT1W_3D_TFE_701/IM-0005-0022.jpg]

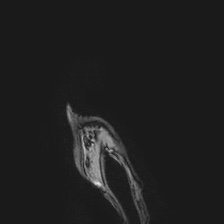

Supplement: Additional file 1: — 1. Complete image series of a transverse TSE T2w sequence in one rabbit. 2. Complete image series of a sagittal TSE T2w sequence in the same rabbit as in Additional file 1: 1. 3. Complete image series of a dorsal TSE T2w sequence in the same rabbit as in Additional file 1: 1. 4. Complete image series of a FLAIR longTR CLEAR sequence in the same rabbit as in Additional file 1: 1. 5. Complete image series of a precontrast T1w 3D (TFE SENSE) sequence in the same rabbit as in Additional file 1: 1. 6. Complete image series of a postcontrast T1w 3D (TFE SENSE) sequence in the same rabbit as in Additional file 1: 1. [file 13028_2015_139_MOESM1_ESM.zip › Brain_2200809/sT1W_3D_TFE_701/IM-0005-0023.jpg]

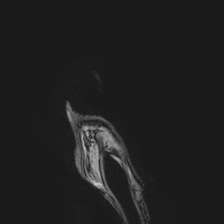

Supplement: Additional file 1: — 1. Complete image series of a transverse TSE T2w sequence in one rabbit. 2. Complete image series of a sagittal TSE T2w sequence in the same rabbit as in Additional file 1: 1. 3. Complete image series of a dorsal TSE T2w sequence in the same rabbit as in Additional file 1: 1. 4. Complete image series of a FLAIR longTR CLEAR sequence in the same rabbit as in Additional file 1: 1. 5. Complete image series of a precontrast T1w 3D (TFE SENSE) sequence in the same rabbit as in Additional file 1: 1. 6. Complete image series of a postcontrast T1w 3D (TFE SENSE) sequence in the same rabbit as in Additional file 1: 1. [file 13028_2015_139_MOESM1_ESM.zip › Brain_2200809/sT1W_3D_TFE_701/IM-0005-0024.jpg]

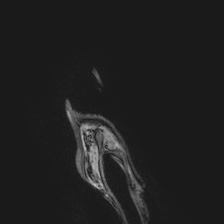

Supplement: Additional file 1: — 1. Complete image series of a transverse TSE T2w sequence in one rabbit. 2. Complete image series of a sagittal TSE T2w sequence in the same rabbit as in Additional file 1: 1. 3. Complete image series of a dorsal TSE T2w sequence in the same rabbit as in Additional file 1: 1. 4. Complete image series of a FLAIR longTR CLEAR sequence in the same rabbit as in Additional file 1: 1. 5. Complete image series of a precontrast T1w 3D (TFE SENSE) sequence in the same rabbit as in Additional file 1: 1. 6. Complete image series of a postcontrast T1w 3D (TFE SENSE) sequence in the same rabbit as in Additional file 1: 1. [file 13028_2015_139_MOESM1_ESM.zip › Brain_2200809/sT1W_3D_TFE_701/IM-0005-0025.jpg]

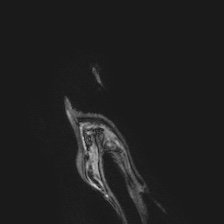

Supplement: Additional file 1: — 1. Complete image series of a transverse TSE T2w sequence in one rabbit. 2. Complete image series of a sagittal TSE T2w sequence in the same rabbit as in Additional file 1: 1. 3. Complete image series of a dorsal TSE T2w sequence in the same rabbit as in Additional file 1: 1. 4. Complete image series of a FLAIR longTR CLEAR sequence in the same rabbit as in Additional file 1: 1. 5. Complete image series of a precontrast T1w 3D (TFE SENSE) sequence in the same rabbit as in Additional file 1: 1. 6. Complete image series of a postcontrast T1w 3D (TFE SENSE) sequence in the same rabbit as in Additional file 1: 1. [file 13028_2015_139_MOESM1_ESM.zip › Brain_2200809/sT1W_3D_TFE_701/IM-0005-0026.jpg]

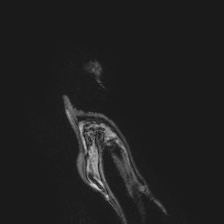

Supplement: Additional file 1: — 1. Complete image series of a transverse TSE T2w sequence in one rabbit. 2. Complete image series of a sagittal TSE T2w sequence in the same rabbit as in Additional file 1: 1. 3. Complete image series of a dorsal TSE T2w sequence in the same rabbit as in Additional file 1: 1. 4. Complete image series of a FLAIR longTR CLEAR sequence in the same rabbit as in Additional file 1: 1. 5. Complete image series of a precontrast T1w 3D (TFE SENSE) sequence in the same rabbit as in Additional file 1: 1. 6. Complete image series of a postcontrast T1w 3D (TFE SENSE) sequence in the same rabbit as in Additional file 1: 1. [file 13028_2015_139_MOESM1_ESM.zip › Brain_2200809/sT1W_3D_TFE_701/IM-0005-0027.jpg]

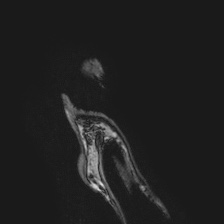

Supplement: Additional file 1: — 1. Complete image series of a transverse TSE T2w sequence in one rabbit. 2. Complete image series of a sagittal TSE T2w sequence in the same rabbit as in Additional file 1: 1. 3. Complete image series of a dorsal TSE T2w sequence in the same rabbit as in Additional file 1: 1. 4. Complete image series of a FLAIR longTR CLEAR sequence in the same rabbit as in Additional file 1: 1. 5. Complete image series of a precontrast T1w 3D (TFE SENSE) sequence in the same rabbit as in Additional file 1: 1. 6. Complete image series of a postcontrast T1w 3D (TFE SENSE) sequence in the same rabbit as in Additional file 1: 1. [file 13028_2015_139_MOESM1_ESM.zip › Brain_2200809/sT1W_3D_TFE_701/IM-0005-0028.jpg]

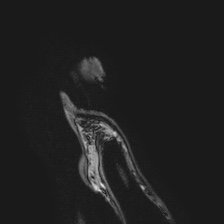

Supplement: Additional file 1: — 1. Complete image series of a transverse TSE T2w sequence in one rabbit. 2. Complete image series of a sagittal TSE T2w sequence in the same rabbit as in Additional file 1: 1. 3. Complete image series of a dorsal TSE T2w sequence in the same rabbit as in Additional file 1: 1. 4. Complete image series of a FLAIR longTR CLEAR sequence in the same rabbit as in Additional file 1: 1. 5. Complete image series of a precontrast T1w 3D (TFE SENSE) sequence in the same rabbit as in Additional file 1: 1. 6. Complete image series of a postcontrast T1w 3D (TFE SENSE) sequence in the same rabbit as in Additional file 1: 1. [file 13028_2015_139_MOESM1_ESM.zip › Brain_2200809/sT1W_3D_TFE_701/IM-0005-0029.jpg]

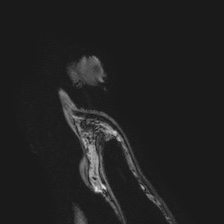

Supplement: Additional file 1: — 1. Complete image series of a transverse TSE T2w sequence in one rabbit. 2. Complete image series of a sagittal TSE T2w sequence in the same rabbit as in Additional file 1: 1. 3. Complete image series of a dorsal TSE T2w sequence in the same rabbit as in Additional file 1: 1. 4. Complete image series of a FLAIR longTR CLEAR sequence in the same rabbit as in Additional file 1: 1. 5. Complete image series of a precontrast T1w 3D (TFE SENSE) sequence in the same rabbit as in Additional file 1: 1. 6. Complete image series of a postcontrast T1w 3D (TFE SENSE) sequence in the same rabbit as in Additional file 1: 1. [file 13028_2015_139_MOESM1_ESM.zip › Brain_2200809/sT1W_3D_TFE_701/IM-0005-0030.jpg]

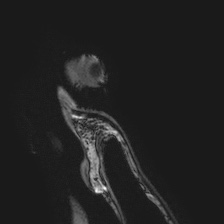

Supplement: Additional file 1: — 1. Complete image series of a transverse TSE T2w sequence in one rabbit. 2. Complete image series of a sagittal TSE T2w sequence in the same rabbit as in Additional file 1: 1. 3. Complete image series of a dorsal TSE T2w sequence in the same rabbit as in Additional file 1: 1. 4. Complete image series of a FLAIR longTR CLEAR sequence in the same rabbit as in Additional file 1: 1. 5. Complete image series of a precontrast T1w 3D (TFE SENSE) sequence in the same rabbit as in Additional file 1: 1. 6. Complete image series of a postcontrast T1w 3D (TFE SENSE) sequence in the same rabbit as in Additional file 1: 1. [file 13028_2015_139_MOESM1_ESM.zip › Brain_2200809/sT1W_3D_TFE_701/IM-0005-0031.jpg]

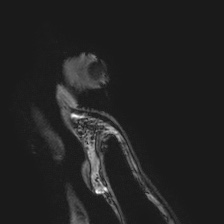

Supplement: Additional file 1: — 1. Complete image series of a transverse TSE T2w sequence in one rabbit. 2. Complete image series of a sagittal TSE T2w sequence in the same rabbit as in Additional file 1: 1. 3. Complete image series of a dorsal TSE T2w sequence in the same rabbit as in Additional file 1: 1. 4. Complete image series of a FLAIR longTR CLEAR sequence in the same rabbit as in Additional file 1: 1. 5. Complete image series of a precontrast T1w 3D (TFE SENSE) sequence in the same rabbit as in Additional file 1: 1. 6. Complete image series of a postcontrast T1w 3D (TFE SENSE) sequence in the same rabbit as in Additional file 1: 1. [file 13028_2015_139_MOESM1_ESM.zip › Brain_2200809/sT1W_3D_TFE_701/IM-0005-0032.jpg]

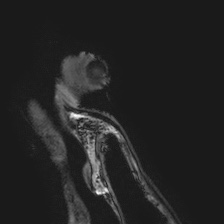

Supplement: Additional file 1: — 1. Complete image series of a transverse TSE T2w sequence in one rabbit. 2. Complete image series of a sagittal TSE T2w sequence in the same rabbit as in Additional file 1: 1. 3. Complete image series of a dorsal TSE T2w sequence in the same rabbit as in Additional file 1: 1. 4. Complete image series of a FLAIR longTR CLEAR sequence in the same rabbit as in Additional file 1: 1. 5. Complete image series of a precontrast T1w 3D (TFE SENSE) sequence in the same rabbit as in Additional file 1: 1. 6. Complete image series of a postcontrast T1w 3D (TFE SENSE) sequence in the same rabbit as in Additional file 1: 1. [file 13028_2015_139_MOESM1_ESM.zip › Brain_2200809/sT1W_3D_TFE_701/IM-0005-0033.jpg]

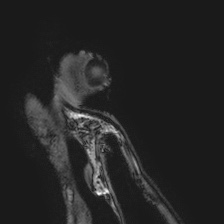

Supplement: Additional file 1: — 1. Complete image series of a transverse TSE T2w sequence in one rabbit. 2. Complete image series of a sagittal TSE T2w sequence in the same rabbit as in Additional file 1: 1. 3. Complete image series of a dorsal TSE T2w sequence in the same rabbit as in Additional file 1: 1. 4. Complete image series of a FLAIR longTR CLEAR sequence in the same rabbit as in Additional file 1: 1. 5. Complete image series of a precontrast T1w 3D (TFE SENSE) sequence in the same rabbit as in Additional file 1: 1. 6. Complete image series of a postcontrast T1w 3D (TFE SENSE) sequence in the same rabbit as in Additional file 1: 1. [file 13028_2015_139_MOESM1_ESM.zip › Brain_2200809/sT1W_3D_TFE_701/IM-0005-0034.jpg]

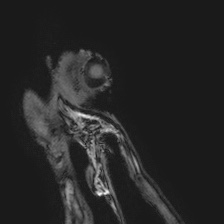

Supplement: Additional file 1: — 1. Complete image series of a transverse TSE T2w sequence in one rabbit. 2. Complete image series of a sagittal TSE T2w sequence in the same rabbit as in Additional file 1: 1. 3. Complete image series of a dorsal TSE T2w sequence in the same rabbit as in Additional file 1: 1. 4. Complete image series of a FLAIR longTR CLEAR sequence in the same rabbit as in Additional file 1: 1. 5. Complete image series of a precontrast T1w 3D (TFE SENSE) sequence in the same rabbit as in Additional file 1: 1. 6. Complete image series of a postcontrast T1w 3D (TFE SENSE) sequence in the same rabbit as in Additional file 1: 1. [file 13028_2015_139_MOESM1_ESM.zip › Brain_2200809/sT1W_3D_TFE_701/IM-0005-0035.jpg]

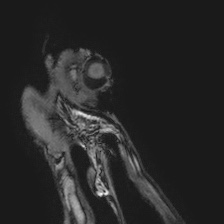

Supplement: Additional file 1: — 1. Complete image series of a transverse TSE T2w sequence in one rabbit. 2. Complete image series of a sagittal TSE T2w sequence in the same rabbit as in Additional file 1: 1. 3. Complete image series of a dorsal TSE T2w sequence in the same rabbit as in Additional file 1: 1. 4. Complete image series of a FLAIR longTR CLEAR sequence in the same rabbit as in Additional file 1: 1. 5. Complete image series of a precontrast T1w 3D (TFE SENSE) sequence in the same rabbit as in Additional file 1: 1. 6. Complete image series of a postcontrast T1w 3D (TFE SENSE) sequence in the same rabbit as in Additional file 1: 1. [file 13028_2015_139_MOESM1_ESM.zip › Brain_2200809/sT1W_3D_TFE_701/IM-0005-0036.jpg]

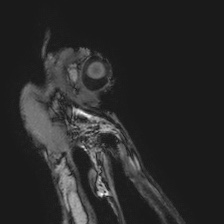

Supplement: Additional file 1: — 1. Complete image series of a transverse TSE T2w sequence in one rabbit. 2. Complete image series of a sagittal TSE T2w sequence in the same rabbit as in Additional file 1: 1. 3. Complete image series of a dorsal TSE T2w sequence in the same rabbit as in Additional file 1: 1. 4. Complete image series of a FLAIR longTR CLEAR sequence in the same rabbit as in Additional file 1: 1. 5. Complete image series of a precontrast T1w 3D (TFE SENSE) sequence in the same rabbit as in Additional file 1: 1. 6. Complete image series of a postcontrast T1w 3D (TFE SENSE) sequence in the same rabbit as in Additional file 1: 1. [file 13028_2015_139_MOESM1_ESM.zip › Brain_2200809/sT1W_3D_TFE_701/IM-0005-0037.jpg]

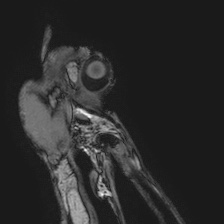

Supplement: Additional file 1: — 1. Complete image series of a transverse TSE T2w sequence in one rabbit. 2. Complete image series of a sagittal TSE T2w sequence in the same rabbit as in Additional file 1: 1. 3. Complete image series of a dorsal TSE T2w sequence in the same rabbit as in Additional file 1: 1. 4. Complete image series of a FLAIR longTR CLEAR sequence in the same rabbit as in Additional file 1: 1. 5. Complete image series of a precontrast T1w 3D (TFE SENSE) sequence in the same rabbit as in Additional file 1: 1. 6. Complete image series of a postcontrast T1w 3D (TFE SENSE) sequence in the same rabbit as in Additional file 1: 1. [file 13028_2015_139_MOESM1_ESM.zip › Brain_2200809/sT1W_3D_TFE_701/IM-0005-0038.jpg]

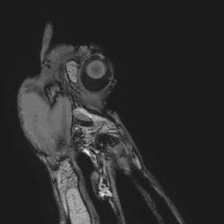

Supplement: Additional file 1: — 1. Complete image series of a transverse TSE T2w sequence in one rabbit. 2. Complete image series of a sagittal TSE T2w sequence in the same rabbit as in Additional file 1: 1. 3. Complete image series of a dorsal TSE T2w sequence in the same rabbit as in Additional file 1: 1. 4. Complete image series of a FLAIR longTR CLEAR sequence in the same rabbit as in Additional file 1: 1. 5. Complete image series of a precontrast T1w 3D (TFE SENSE) sequence in the same rabbit as in Additional file 1: 1. 6. Complete image series of a postcontrast T1w 3D (TFE SENSE) sequence in the same rabbit as in Additional file 1: 1. [file 13028_2015_139_MOESM1_ESM.zip › Brain_2200809/sT1W_3D_TFE_701/IM-0005-0039.jpg]

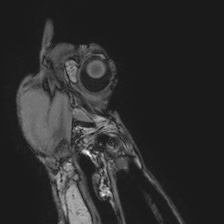

Supplement: Additional file 1: — 1. Complete image series of a transverse TSE T2w sequence in one rabbit. 2. Complete image series of a sagittal TSE T2w sequence in the same rabbit as in Additional file 1: 1. 3. Complete image series of a dorsal TSE T2w sequence in the same rabbit as in Additional file 1: 1. 4. Complete image series of a FLAIR longTR CLEAR sequence in the same rabbit as in Additional file 1: 1. 5. Complete image series of a precontrast T1w 3D (TFE SENSE) sequence in the same rabbit as in Additional file 1: 1. 6. Complete image series of a postcontrast T1w 3D (TFE SENSE) sequence in the same rabbit as in Additional file 1: 1. [file 13028_2015_139_MOESM1_ESM.zip › Brain_2200809/sT1W_3D_TFE_701/IM-0005-0040.jpg]

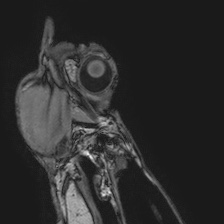

Supplement: Additional file 1: — 1. Complete image series of a transverse TSE T2w sequence in one rabbit. 2. Complete image series of a sagittal TSE T2w sequence in the same rabbit as in Additional file 1: 1. 3. Complete image series of a dorsal TSE T2w sequence in the same rabbit as in Additional file 1: 1. 4. Complete image series of a FLAIR longTR CLEAR sequence in the same rabbit as in Additional file 1: 1. 5. Complete image series of a precontrast T1w 3D (TFE SENSE) sequence in the same rabbit as in Additional file 1: 1. 6. Complete image series of a postcontrast T1w 3D (TFE SENSE) sequence in the same rabbit as in Additional file 1: 1. [file 13028_2015_139_MOESM1_ESM.zip › Brain_2200809/sT1W_3D_TFE_701/IM-0005-0041.jpg]

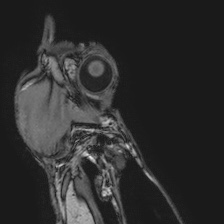

Supplement: Additional file 1: — 1. Complete image series of a transverse TSE T2w sequence in one rabbit. 2. Complete image series of a sagittal TSE T2w sequence in the same rabbit as in Additional file 1: 1. 3. Complete image series of a dorsal TSE T2w sequence in the same rabbit as in Additional file 1: 1. 4. Complete image series of a FLAIR longTR CLEAR sequence in the same rabbit as in Additional file 1: 1. 5. Complete image series of a precontrast T1w 3D (TFE SENSE) sequence in the same rabbit as in Additional file 1: 1. 6. Complete image series of a postcontrast T1w 3D (TFE SENSE) sequence in the same rabbit as in Additional file 1: 1. [file 13028_2015_139_MOESM1_ESM.zip › Brain_2200809/sT1W_3D_TFE_701/IM-0005-0042.jpg]

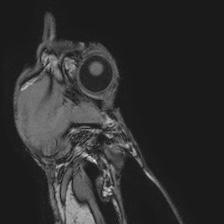

Supplement: Additional file 1: — 1. Complete image series of a transverse TSE T2w sequence in one rabbit. 2. Complete image series of a sagittal TSE T2w sequence in the same rabbit as in Additional file 1: 1. 3. Complete image series of a dorsal TSE T2w sequence in the same rabbit as in Additional file 1: 1. 4. Complete image series of a FLAIR longTR CLEAR sequence in the same rabbit as in Additional file 1: 1. 5. Complete image series of a precontrast T1w 3D (TFE SENSE) sequence in the same rabbit as in Additional file 1: 1. 6. Complete image series of a postcontrast T1w 3D (TFE SENSE) sequence in the same rabbit as in Additional file 1: 1. [file 13028_2015_139_MOESM1_ESM.zip › Brain_2200809/sT1W_3D_TFE_701/IM-0005-0043.jpg]

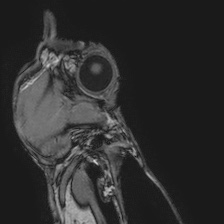

Supplement: Additional file 1: — 1. Complete image series of a transverse TSE T2w sequence in one rabbit. 2. Complete image series of a sagittal TSE T2w sequence in the same rabbit as in Additional file 1: 1. 3. Complete image series of a dorsal TSE T2w sequence in the same rabbit as in Additional file 1: 1. 4. Complete image series of a FLAIR longTR CLEAR sequence in the same rabbit as in Additional file 1: 1. 5. Complete image series of a precontrast T1w 3D (TFE SENSE) sequence in the same rabbit as in Additional file 1: 1. 6. Complete image series of a postcontrast T1w 3D (TFE SENSE) sequence in the same rabbit as in Additional file 1: 1. [file 13028_2015_139_MOESM1_ESM.zip › Brain_2200809/sT1W_3D_TFE_701/IM-0005-0044.jpg]

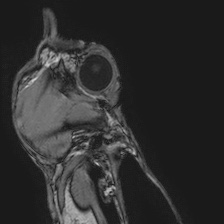

Supplement: Additional file 1: — 1. Complete image series of a transverse TSE T2w sequence in one rabbit. 2. Complete image series of a sagittal TSE T2w sequence in the same rabbit as in Additional file 1: 1. 3. Complete image series of a dorsal TSE T2w sequence in the same rabbit as in Additional file 1: 1. 4. Complete image series of a FLAIR longTR CLEAR sequence in the same rabbit as in Additional file 1: 1. 5. Complete image series of a precontrast T1w 3D (TFE SENSE) sequence in the same rabbit as in Additional file 1: 1. 6. Complete image series of a postcontrast T1w 3D (TFE SENSE) sequence in the same rabbit as in Additional file 1: 1. [file 13028_2015_139_MOESM1_ESM.zip › Brain_2200809/sT1W_3D_TFE_701/IM-0005-0045.jpg]

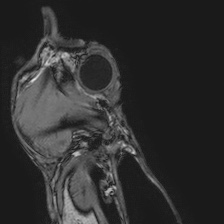

Supplement: Additional file 1: — 1. Complete image series of a transverse TSE T2w sequence in one rabbit. 2. Complete image series of a sagittal TSE T2w sequence in the same rabbit as in Additional file 1: 1. 3. Complete image series of a dorsal TSE T2w sequence in the same rabbit as in Additional file 1: 1. 4. Complete image series of a FLAIR longTR CLEAR sequence in the same rabbit as in Additional file 1: 1. 5. Complete image series of a precontrast T1w 3D (TFE SENSE) sequence in the same rabbit as in Additional file 1: 1. 6. Complete image series of a postcontrast T1w 3D (TFE SENSE) sequence in the same rabbit as in Additional file 1: 1. [file 13028_2015_139_MOESM1_ESM.zip › Brain_2200809/sT1W_3D_TFE_701/IM-0005-0046.jpg]

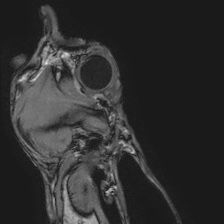

Supplement: Additional file 1: — 1. Complete image series of a transverse TSE T2w sequence in one rabbit. 2. Complete image series of a sagittal TSE T2w sequence in the same rabbit as in Additional file 1: 1. 3. Complete image series of a dorsal TSE T2w sequence in the same rabbit as in Additional file 1: 1. 4. Complete image series of a FLAIR longTR CLEAR sequence in the same rabbit as in Additional file 1: 1. 5. Complete image series of a precontrast T1w 3D (TFE SENSE) sequence in the same rabbit as in Additional file 1: 1. 6. Complete image series of a postcontrast T1w 3D (TFE SENSE) sequence in the same rabbit as in Additional file 1: 1. [file 13028_2015_139_MOESM1_ESM.zip › Brain_2200809/sT1W_3D_TFE_701/IM-0005-0047.jpg]

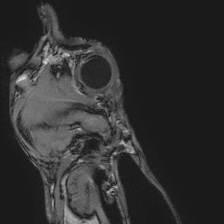

Supplement: Additional file 1: — 1. Complete image series of a transverse TSE T2w sequence in one rabbit. 2. Complete image series of a sagittal TSE T2w sequence in the same rabbit as in Additional file 1: 1. 3. Complete image series of a dorsal TSE T2w sequence in the same rabbit as in Additional file 1: 1. 4. Complete image series of a FLAIR longTR CLEAR sequence in the same rabbit as in Additional file 1: 1. 5. Complete image series of a precontrast T1w 3D (TFE SENSE) sequence in the same rabbit as in Additional file 1: 1. 6. Complete image series of a postcontrast T1w 3D (TFE SENSE) sequence in the same rabbit as in Additional file 1: 1. [file 13028_2015_139_MOESM1_ESM.zip › Brain_2200809/sT1W_3D_TFE_701/IM-0005-0048.jpg]

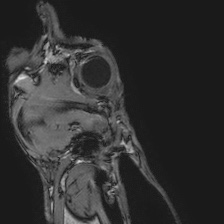

Supplement: Additional file 1: — 1. Complete image series of a transverse TSE T2w sequence in one rabbit. 2. Complete image series of a sagittal TSE T2w sequence in the same rabbit as in Additional file 1: 1. 3. Complete image series of a dorsal TSE T2w sequence in the same rabbit as in Additional file 1: 1. 4. Complete image series of a FLAIR longTR CLEAR sequence in the same rabbit as in Additional file 1: 1. 5. Complete image series of a precontrast T1w 3D (TFE SENSE) sequence in the same rabbit as in Additional file 1: 1. 6. Complete image series of a postcontrast T1w 3D (TFE SENSE) sequence in the same rabbit as in Additional file 1: 1. [file 13028_2015_139_MOESM1_ESM.zip › Brain_2200809/sT1W_3D_TFE_701/IM-0005-0049.jpg]

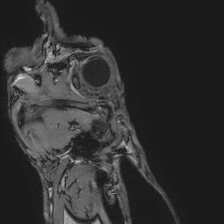

Supplement: Additional file 1: — 1. Complete image series of a transverse TSE T2w sequence in one rabbit. 2. Complete image series of a sagittal TSE T2w sequence in the same rabbit as in Additional file 1: 1. 3. Complete image series of a dorsal TSE T2w sequence in the same rabbit as in Additional file 1: 1. 4. Complete image series of a FLAIR longTR CLEAR sequence in the same rabbit as in Additional file 1: 1. 5. Complete image series of a precontrast T1w 3D (TFE SENSE) sequence in the same rabbit as in Additional file 1: 1. 6. Complete image series of a postcontrast T1w 3D (TFE SENSE) sequence in the same rabbit as in Additional file 1: 1. [file 13028_2015_139_MOESM1_ESM.zip › Brain_2200809/sT1W_3D_TFE_701/IM-0005-0050.jpg]

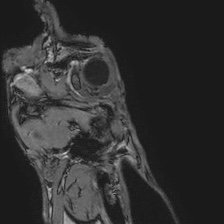

Supplement: Additional file 1: — 1. Complete image series of a transverse TSE T2w sequence in one rabbit. 2. Complete image series of a sagittal TSE T2w sequence in the same rabbit as in Additional file 1: 1. 3. Complete image series of a dorsal TSE T2w sequence in the same rabbit as in Additional file 1: 1. 4. Complete image series of a FLAIR longTR CLEAR sequence in the same rabbit as in Additional file 1: 1. 5. Complete image series of a precontrast T1w 3D (TFE SENSE) sequence in the same rabbit as in Additional file 1: 1. 6. Complete image series of a postcontrast T1w 3D (TFE SENSE) sequence in the same rabbit as in Additional file 1: 1. [file 13028_2015_139_MOESM1_ESM.zip › Brain_2200809/sT1W_3D_TFE_701/IM-0005-0051.jpg]

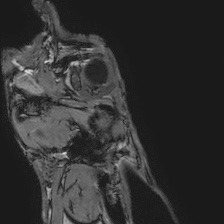

Supplement: Additional file 1: — 1. Complete image series of a transverse TSE T2w sequence in one rabbit. 2. Complete image series of a sagittal TSE T2w sequence in the same rabbit as in Additional file 1: 1. 3. Complete image series of a dorsal TSE T2w sequence in the same rabbit as in Additional file 1: 1. 4. Complete image series of a FLAIR longTR CLEAR sequence in the same rabbit as in Additional file 1: 1. 5. Complete image series of a precontrast T1w 3D (TFE SENSE) sequence in the same rabbit as in Additional file 1: 1. 6. Complete image series of a postcontrast T1w 3D (TFE SENSE) sequence in the same rabbit as in Additional file 1: 1. [file 13028_2015_139_MOESM1_ESM.zip › Brain_2200809/sT1W_3D_TFE_701/IM-0005-0052.jpg]

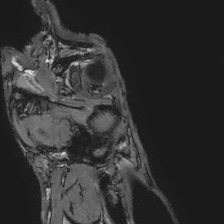

Supplement: Additional file 1: — 1. Complete image series of a transverse TSE T2w sequence in one rabbit. 2. Complete image series of a sagittal TSE T2w sequence in the same rabbit as in Additional file 1: 1. 3. Complete image series of a dorsal TSE T2w sequence in the same rabbit as in Additional file 1: 1. 4. Complete image series of a FLAIR longTR CLEAR sequence in the same rabbit as in Additional file 1: 1. 5. Complete image series of a precontrast T1w 3D (TFE SENSE) sequence in the same rabbit as in Additional file 1: 1. 6. Complete image series of a postcontrast T1w 3D (TFE SENSE) sequence in the same rabbit as in Additional file 1: 1. [file 13028_2015_139_MOESM1_ESM.zip › Brain_2200809/sT1W_3D_TFE_701/IM-0005-0053.jpg]

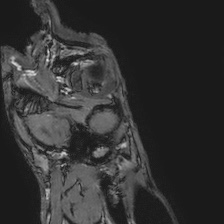

Supplement: Additional file 1: — 1. Complete image series of a transverse TSE T2w sequence in one rabbit. 2. Complete image series of a sagittal TSE T2w sequence in the same rabbit as in Additional file 1: 1. 3. Complete image series of a dorsal TSE T2w sequence in the same rabbit as in Additional file 1: 1. 4. Complete image series of a FLAIR longTR CLEAR sequence in the same rabbit as in Additional file 1: 1. 5. Complete image series of a precontrast T1w 3D (TFE SENSE) sequence in the same rabbit as in Additional file 1: 1. 6. Complete image series of a postcontrast T1w 3D (TFE SENSE) sequence in the same rabbit as in Additional file 1: 1. [file 13028_2015_139_MOESM1_ESM.zip › Brain_2200809/sT1W_3D_TFE_701/IM-0005-0054.jpg]

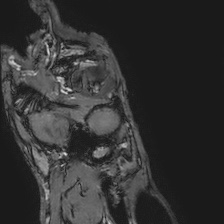

Supplement: Additional file 1: — 1. Complete image series of a transverse TSE T2w sequence in one rabbit. 2. Complete image series of a sagittal TSE T2w sequence in the same rabbit as in Additional file 1: 1. 3. Complete image series of a dorsal TSE T2w sequence in the same rabbit as in Additional file 1: 1. 4. Complete image series of a FLAIR longTR CLEAR sequence in the same rabbit as in Additional file 1: 1. 5. Complete image series of a precontrast T1w 3D (TFE SENSE) sequence in the same rabbit as in Additional file 1: 1. 6. Complete image series of a postcontrast T1w 3D (TFE SENSE) sequence in the same rabbit as in Additional file 1: 1. [file 13028_2015_139_MOESM1_ESM.zip › Brain_2200809/sT1W_3D_TFE_701/IM-0005-0055.jpg]

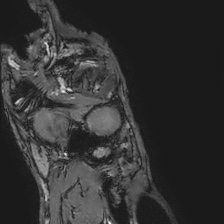

Supplement: Additional file 1: — 1. Complete image series of a transverse TSE T2w sequence in one rabbit. 2. Complete image series of a sagittal TSE T2w sequence in the same rabbit as in Additional file 1: 1. 3. Complete image series of a dorsal TSE T2w sequence in the same rabbit as in Additional file 1: 1. 4. Complete image series of a FLAIR longTR CLEAR sequence in the same rabbit as in Additional file 1: 1. 5. Complete image series of a precontrast T1w 3D (TFE SENSE) sequence in the same rabbit as in Additional file 1: 1. 6. Complete image series of a postcontrast T1w 3D (TFE SENSE) sequence in the same rabbit as in Additional file 1: 1. [file 13028_2015_139_MOESM1_ESM.zip › Brain_2200809/sT1W_3D_TFE_701/IM-0005-0056.jpg]

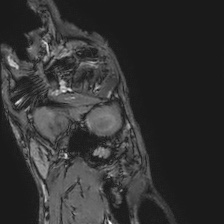

Supplement: Additional file 1: — 1. Complete image series of a transverse TSE T2w sequence in one rabbit. 2. Complete image series of a sagittal TSE T2w sequence in the same rabbit as in Additional file 1: 1. 3. Complete image series of a dorsal TSE T2w sequence in the same rabbit as in Additional file 1: 1. 4. Complete image series of a FLAIR longTR CLEAR sequence in the same rabbit as in Additional file 1: 1. 5. Complete image series of a precontrast T1w 3D (TFE SENSE) sequence in the same rabbit as in Additional file 1: 1. 6. Complete image series of a postcontrast T1w 3D (TFE SENSE) sequence in the same rabbit as in Additional file 1: 1. [file 13028_2015_139_MOESM1_ESM.zip › Brain_2200809/sT1W_3D_TFE_701/IM-0005-0057.jpg]

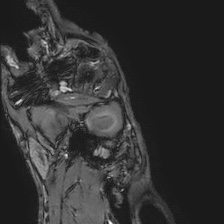

Supplement: Additional file 1: — 1. Complete image series of a transverse TSE T2w sequence in one rabbit. 2. Complete image series of a sagittal TSE T2w sequence in the same rabbit as in Additional file 1: 1. 3. Complete image series of a dorsal TSE T2w sequence in the same rabbit as in Additional file 1: 1. 4. Complete image series of a FLAIR longTR CLEAR sequence in the same rabbit as in Additional file 1: 1. 5. Complete image series of a precontrast T1w 3D (TFE SENSE) sequence in the same rabbit as in Additional file 1: 1. 6. Complete image series of a postcontrast T1w 3D (TFE SENSE) sequence in the same rabbit as in Additional file 1: 1. [file 13028_2015_139_MOESM1_ESM.zip › Brain_2200809/sT1W_3D_TFE_701/IM-0005-0058.jpg]

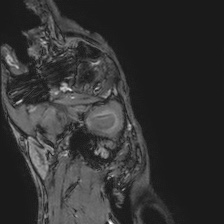

Supplement: Additional file 1: — 1. Complete image series of a transverse TSE T2w sequence in one rabbit. 2. Complete image series of a sagittal TSE T2w sequence in the same rabbit as in Additional file 1: 1. 3. Complete image series of a dorsal TSE T2w sequence in the same rabbit as in Additional file 1: 1. 4. Complete image series of a FLAIR longTR CLEAR sequence in the same rabbit as in Additional file 1: 1. 5. Complete image series of a precontrast T1w 3D (TFE SENSE) sequence in the same rabbit as in Additional file 1: 1. 6. Complete image series of a postcontrast T1w 3D (TFE SENSE) sequence in the same rabbit as in Additional file 1: 1. [file 13028_2015_139_MOESM1_ESM.zip › Brain_2200809/sT1W_3D_TFE_701/IM-0005-0059.jpg]

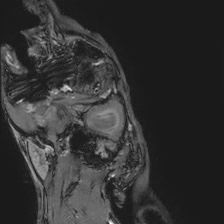

Supplement: Additional file 1: — 1. Complete image series of a transverse TSE T2w sequence in one rabbit. 2. Complete image series of a sagittal TSE T2w sequence in the same rabbit as in Additional file 1: 1. 3. Complete image series of a dorsal TSE T2w sequence in the same rabbit as in Additional file 1: 1. 4. Complete image series of a FLAIR longTR CLEAR sequence in the same rabbit as in Additional file 1: 1. 5. Complete image series of a precontrast T1w 3D (TFE SENSE) sequence in the same rabbit as in Additional file 1: 1. 6. Complete image series of a postcontrast T1w 3D (TFE SENSE) sequence in the same rabbit as in Additional file 1: 1. [file 13028_2015_139_MOESM1_ESM.zip › Brain_2200809/sT1W_3D_TFE_701/IM-0005-0060.jpg]

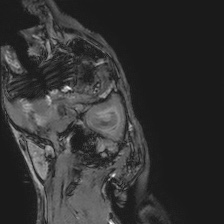

Supplement: Additional file 1: — 1. Complete image series of a transverse TSE T2w sequence in one rabbit. 2. Complete image series of a sagittal TSE T2w sequence in the same rabbit as in Additional file 1: 1. 3. Complete image series of a dorsal TSE T2w sequence in the same rabbit as in Additional file 1: 1. 4. Complete image series of a FLAIR longTR CLEAR sequence in the same rabbit as in Additional file 1: 1. 5. Complete image series of a precontrast T1w 3D (TFE SENSE) sequence in the same rabbit as in Additional file 1: 1. 6. Complete image series of a postcontrast T1w 3D (TFE SENSE) sequence in the same rabbit as in Additional file 1: 1. [file 13028_2015_139_MOESM1_ESM.zip › Brain_2200809/sT1W_3D_TFE_701/IM-0005-0061.jpg]

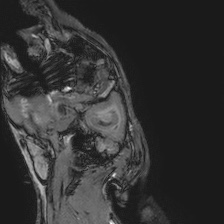

Supplement: Additional file 1: — 1. Complete image series of a transverse TSE T2w sequence in one rabbit. 2. Complete image series of a sagittal TSE T2w sequence in the same rabbit as in Additional file 1: 1. 3. Complete image series of a dorsal TSE T2w sequence in the same rabbit as in Additional file 1: 1. 4. Complete image series of a FLAIR longTR CLEAR sequence in the same rabbit as in Additional file 1: 1. 5. Complete image series of a precontrast T1w 3D (TFE SENSE) sequence in the same rabbit as in Additional file 1: 1. 6. Complete image series of a postcontrast T1w 3D (TFE SENSE) sequence in the same rabbit as in Additional file 1: 1. [file 13028_2015_139_MOESM1_ESM.zip › Brain_2200809/sT1W_3D_TFE_701/IM-0005-0062.jpg]

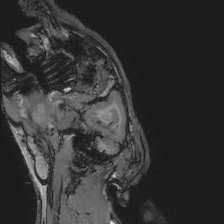

Supplement: Additional file 1: — 1. Complete image series of a transverse TSE T2w sequence in one rabbit. 2. Complete image series of a sagittal TSE T2w sequence in the same rabbit as in Additional file 1: 1. 3. Complete image series of a dorsal TSE T2w sequence in the same rabbit as in Additional file 1: 1. 4. Complete image series of a FLAIR longTR CLEAR sequence in the same rabbit as in Additional file 1: 1. 5. Complete image series of a precontrast T1w 3D (TFE SENSE) sequence in the same rabbit as in Additional file 1: 1. 6. Complete image series of a postcontrast T1w 3D (TFE SENSE) sequence in the same rabbit as in Additional file 1: 1. [file 13028_2015_139_MOESM1_ESM.zip › Brain_2200809/sT1W_3D_TFE_701/IM-0005-0063.jpg]

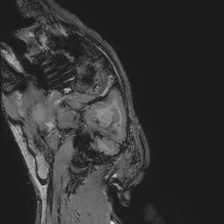

Supplement: Additional file 1: — 1. Complete image series of a transverse TSE T2w sequence in one rabbit. 2. Complete image series of a sagittal TSE T2w sequence in the same rabbit as in Additional file 1: 1. 3. Complete image series of a dorsal TSE T2w sequence in the same rabbit as in Additional file 1: 1. 4. Complete image series of a FLAIR longTR CLEAR sequence in the same rabbit as in Additional file 1: 1. 5. Complete image series of a precontrast T1w 3D (TFE SENSE) sequence in the same rabbit as in Additional file 1: 1. 6. Complete image series of a postcontrast T1w 3D (TFE SENSE) sequence in the same rabbit as in Additional file 1: 1. [file 13028_2015_139_MOESM1_ESM.zip › Brain_2200809/sT1W_3D_TFE_701/IM-0005-0064.jpg]

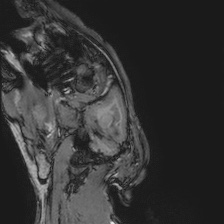

Supplement: Additional file 1: — 1. Complete image series of a transverse TSE T2w sequence in one rabbit. 2. Complete image series of a sagittal TSE T2w sequence in the same rabbit as in Additional file 1: 1. 3. Complete image series of a dorsal TSE T2w sequence in the same rabbit as in Additional file 1: 1. 4. Complete image series of a FLAIR longTR CLEAR sequence in the same rabbit as in Additional file 1: 1. 5. Complete image series of a precontrast T1w 3D (TFE SENSE) sequence in the same rabbit as in Additional file 1: 1. 6. Complete image series of a postcontrast T1w 3D (TFE SENSE) sequence in the same rabbit as in Additional file 1: 1. [file 13028_2015_139_MOESM1_ESM.zip › Brain_2200809/sT1W_3D_TFE_701/IM-0005-0065.jpg]

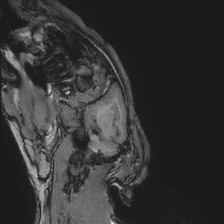

Supplement: Additional file 1: — 1. Complete image series of a transverse TSE T2w sequence in one rabbit. 2. Complete image series of a sagittal TSE T2w sequence in the same rabbit as in Additional file 1: 1. 3. Complete image series of a dorsal TSE T2w sequence in the same rabbit as in Additional file 1: 1. 4. Complete image series of a FLAIR longTR CLEAR sequence in the same rabbit as in Additional file 1: 1. 5. Complete image series of a precontrast T1w 3D (TFE SENSE) sequence in the same rabbit as in Additional file 1: 1. 6. Complete image series of a postcontrast T1w 3D (TFE SENSE) sequence in the same rabbit as in Additional file 1: 1. [file 13028_2015_139_MOESM1_ESM.zip › Brain_2200809/sT1W_3D_TFE_701/IM-0005-0066.jpg]

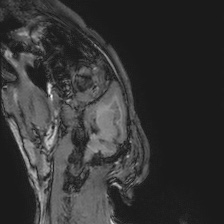

Supplement: Additional file 1: — 1. Complete image series of a transverse TSE T2w sequence in one rabbit. 2. Complete image series of a sagittal TSE T2w sequence in the same rabbit as in Additional file 1: 1. 3. Complete image series of a dorsal TSE T2w sequence in the same rabbit as in Additional file 1: 1. 4. Complete image series of a FLAIR longTR CLEAR sequence in the same rabbit as in Additional file 1: 1. 5. Complete image series of a precontrast T1w 3D (TFE SENSE) sequence in the same rabbit as in Additional file 1: 1. 6. Complete image series of a postcontrast T1w 3D (TFE SENSE) sequence in the same rabbit as in Additional file 1: 1. [file 13028_2015_139_MOESM1_ESM.zip › Brain_2200809/sT1W_3D_TFE_701/IM-0005-0067.jpg]

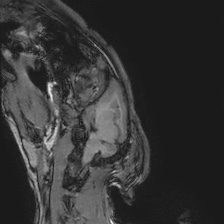

Supplement: Additional file 1: — 1. Complete image series of a transverse TSE T2w sequence in one rabbit. 2. Complete image series of a sagittal TSE T2w sequence in the same rabbit as in Additional file 1: 1. 3. Complete image series of a dorsal TSE T2w sequence in the same rabbit as in Additional file 1: 1. 4. Complete image series of a FLAIR longTR CLEAR sequence in the same rabbit as in Additional file 1: 1. 5. Complete image series of a precontrast T1w 3D (TFE SENSE) sequence in the same rabbit as in Additional file 1: 1. 6. Complete image series of a postcontrast T1w 3D (TFE SENSE) sequence in the same rabbit as in Additional file 1: 1. [file 13028_2015_139_MOESM1_ESM.zip › Brain_2200809/sT1W_3D_TFE_701/IM-0005-0068.jpg]

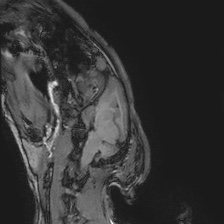

Supplement: Additional file 1: — 1. Complete image series of a transverse TSE T2w sequence in one rabbit. 2. Complete image series of a sagittal TSE T2w sequence in the same rabbit as in Additional file 1: 1. 3. Complete image series of a dorsal TSE T2w sequence in the same rabbit as in Additional file 1: 1. 4. Complete image series of a FLAIR longTR CLEAR sequence in the same rabbit as in Additional file 1: 1. 5. Complete image series of a precontrast T1w 3D (TFE SENSE) sequence in the same rabbit as in Additional file 1: 1. 6. Complete image series of a postcontrast T1w 3D (TFE SENSE) sequence in the same rabbit as in Additional file 1: 1. [file 13028_2015_139_MOESM1_ESM.zip › Brain_2200809/sT1W_3D_TFE_701/IM-0005-0069.jpg]

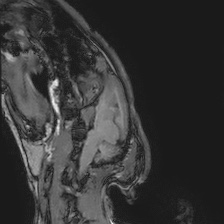

Supplement: Additional file 1: — 1. Complete image series of a transverse TSE T2w sequence in one rabbit. 2. Complete image series of a sagittal TSE T2w sequence in the same rabbit as in Additional file 1: 1. 3. Complete image series of a dorsal TSE T2w sequence in the same rabbit as in Additional file 1: 1. 4. Complete image series of a FLAIR longTR CLEAR sequence in the same rabbit as in Additional file 1: 1. 5. Complete image series of a precontrast T1w 3D (TFE SENSE) sequence in the same rabbit as in Additional file 1: 1. 6. Complete image series of a postcontrast T1w 3D (TFE SENSE) sequence in the same rabbit as in Additional file 1: 1. [file 13028_2015_139_MOESM1_ESM.zip › Brain_2200809/sT1W_3D_TFE_701/IM-0005-0070.jpg]

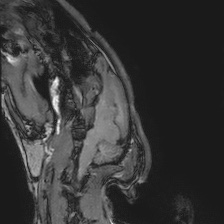

Supplement: Additional file 1: — 1. Complete image series of a transverse TSE T2w sequence in one rabbit. 2. Complete image series of a sagittal TSE T2w sequence in the same rabbit as in Additional file 1: 1. 3. Complete image series of a dorsal TSE T2w sequence in the same rabbit as in Additional file 1: 1. 4. Complete image series of a FLAIR longTR CLEAR sequence in the same rabbit as in Additional file 1: 1. 5. Complete image series of a precontrast T1w 3D (TFE SENSE) sequence in the same rabbit as in Additional file 1: 1. 6. Complete image series of a postcontrast T1w 3D (TFE SENSE) sequence in the same rabbit as in Additional file 1: 1. [file 13028_2015_139_MOESM1_ESM.zip › Brain_2200809/sT1W_3D_TFE_701/IM-0005-0071.jpg]

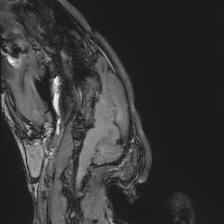

Supplement: Additional file 1: — 1. Complete image series of a transverse TSE T2w sequence in one rabbit. 2. Complete image series of a sagittal TSE T2w sequence in the same rabbit as in Additional file 1: 1. 3. Complete image series of a dorsal TSE T2w sequence in the same rabbit as in Additional file 1: 1. 4. Complete image series of a FLAIR longTR CLEAR sequence in the same rabbit as in Additional file 1: 1. 5. Complete image series of a precontrast T1w 3D (TFE SENSE) sequence in the same rabbit as in Additional file 1: 1. 6. Complete image series of a postcontrast T1w 3D (TFE SENSE) sequence in the same rabbit as in Additional file 1: 1. [file 13028_2015_139_MOESM1_ESM.zip › Brain_2200809/sT1W_3D_TFE_701/IM-0005-0072.jpg]

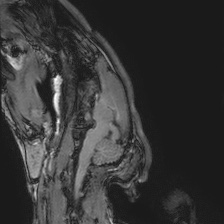

Supplement: Additional file 1: — 1. Complete image series of a transverse TSE T2w sequence in one rabbit. 2. Complete image series of a sagittal TSE T2w sequence in the same rabbit as in Additional file 1: 1. 3. Complete image series of a dorsal TSE T2w sequence in the same rabbit as in Additional file 1: 1. 4. Complete image series of a FLAIR longTR CLEAR sequence in the same rabbit as in Additional file 1: 1. 5. Complete image series of a precontrast T1w 3D (TFE SENSE) sequence in the same rabbit as in Additional file 1: 1. 6. Complete image series of a postcontrast T1w 3D (TFE SENSE) sequence in the same rabbit as in Additional file 1: 1. [file 13028_2015_139_MOESM1_ESM.zip › Brain_2200809/sT1W_3D_TFE_701/IM-0005-0073.jpg]

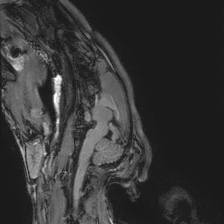

Supplement: Additional file 1: — 1. Complete image series of a transverse TSE T2w sequence in one rabbit. 2. Complete image series of a sagittal TSE T2w sequence in the same rabbit as in Additional file 1: 1. 3. Complete image series of a dorsal TSE T2w sequence in the same rabbit as in Additional file 1: 1. 4. Complete image series of a FLAIR longTR CLEAR sequence in the same rabbit as in Additional file 1: 1. 5. Complete image series of a precontrast T1w 3D (TFE SENSE) sequence in the same rabbit as in Additional file 1: 1. 6. Complete image series of a postcontrast T1w 3D (TFE SENSE) sequence in the same rabbit as in Additional file 1: 1. [file 13028_2015_139_MOESM1_ESM.zip › Brain_2200809/sT1W_3D_TFE_701/IM-0005-0074.jpg]

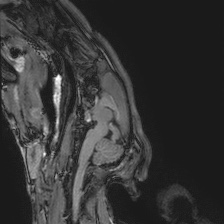

Supplement: Additional file 1: — 1. Complete image series of a transverse TSE T2w sequence in one rabbit. 2. Complete image series of a sagittal TSE T2w sequence in the same rabbit as in Additional file 1: 1. 3. Complete image series of a dorsal TSE T2w sequence in the same rabbit as in Additional file 1: 1. 4. Complete image series of a FLAIR longTR CLEAR sequence in the same rabbit as in Additional file 1: 1. 5. Complete image series of a precontrast T1w 3D (TFE SENSE) sequence in the same rabbit as in Additional file 1: 1. 6. Complete image series of a postcontrast T1w 3D (TFE SENSE) sequence in the same rabbit as in Additional file 1: 1. [file 13028_2015_139_MOESM1_ESM.zip › Brain_2200809/sT1W_3D_TFE_701/IM-0005-0075.jpg]

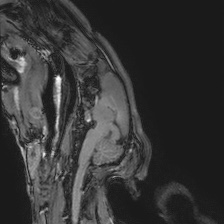

Supplement: Additional file 1: — 1. Complete image series of a transverse TSE T2w sequence in one rabbit. 2. Complete image series of a sagittal TSE T2w sequence in the same rabbit as in Additional file 1: 1. 3. Complete image series of a dorsal TSE T2w sequence in the same rabbit as in Additional file 1: 1. 4. Complete image series of a FLAIR longTR CLEAR sequence in the same rabbit as in Additional file 1: 1. 5. Complete image series of a precontrast T1w 3D (TFE SENSE) sequence in the same rabbit as in Additional file 1: 1. 6. Complete image series of a postcontrast T1w 3D (TFE SENSE) sequence in the same rabbit as in Additional file 1: 1. [file 13028_2015_139_MOESM1_ESM.zip › Brain_2200809/sT1W_3D_TFE_701/IM-0005-0076.jpg]

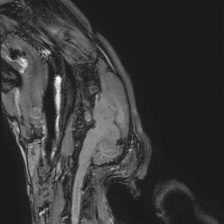

Supplement: Additional file 1: — 1. Complete image series of a transverse TSE T2w sequence in one rabbit. 2. Complete image series of a sagittal TSE T2w sequence in the same rabbit as in Additional file 1: 1. 3. Complete image series of a dorsal TSE T2w sequence in the same rabbit as in Additional file 1: 1. 4. Complete image series of a FLAIR longTR CLEAR sequence in the same rabbit as in Additional file 1: 1. 5. Complete image series of a precontrast T1w 3D (TFE SENSE) sequence in the same rabbit as in Additional file 1: 1. 6. Complete image series of a postcontrast T1w 3D (TFE SENSE) sequence in the same rabbit as in Additional file 1: 1. [file 13028_2015_139_MOESM1_ESM.zip › Brain_2200809/sT1W_3D_TFE_701/IM-0005-0077.jpg]

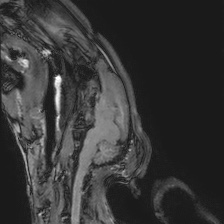

Supplement: Additional file 1: — 1. Complete image series of a transverse TSE T2w sequence in one rabbit. 2. Complete image series of a sagittal TSE T2w sequence in the same rabbit as in Additional file 1: 1. 3. Complete image series of a dorsal TSE T2w sequence in the same rabbit as in Additional file 1: 1. 4. Complete image series of a FLAIR longTR CLEAR sequence in the same rabbit as in Additional file 1: 1. 5. Complete image series of a precontrast T1w 3D (TFE SENSE) sequence in the same rabbit as in Additional file 1: 1. 6. Complete image series of a postcontrast T1w 3D (TFE SENSE) sequence in the same rabbit as in Additional file 1: 1. [file 13028_2015_139_MOESM1_ESM.zip › Brain_2200809/sT1W_3D_TFE_701/IM-0005-0078.jpg]

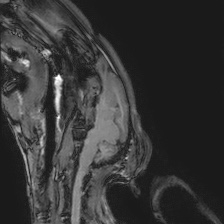

Supplement: Additional file 1: — 1. Complete image series of a transverse TSE T2w sequence in one rabbit. 2. Complete image series of a sagittal TSE T2w sequence in the same rabbit as in Additional file 1: 1. 3. Complete image series of a dorsal TSE T2w sequence in the same rabbit as in Additional file 1: 1. 4. Complete image series of a FLAIR longTR CLEAR sequence in the same rabbit as in Additional file 1: 1. 5. Complete image series of a precontrast T1w 3D (TFE SENSE) sequence in the same rabbit as in Additional file 1: 1. 6. Complete image series of a postcontrast T1w 3D (TFE SENSE) sequence in the same rabbit as in Additional file 1: 1. [file 13028_2015_139_MOESM1_ESM.zip › Brain_2200809/sT1W_3D_TFE_701/IM-0005-0079.jpg]

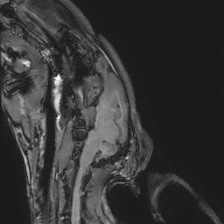

Supplement: Additional file 1: — 1. Complete image series of a transverse TSE T2w sequence in one rabbit. 2. Complete image series of a sagittal TSE T2w sequence in the same rabbit as in Additional file 1: 1. 3. Complete image series of a dorsal TSE T2w sequence in the same rabbit as in Additional file 1: 1. 4. Complete image series of a FLAIR longTR CLEAR sequence in the same rabbit as in Additional file 1: 1. 5. Complete image series of a precontrast T1w 3D (TFE SENSE) sequence in the same rabbit as in Additional file 1: 1. 6. Complete image series of a postcontrast T1w 3D (TFE SENSE) sequence in the same rabbit as in Additional file 1: 1. [file 13028_2015_139_MOESM1_ESM.zip › Brain_2200809/sT1W_3D_TFE_701/IM-0005-0080.jpg]
